# Reactive oxygen species stimulate Paneth cell plasticity and diminish antimicrobial function

**DOI:** 10.1038/s44318-026-00869-y

**Published:** 2026-07-18

**Authors:** Jiaxing Yang, Sheila Bandyopadhyay, Dahui Wang, Edward Gonzalez, Yeqing Chen, Jagannatham Naidu Bhupana, Oscar Pellon-Cardenas, Helen Park, Iyshwarya Balasubramanian, Jared Bianchi-Smak, Xiao Zhang, Zhi Wei, Edward M Bonder, Michael P Verzi, Jean-Pierre Etchegaray, Nan Gao

**Affiliations:** 1 Department of Biological Sciences, Rutgers University, Newark, NJ USA; 2 Department of Computational Sciences, New Jersey Institute of Technology, Newark, NJ USA; 3 Cooper Medical School of Rowan University and Cooper University Health Care, Camden, NJ USA; 4 Department of Pharmacology, Physiology, and Neurosciences, New Jersey Medical School, Rutgers University, Newark, NJ USA; 5 Department of Genetics, Rutgers University, New Brunswick, NJ USA; 6 Center for Immunity and Inflammation, New Jersey Medical School, Rutgers University, Newark, NJ USA

**Keywords:** Microbiology, Virology & Host Pathogen Interaction, Signal Transduction, Stem Cells & Regenerative Medicine

## Abstract

The gut epithelium faces constant environmental oxidative stresses. How it responds to pathological reactive oxygen species (ROS) is not fully understood. Using Paneth cell (PC) lineage reporters and genetic tracing, we show that as PC progenitors mature, a decline in their oxidative phosphorylation (OXPHOS) activity coincides with an increased capacity to produce antimicrobial peptides (AMPs). Direct stimulation by elevated extracellular ROS, often found during gut infection and inflammation, can drive a subset of PCs into mitotic cell division. Increasing mitochondrial superoxide production via impairment or uncoupling of the respiratory electron transport chain similarly stimulated PC cell-cycle entry and dedifferentiation into various intestinal epithelial cell types. Using gain- and loss-of-function approaches, we found that the Rho-GTPase CDC42 in PCs regulates the balance of ROS and antimicrobial peptide production, influencing the outcome of pathogenic infection. CDC42 deficiency impairs mitochondrial respiration in PCs, elevating cellular ROS, plasticity, and inflammation. Together, ROS directly suppress the mature PC phenotypes, contributing to the widely observed PC pathology in inflammatory diseases. Our findings suggest that molecular defects affecting OXPHOS balance and antimicrobial peptide production in PCs will likely impair innate defense and drive inflammatory progression in the intestine.

## Synopsis

Mature Paneth cells support intestinal stem cell renewal and produce antimicrobial peptides during homeostasis, and are often lost during gut inflammation and infection. Here, reactive oxygen species (ROS) produced by mitochondrial oxidative phosphorylation are found to regulate Paneth cell differentiation and their antimicrobial function.

- During Paneth cell maturation, OXPHOS activity decreases concomitantly with the increased expression of antimicrobial peptides.
- Exogenous ROS or pathological mitochondrial overproduction of ROS promote Paneth cell entry into the cell cycle and dedifferentiation in enteroids.
- Paneth-cell-specific loss of *Cdc42* elevates intracellular ROS, suppresses antimicrobial peptide production, and increases Paneth cell plasticity in vivo.

## Abstract (web summary)

The Rho GTPase CDC42 influencing the outcome of pathogenic infection in the intestine by regulating the balance of ROS and antimicrobial peptide production in Paneth cells.

## Graphical abstract

**Figure:**
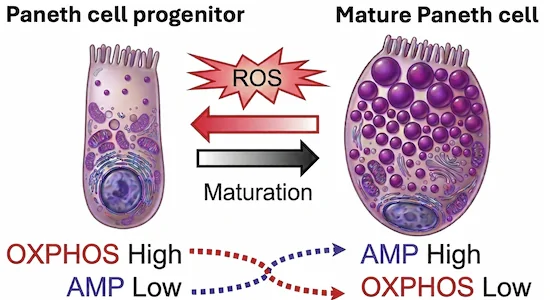

**Figure:**
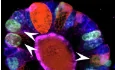

## Introduction

Mucosal tissues such as the gut are constantly exposed to oxidative stresses following exposure to various environmental challenges. In particular, infection and inflammatory responses lead to massive production of reactive oxygen species (ROS) from immune and non-immune cells within the gut mucosa. While physiological levels of ROS, e.g., hydrogen peroxide (H_2_O_2_), superoxide anion (O_2_^-^), and hydroxyl radicals (•OH), are made as metabolic byproducts primarily from oxidative respiration, pathological amounts of ROS can harm tissues by oxidizing macromolecules including nucleic acids, causing detrimental cell death and inflammatory disorders (Ramani et al, 2020). Cellular mechanisms that neutralize ROS damage include antioxidant and scavenger systems that remove oxidative damage and reparative pathways that restore normal cellular functions (Panday et al, 2015). In the fly and mouse gut, Keap1 and Nrf2 establish a critical redox management system to regulate intestinal stem cell (ISC) functions; loss of the management mechanism results in accumulation of ROS, causing epithelial degeneration (Circu and Aw, 2012; Hochmuth et al, 2011).

How different intestinal cell types respond to and manage oxidative stress is crucial for understanding the underlying mechanism guiding gut homeostasis and health. Commensal bacteria have been shown to increase ROS production in intestinal epithelial cells (IECs), which use ROS as physiological signaling molecules to regulate epithelial cell growth, proliferation, migration, and tissue integrity (Kumar et al, 2007; Lin et al, 2009; Swanson et al, 2011). Using a mouse intestinal organoid model, a mitochondrial ROS signaling mechanism was proposed to promote the differentiation of crypt-based columnar intestinal stem cells (ISCs) and drive formation of crypts (Rodriguez-Colman et al, 2017). This elegant work showed that PCs produce and export lactate, which is then used by neighboring Lgr5^+^ ISCs to support self-renewal. During infectious and non-infectious gut inflammation, pathological levels of ROS are produced and accumulated by immune and epithelial cells due to impaired mitochondrial function and ER stress. How the key gut epithelial cell types directly respond to pathological ROS is yet to be fully appreciated.

PCs are a gut secretory epithelial cell type that is differentiated from ISCs and regulates stem cell homeostasis, microbial community, and mucosal immunology (Adolph et al, 2013; Clevers and Bevins, 2013; Lueschow and McElroy, 2020; Sato et al, 2011). The loss or reduction in the PC population is frequently observed and reported across numerous intestinal diseases including Crohn’s disease (CD) (Adolph et al, 2013) (Wehkamp and Stange, 2020) (Cadwell et al, 2008; Liu et al, 2014; VanDussen et al, 2014) (Liu et al, 2016), graft-versus-host disease (GvHD) (Koreth, 2013; Levine et al, 2013), necrotizing enterocolitis (Coutinho et al, 1998; Lueschow and McElroy, 2020; McElroy et al, 2011), as well as in conditions challenged by environmental risk factors like cigarette smoking (Liu et al, 2018) or Western diet (Liu et al, 2021; Verhagen et al, 2024). Reduction in the mature PC population is often linked to increased disease severity, less therapeutic response, and higher mortality due to non-relapse causes, e.g., in GvHD patients (Koreth, 2013; Levine et al, 2013) or more rapid disease recurrence after surgery, e.g., in CD patients (VanDussen et al, 2014). PCs secrete antimicrobial peptides (AMPs) that target commensal microbes and foreign pathogens (Brandl et al, 2007; Han et al, 2024; Petnicki-Ocwieja et al, 2009; Salzman, 2010; Timmermans et al, 2025; Wilson et al, 1999; Yu et al, 2020). Mice with defective PC AMP biology have higher susceptibility to enteric infections (Gaudino et al, 2021; Martinez Rodriguez et al, 2012; Matamouros and Miller, 2015; Salzman et al, 2003a; Salzman et al, 2003b; Wilson et al, 1999). Interestingly, infection and inflammation often cause disruption of mature PCs’ secretory and/or granular phenotypes (Martinez Rodriguez et al, 2012; Matamouros and Miller, 2015; Salzman et al, 2003a). Aggressive enteric pathogens, such as *S. Typhimurium*, are known to robustly stimulate ROS production in immune and non-immune cells, and these pathogens then also exploit host oxygen species for their survival, replication, and virulence (Dong et al, 2015; Wang et al, 2020; Winter et al, 2010).

We recently reported that enteric infections by various pathogens can lead to elevated oxidative stress in PCs (Balasubramanian et al, 2023), but the direct impacts of exogenous or mitochondrial-derived ROS on PCs have not been carefully investigated. Here, we report that stimulation by exogenous ROS or aberrantly produced intracellular ROS through mitochondrial dysfunction elevated the plasticity of PC population sub-types. A small subset can be activated for proliferation and dedifferentiation. PC oxidative stress negatively regulates antimicrobial gene expression and peptide production. One of the regulators of this balance is the Rho GTPase CDC42, loss of which dramatically impairs mitochondrial function and elevated ROS. This regulation of ROS-AMP balance may be especially important for PCs to maintain gut innate defense and support tissue repair.

## Results

### A temporal decline in oxidative phosphorylation is correlated with Paneth cell maturation

**Figure 1:**
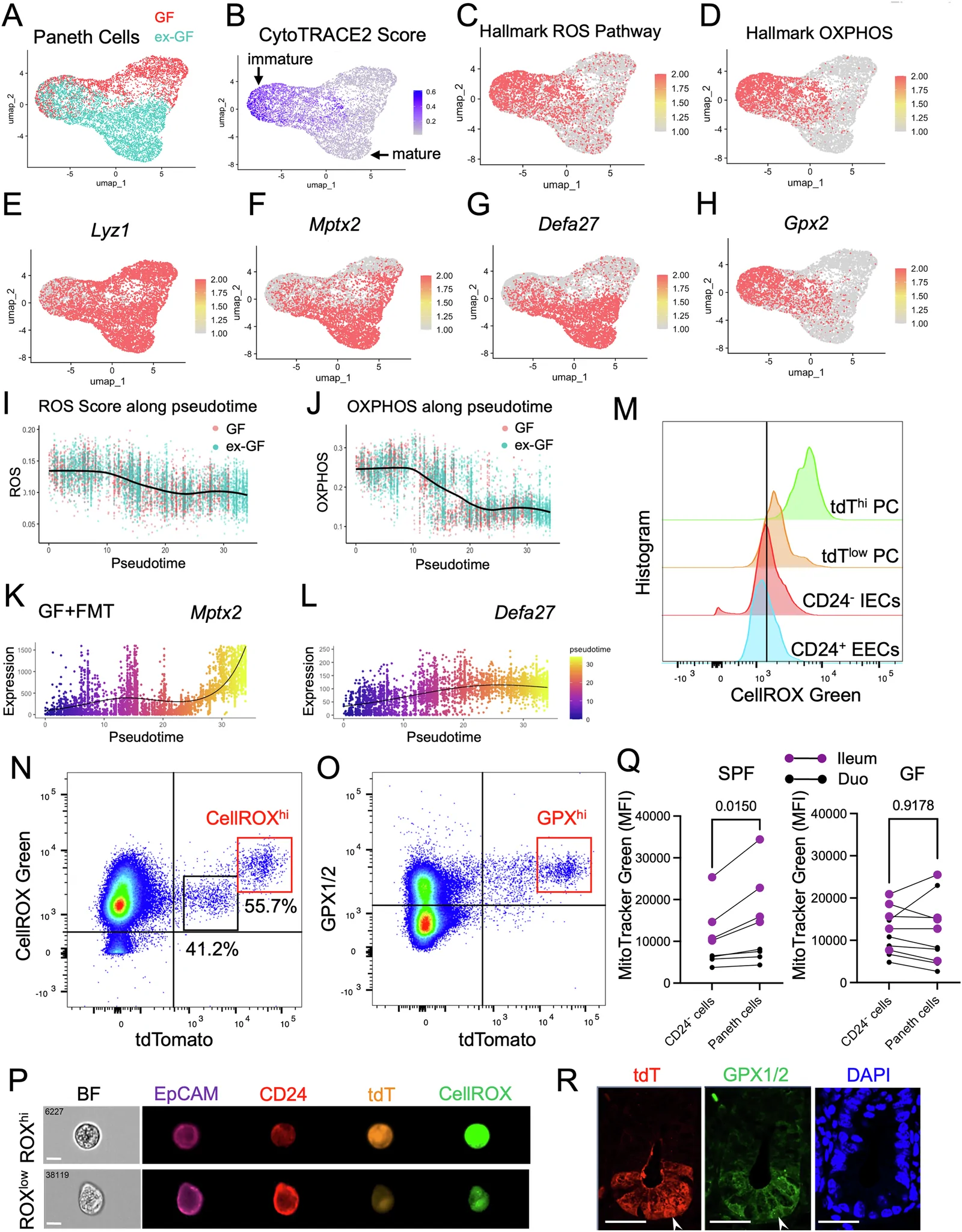
A temporal decline of oxidative phosphorylation during Paneth cell maturation. (**A**) Paneth cells (PCs) were isolated from the ilea of adult C57BL/6 germ-free (GF) PC reporter mice, and from ex-GF PC reporter mice colonized with the fecal microbiota of wild-type SPF C57BL/6 mice. scRNA-sequencing was performed. Uniform Manifold Approximation and Projection (UMAP) was used to resolve 5224 GF PCs (red) and 5646 ex-GF PCs (blue). (**B**) Cellular (Cyto) Trajectory Reconstruction Analysis using gene counts and expression was performed to determine the differentiation state of individual PCs from the scRNA-seq data. (**C**,** D**) UMAP was used to reveal the Hallmark gene sets for the reactive oxygen species (ROS) and oxidative phosphorylation (OXPHOS) pathways. (**E**–**H**) UMAP analysis was performed for *Lyz1*, *Mptx2*, *Defa27*, and *Gpx2*. (**I**,** J**) Hallmark ROS and OXPHOS gene sets were resolved along a pseudotime trajectory measuring the developmental transition of PCs from young progenitors to mature cells. (**K**,** L**) Pseudotime trajectory analysis was used to resolve *Mptx2* and *Defa27* expression in PCs. (**M**) Ileal EpCAM^+^ cells from PC reporter mice were segregated by flow cytometry into tdT^high^ and tdT^low^ CD24^+^ PCs, and CD24^+^ or CD24^-^ non-PCs. The X-axis represents CellROX intensity. (**N**, **O**) Flow cytometry for CellROX Green or GPX1/2 in tdT^+^ PCs gated from live ileal EpCAM^+^ cells from PC reporter mice. 55.7% of PCs exhibited higher CellROX levels (red box), and PCs with elevated GPX1/2 are boxed in panel O. This observation was consistent across five independent mice. (**P**) ImageStream analysis was used to visualize distinct CellROX intensities in individual PCs. Scale bar, 10 µm. (**Q**) Flow analysis comparing MitoTracker Green intensity in PCs and CD24^-^ non-PCs from the ileum (purple dots) or duodenum (black dots) of individual animals under SPF or GF conditions. *n* = 4 per condition. A paired Student’s *t*-test was used for the comparison. Exact *p*-values are provided above the comparison bars. (**R**) Confocal immunofluorescence analysis for GPX1/2 (green) and tdT (red) was performed on WT mouse small intestinal sections. DAPI labels the nuclei. The white arrow indicates a Paneth cell expressing GPX1/2. Scale bar, 20 µm. Source data are available online for this figure.

**Figure EV1:**
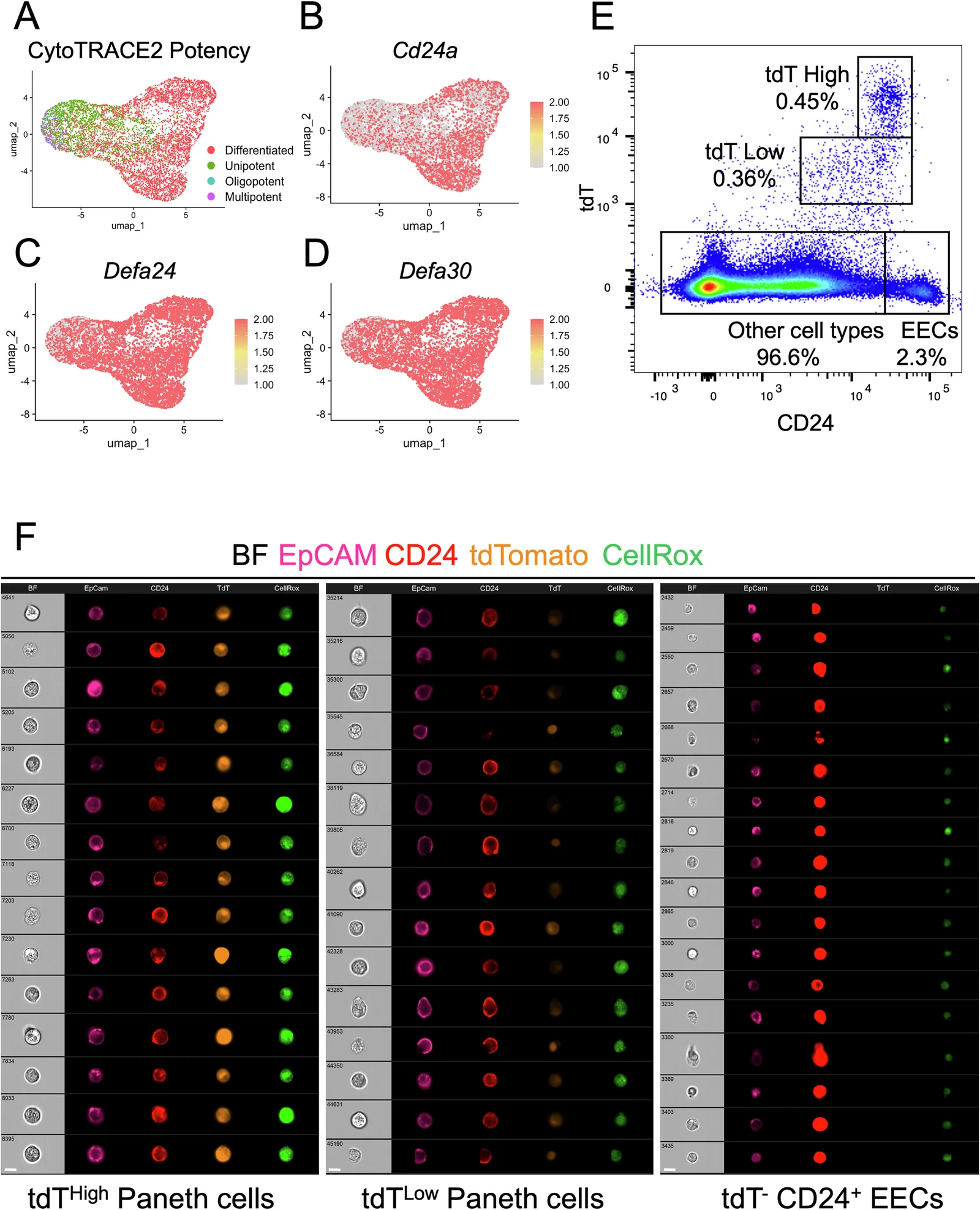
Intracellular ROS levels vary across Paneth cell populations. (**A**) CytoTRACE potency was calculated for individual Paneth cells. (**B**–**D**) UMAP for Cd24a, Defa24, and Defa30. (**E**) Live intestinal epithelial cells were resolved by the expression of tdT and CD24. (**F**) Imagestream analysis for CellROX was performed for tdT^High^ and tdT^Low^ Paneth cells, tdT^-^CD24^+^ EECs and tdT^-^CD24^-^ IECs. Scale bar, 10 µm.

Commensal microbiota promotes physiological ROS production in IECs (Alam et al, 2014; Kumar et al, 2007; Lin et al, 2009; Swanson et al, 2011). We first examined the single-cell transcriptomes of PCs isolated from adult *Lyz1*^3’UTR-IRES-CreER^; *Rosa26*R^tdTomato^ mice (hereafter called PC reporter mice) maintained under germ-free (GF) conditions or conventionalized (ex-GF) via a fecal microbiota transfer (FMT) from wild-type (WT) C57BL/6 mice. These PC reporter mice exhibit recombination in virtually all PCs across the proximal-to-distal axis of the small intestines (Balasubramanian et al, 2023). A single injection of tamoxifen was administered to induce the tdTomato (tdT) reporter expression in these mice. After 48 h, tdT^+^ crypt epithelial cells were isolated for scRNA-seq analysis (Fig. 1A). CytoTRACE2 (Kang et al, 2025) was used to compute potency scores for each PC, enabling identification of progenitor and differentiated clusters (Figs. 1B and EV1A). Pathway activity scores were computed using AUCell (Aibar et al, 2017) with hallmark gene sets from the Molecular Signatures Database (MSigDB) (Liberzon et al, 2015), revealing that PC progenitors have higher hallmark ROS signaling and oxidative phosphorylation (OXPHOS) transcriptomic signatures than mature PCs (Fig. 1C,D). UMAP plots of *Lyz1, Mptx2, Defa27, Cd24a, Defa24*, and *Defa30* illustrated the differential expression of AMPs  and Paneth cell markers across progenitor and mature PCs in GF and ex-GF mice (Figs. 1E–G and EV1B–D). The immature PCs express high levels of *Gpx2* (Fig. 1H), a gene which encodes the glutathione peroxidase that catalyzes the reduction of hydrogen peroxide (H_2_O_2_) and organic hydroperoxides by glutathione. The differences between mature and progenitor PCs in ROS and OXPHOS transcriptomes do not differ in GF or ex-GF mice, indicating a microbe-independent intrinsic feature.

Trajectory analysis was performed using monocle3 (Trapnell et al, 2014), with the PC progenitor cluster set as the starting root of the pseudo-time axis. A generalized linear model was then fitted for each gene to identify those whose expression levels significantly covaried with pseudo-time. These analyses revealed a temporal decline in ROS and OXPHOS hallmark gene signatures (Fig. 1I,J), which are tracking inversely with the increased AMP genes (Fig. 1K,L). *Mptx2* is a PC specific mucosal pentraxin gene identified by this pseudotime analysis as becoming most abundant in fully matured PCs in the ex-GF mice (Fig. 1F,K).

To validate these findings at the molecular level, flow cytometric analysis for CellROX was performed on PCs isolated from the reporter mice. CellROX, when oxidized in cells, becomes brightly fluorescent, allowing sensitive measurement of intracellular oxidative stress. Compared to a subset of CellROX^high^ PCs, CD24^+^ enteroendocrine cells (EECs) have the lowest CellROX activity (Figs. 1M and EV1E). Approximately 55.7% of PCs possess the higher CellRox (Fig. 1N). These PCs also contained higher GPX1/2 proteins (Fig. 1O). Image-stream analysis validated the existence of distinct CellROX abundances within the PC population, with the CellROX^low^ PCs frequently packaged with secretory granules (Figs. 1P and EV1F).

As mitochondria contribute up to 90% of intracellular ROS, we analyzed mitochondrial abundance by MitoTracker Green in live PCs isolated from SPF or GF mice. SPF PCs contain moderately more mitochondria than CD24^-^ non-PCs, and this difference is more pronounced in the ileum of SPF mice (Fig. 1Q). These differences were not observed in GF mice.

Immunostaining for GPX1/2 revealed that these enzymes are concentrated at the crypt base in a subset of PCs (Fig. 1R), in agreement with previous reports in mice (Florian et al, 2010; Komatsu et al, 2001), and in humans (Florian et al, 2001). Thus, the PC population has an intrinsic developmental variation in oxidative phosphorylation, which is likely modifiable by the microbiota.

### Exogenous and mitochondrial ROS stimulate a subset of Paneth cells to enter the cell cycle

**Figure EV2:**
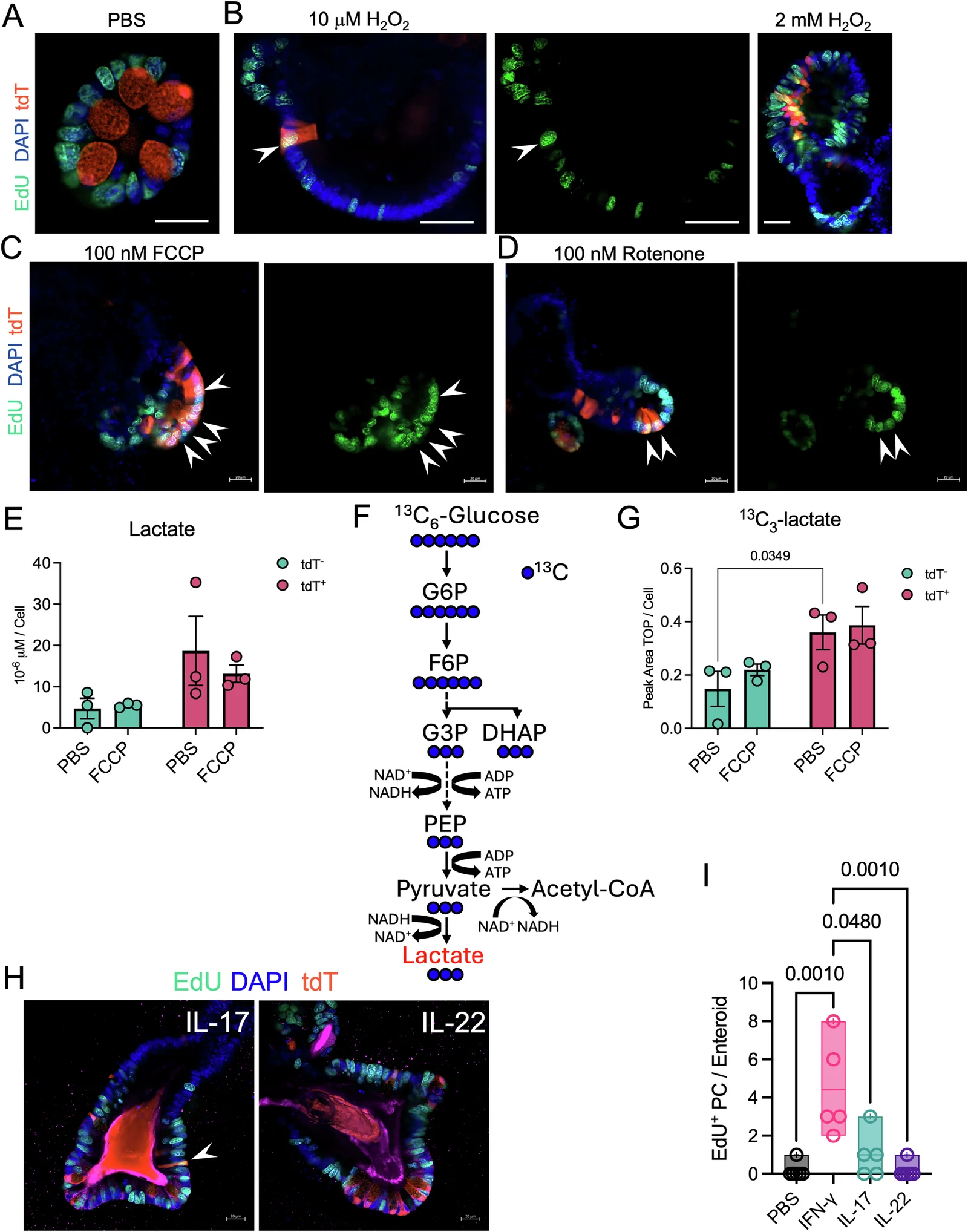
Impact of oxidative stress on cell-cycle entry and metabolism in Paneth cells. (**A**–**D**) Enteroids were grown from PC reporter mouse duodenum, induced for tdT expression on day 3, and treated with various ROS for 24 h. Enteroids were labeled with EdU for 1 h before fixation and imaging. Scale bar, 20 µm. Arrowheads point to EdU^+^tdT^+^ cells. Scale bar, 20 µm. (**E**) Approximately 1000 duodenal enteroids from PC reporter mice (*n* = 3) were treated with FCCP for 24 h, washed out, and 17.5 mM ^13^C_6_-glucose was added to the medium for a duration of 17 h before washing out and proceeding to FACS sorting. tdT^+^ and tdT^-^ IECs were isolated for LC-MS analysis. Lactate concentrations were normalized against the cell number of individual samples and plotted. Data are presented as mean ± SEM. (**F**) Schematic diagram illustrating the glycolysis of glucose to pyruvate, which is either converted to lactate through anaerobic respiration or enters mitochondria to fuel the TCA cycle. (**G**) Using samples from the experiment described in (**E**), ^13^C_3_-lactate abundance was quantified from individual samples treated with PBS or FCCP. A two-way ANOVA followed by Fisher’s LSD test was performed to compare the difference. Exact *p*-value is provided above the comparison bar. Data are presented as mean ± SEM. (**H**) Enteroids were grown from PC reporter mouse duodenum, induced for tdT expression on day 3, and treated with 50 ng/mL IL-17 or 4 ng/mL IL-22 for 24 h. Enteroids were labeled with EdU for 1 h before fixation and imaging. Scale bar, 20 µm. Arrowheads point to an EdU^+^tdT^+^ cell in the IL-17-treated condition. (**I**) The number of EdU^+^tdT^+^ cells was calculated for individual enteroids treated with PBS (*n* = 6), IFN-γ (*n* = 5), IL-17 (*n* = 6), or IL-22 (*n* = 5). Statistical comparisons between groups were performed using a Kruskal-Wallis test followed by an uncorrected Dunn’s multiple comparisons test. Exact *p*-values are provided above the comparison bars. Data are presented as floating bars (min to max) with the central line indicating the mean.

**Figure 2:**
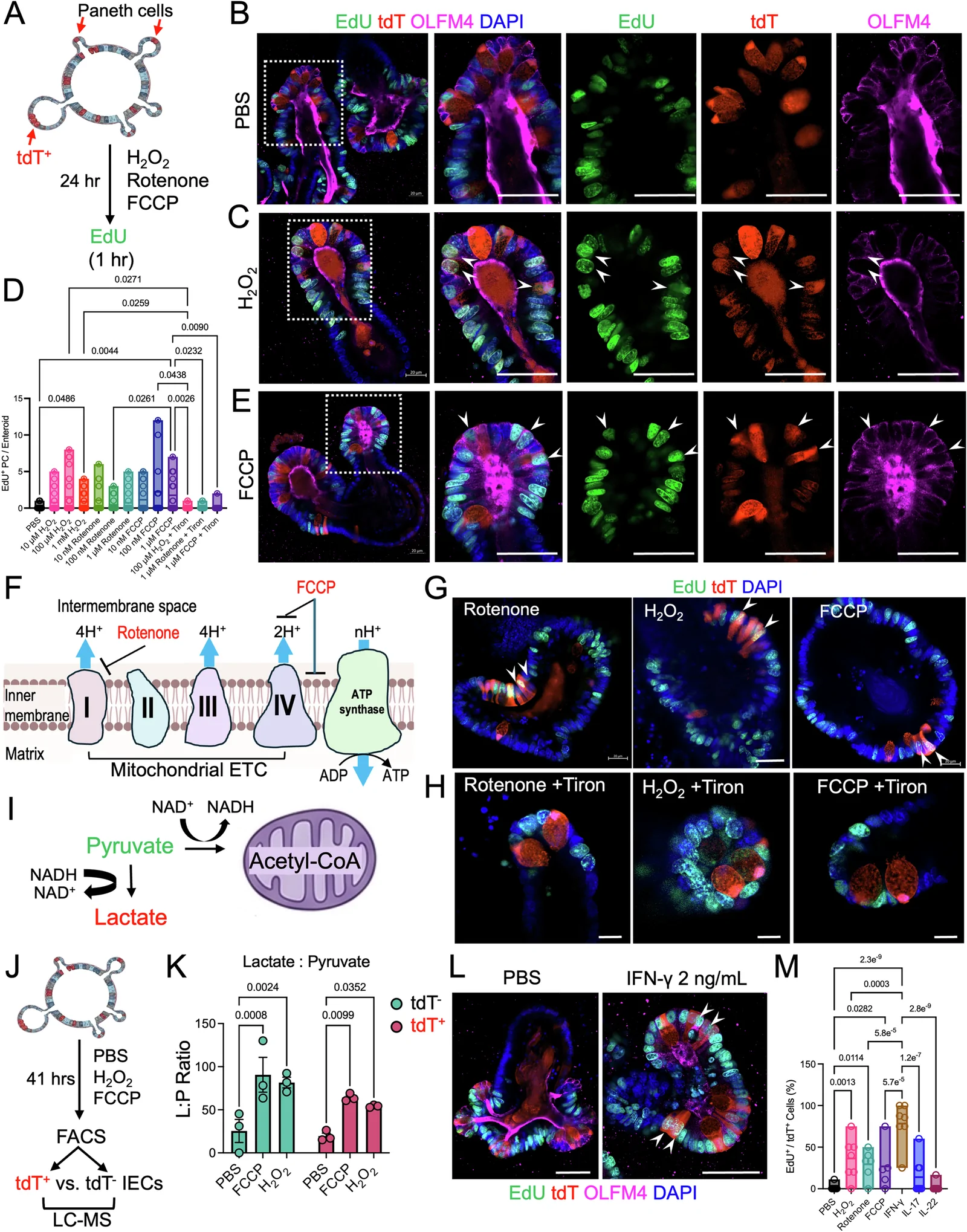
Exogenous H_2_O_2_ and mitochondrial ROS stimulate cell-cycle entry in a PC subset. (**A**) Enteroids were grown from adult PC reporter mouse duodenum. On day 3, 4-OHT was added to induce tdT expression in PCs. Two days later, enteroids were treated for 24 h with exogenous H_2_O_2_, rotenone (a mitochondrial electron transport chain [ETC] inhibitor), or FCCP (a mitochondrial OXPHOS uncoupler). Enteroids were labeled with EdU for 1 h piror to fixation. (**B**,** C**) Fixed enteroids were analyzed via confocal fluorescence microscopy for EdU, tdT, OLFM4, and DAPI. Arrowheads point to EdU^+^tdT^+^ cells in the H_2_O_2_-treated conditions. Scale bar, 20 µm. (**D**) The number of EdU^+^tdT^+^ cells was scored for individual enteroids across different treatment groups. Counts were obtained from 3 independent experiments. A Kruskal-Wallis test followed by an uncorrected Dunn’s test was performed to determine statistical significance. Exact *p*-values are provided above the comparison bars. Data are presented as floating bar (min to max) with line at mean. (**E**) Fixed enteroids were analyzed by confocal fluorescence microscopy for EdU, tdT, OLFM4, and DAPI. Arrowheads point to EdU^+^tdT^+^ cells in the FCCP-treated condition. Scale bar, 20 µm. (**F**) Schematic diagram illustrating the ETC targets inhibited by rotenone and FCCP. (**G**, **H**) Representative images of enteroids co-treated with ROS and Tiron, compared to treatment with ROS alone. Arrowheads point to EdU^+^tdT^+^ cells. Scale bar, (**G**) 20 µm, (**H**) 10 µm. (**I**) Schematic diagram illustrating the distinct metabolic fates of pyruvate: conversion into acetyl-CoA for the mitochondrial TCA cycle versus reduction to lactate during anaerobic glycolysis. (**J**) Enteroids were grown from adult PC reporter mouse duodenum with tdT expression induced on day 3 after seeding. They were treated with PBS, H_2_O_2_ or FCCP for 24 h followed by isotope labeling for 17 h. Live tdT^+^ and tdT^-^ cells were isolated via FACS and subjected to untargeted metabolomic analysis by LC-MS. Approximately 150,000 tdT^+^ and 300,000 tdT^-^ cells were collected per sample (*n* = 3 per condition). (**K**) The [lactate]:[pyruvate] ratio for different conditions was computed from the LC-MS results and compared using an ordinary two-way ANOVA multiple comparison. Exact *p*-values are provided above the comparison bars. Data are presented as mean ± SEM. Cells from approximately 1000 enteroids, isolated from three independent mice (*n* = 3), were analyzed per group. (**L**) Enteroids were grown from the duodenum of adult PC reporter mice. On day 3, 4-OHT was added to induce tdT expression in PCs. On day 4, 2 ng/mL IFN-γ was added, and washed out after 24 h. Enteroids were labeled with EdU for 1 h and analyzed by confocal fluorescence microscopy. Arrowheads point to EdU^+^tdT^+^ cells. Scale bar, 20 µm. (**M**) The percentage of EdU^+^ cells within the tdT^+^ population was calculated for individual enteroids treated with PBS (*n* = 8), H_2_O_2_ (*n* = 7), rotenone (*n* = 6), FCCP (*n* = 5), IFN-γ (*n* = 7), IL-17 (*n* = 8), or IL-22 (*n* = 8). Statistical comparisons between groups were performed using an ordinary one-way ANOVA followed by Fisher’s LSD test. Exact *p*-values are provided above the comparison bars. Data are presented as floating bars (min to max) with the central line indicating the mean. Source data are available online for this figure.

During infectious or non-infectious gut inflammation, pathological amounts of ROS made by immune cells (e.g., neutrophils) can accumulate at the crypt niche. To directly test the impact of exogenous ROS on PCs, we grew enteroids from the PC reporter mice and induced tdT expression 3 days after seeding. After 1–2 days, there were abundant tdT-labeled PCs in the enteroid buds, and they appeared large, round and packed with granules (Fig. EV2A). We treated the enteroids with H_2_O_2_ at different concentrations. Forman and colleagues analyzed the literature and the chemical kinetics, which suggested a normal range of H_2_O_2_ at 1–5 µM in human plasma, which could rise to 50 µM in inflammatory diseases (Forman et al, 2016). The cell or enteroid culture media are typically supplemented with antioxidants (e.g., for enteroid culture: N-acetylcysteine, vitamin E, superoxide dismutase, catalase and glutathione); thus, ex vitro studies often require a higher H_2_O_2_ to define its effects. Twenty-four hours after treating enteroids with H_2_O_2_, we added EdU and incubated the enteroids for 1 h to label the cycling cells (Fig. 2A). Enteroids were then fixed for confocal fluorescent analysis. We detected a small fraction of EdU^+^ tdT^+^ PCs at various H_2_O_2_ concentrations with 10 µM and 2 mM being the lowest and highest ones tested (white arrows in Figs. 2C and EV2B). These EdU^+^/tdT^+^ events appeared in 28%, 36%, and 38% in 10 µM, 100 µM, and 1 mM H_2_O_2_-treated enteroids analyzed in multiple experiments. Rarely any EdU^+^tdT^+^ PCs were detected in PBS-treated enteroids in the same experiments (Fig. 2B,D). Although 1 mM H_2_O_2_ significantly increased the overall frequency of EdU^+^tdT^+^ events compared to lower concentrations of H_2_O_2_, it did not significantly promote the total number of EdU^+^tdT^+^ cells per enteroid (Fig. 2D). No visible cell death was observed with concentrations of H_2_O_2_ less than 2 mM.

Compared to H_2_O_2_ that diffuses across cell membranes and travels long distances, superoxide anion (O_2_^-^) is charged and cannot readily cross membranes. Intracellular superoxide is primarily produced through electron leaks from the mitochondrial electron transport chain (ETC) during oxidative phosphorylation. PC progenitors exhibit relatively higher OXPHOS activity (Fig. 1D,J). We therefore asked whether an aberrantly elevated mitochondrial ROS production may have impact on PCs. Like the H_2_O_2_ experiments, PC reporter enteroids were treated for 24 h with carbonyl cyanide-p-trifluoromethoxyphenylhydrazone (FCCP), which uncouples the mitochondrial ETC from ATP synthesis (Cadenas, 2018) (Fig. 2E,F). Under this scenario where a maximized mitochondrial superoxide is produced from the still functioning ETC, we detected increased frequencies of EdU^+^tdT^+^ events in approximately 29%, 36%, and 50% of enteroids treated with 10 nM, 100 nM, and 1 µM FCCP (Figs. 2E and EV2C). Although 1 µM FCCP significantly increased the frequency of EdU^+^tdT^+^ cells, higher FCCP concentration did not increase the total number of EdU^+^tdT^+^ cells per enteroid compared to the lower concentration treatments (Fig. 2D). To investigate this further, we tested Rotenone, a mitochondrial ETC complex I inhibitor that blocks the electron transfer from iron-sulfur centers to ubiquinone (Fig. 2F), leading to electron buildup and ROS production (Li et al, 2003). Rotenone, at as low as 10 nM, was also sufficient to increase the frequency of EdU^+^tdT^+^ cells in enteroids within 24 h (Figs. 2D,G and EV2D). In general, these ROS-induced EdU^+^tdT^+^ cells took on a slender and tall columnar morphology, a feature different from the mature PCs.

To assess whether the observed cell-cycle entry in the above stimulated PCs was caused by the ROS activity, we co-treated enteroids with Tiron, a cell-permeable ROS scavenger and antioxidant (Oyewole et al, 2014). Tiron completely abolished the increased EdU^+^tdT^+^ events in H_2_O_2_-, FCCP- or Rotenone-treated enteroids (Fig. 2D,G,H), suggesting that these observed phenotypes were caused by oxidative reactivity.

Glycolysis metabolizes glucose to pyruvate in the cytosol for further oxidation in mitochondria or anaerobic conversion into lactate (Fig. 2I). Previous work has shown that PCs produce lactate to fuel the ISC self-renewal and differentiation (Rodriguez-Colman et al, 2017). To examine if ROS directly alters PCs’ conversion of pyruvate into lactate, we performed untargeted LC-MS metabolomic analysis on tdT^+^ PCs FACS-isolated from enteroids 17 h after a 24-h stimulation with H_2_O_2_ or FCCP (Fig. 2J). PBS-treated enteroids were used as a negative control. The results revealed that ROS treatment significantly elevated [lactate]:[pyruvate] (L:P) ratio in PCs and non-PCs (Fig. 2K). Due to the equilibrium of the reaction catalyzed by lactate dehydrogenase: [Pyr][NADH][H^+^]=K_eq_[Lac][NAD^+^], the L:P ratio reflects cellular energy metabolism, mitochondrial disorders, and the cellular redox state (NADH:NAD^+^). A higher L:P ratio suggests defects in the mitochondrial respiratory chain, which is expected in these enteroids treated with the FCCP or H_2_O_2_. Furthermore, the PCs generally possess higher concentrations of lactate than do non-PCs (Fig. EV2E). We performed a 17-h pulse-chase stable isotope tracing of ^13^C_6_-glucose added to enteroids under unchallenged vs. 24-h FCCP-treated conditions. The results suggest that the ^13^C_6_-glucose was exclusively used to produce ^13^C_3_-lactate in PCs (Fig. EV2F,G), while no tracer was detected in TCA metabolites in this experiment.

Several inflammatory cytokines have been shown to affect PC biology. IFN-γ was reported to induce PC extrusion and degranulation (Farin et al, 2014). IL-17 promotes PC dedifferentiation (Kempen et al, 2026) while IL-22 promotes PC differentiation (He et al, 2022). We asked if these cytokines may also promote PC cell-cycle entry. Treating enteroids with 2 ng/mL IFN-γ for 24 h caused a significantly higher frequency of EdU^+^tdT^+^ events than observed in PBS-treated enteroids (Fig. 2L,M), with the percentage of EdU^+^ cells out of the total tdT+ cells being significantly higher than that in FCCP-treated enteroids (Fig. 2M). IL-17 slightly elevated while IL-22 failed to increase EdU^+^tdT^+^ event frequency compared to the PBS-treated enteroids (Fig. EV2H,I).

### ROS stimulates the plasticity of a subset of PCs

**Figure 3:**
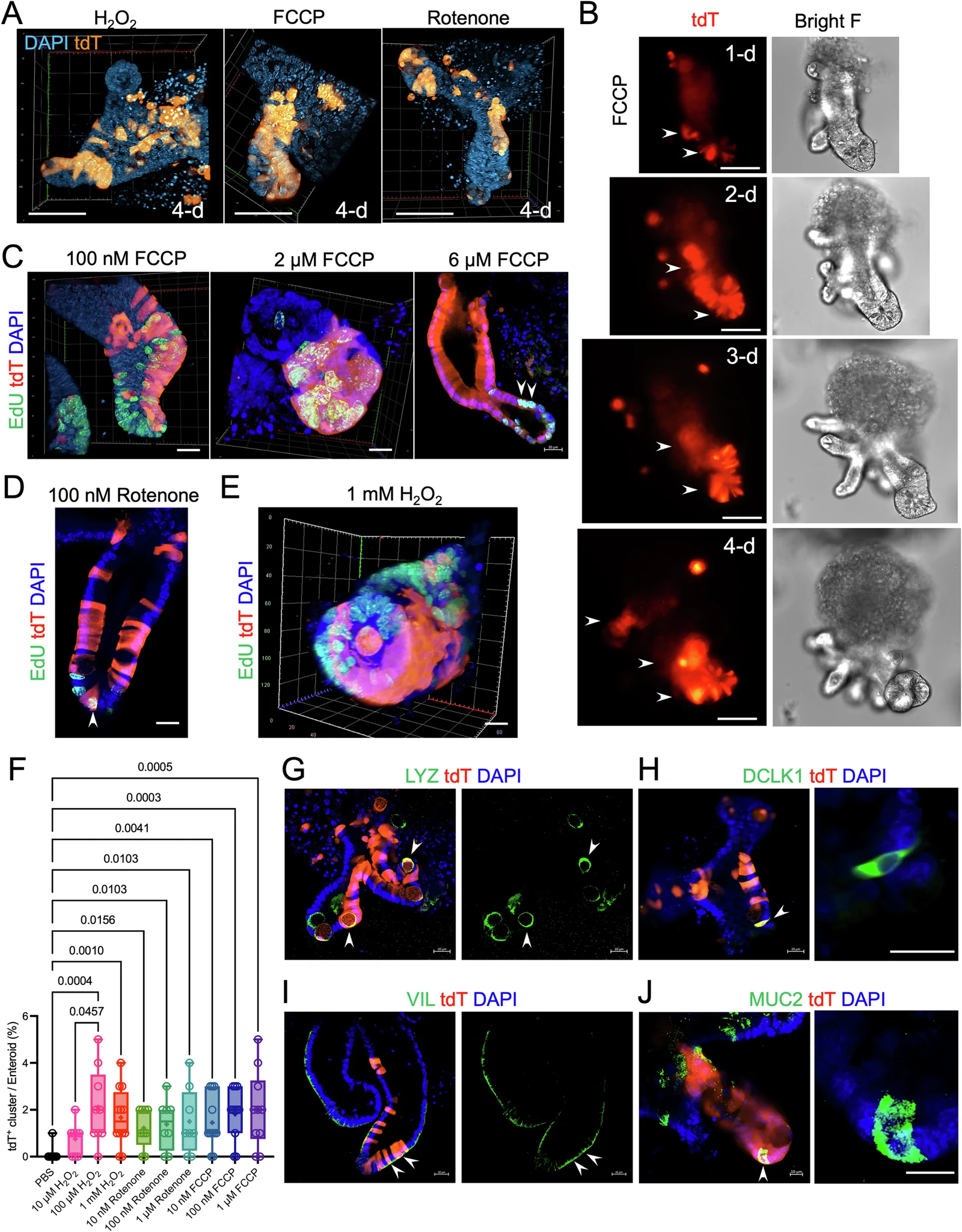
ROS stimulates dedifferentiation of a subset of Paneth cells. (**A**) Enteroids were grown from PC reporter mouse duodenum. On day 3 after seeding, 4-OHT was added to induce tdT expression. H_2_O_2_, or FCCP, or rotenone was added for 24 h, washed out, and then the enteroids were cultured for an additional 3 days before imaging. Representative 3-D projections were shown to illustrate the expansion of the red lineage tracing ribbons. Scale bar, 60 µm. (**B**) A series of time-course images were taken of FCCP-treated enteroids, from 1 to 4 days after a single 24 h treatment. tdT and bright field images were taken of the same enteroid to depict the progressive expansion of tdT^+^ progenies (arrowheads) originating from the PCs. Scale bar, 100 µm. (**C**) Representative images of red ribbons were taken of enteroids treated with 100 nM, 2 µM, and 6 µM FCCP. Scale bar, 20 µm. White arrows indicate EdU^+^tdT^+^ cells in the transit-amplifying zone. (**D**, **E**) Representative images of red ribbons were taken of enteroids treated with 100 nM rotenone or 1 mM H_2_O_2_. Scale bar, (**E**) 20 µm. (**F**) tdT cell clusters or ribbons (> 5 tdT^+^ cells in continuation) observed in enteroids under different treatments were scored as a percentage, from 3 independent experiments. A Kruskal-Wallis test followed by an uncorrected Dunn’s test was performed to determine the statistical significance. Exact *p*-values are provided above the comparison bars. Data are presented as box-and-whisker plot (box: 25th–75th percentiles; whiskers: min–max; center line: median). (**G**–**J**) ROS-stimulated enteroids were fixed at day 4, and co-stained for lysozyme, DCLK1, VILLIN and MUC2. Arrowheads point to tdT^+^ cells that co-expressed these individual markers. Scale bar, (**G**–**I**) 20 µm, (**J**) 10 µm. Source data are available online for this figure.

Forced cell-cycle entry may cause growth arrest and death of the PCs. We therefore investigated these ROS-induced events in time-course experiments to determine whether the observed EdU^+^tdT^+^ cells were lost over time or able to propagate like the de-differentiated PCs. We followed the ROS-treated enteroids in culture for 4 days and observed expansions in a subset of the lineage-traced tdT^+^ PC progenies under all 3 treatments (Fig. 3A). We noted that most of the lineage tracing and tdT^+^ transit amplification happened between the second and third days after ROS treatment (Fig. 3B). Lineage ribbons were detected in response to a wide range of concentrations of FCCP at 100 nM up to 6 µM (Fig. 3C), to which the enteroids showed an overall tolerance. Similar ribbons were observed in rotenone- (Fig. 3D) and H_2_O_2_-treated enteroids (Fig. 3E). We noted that the tdT^+^EdU^+^ cells were localized in the transit-amplifying zone (arrows in Fig. 3C) or at the bottom of the crypts (arrow in Fig. 3D), suggesting that these de-differentiated PCs may be the sources giving rise to the lineage-tracing stripes. Enteroids can tolerate up to 2 mM H_2_O_2_ and 2 µM Rotenone, with higher concentrations inducing cell deaths. Across multiple independent experiments, the frequency of observing these lineage-tracing tdT^+^ clusters or ribbons ranged from 1% to 5% of the enteroids, which was significantly higher than that in unchallenged enteroids (Fig. 3F).

Co-staining these tdT^+^ lineage-traced events with cell-type-specific markers revealed that the majority of the villus-localized tdT^+^ cells do not express lysozyme (Fig. 3G), but instead expressed markers of Tuft cells (DCLK1) (Fig. 3H), enterocytes (VIL) (Fig. 3I), and goblet cells (MUC2) (Fig. 3J). The kinetics of these lineage tracing events suggest a rapid ROS-induced cell-cycle entry in a subset of PCs within the first 24 h followed by a transit-amplification and differentiation in a fraction of these activated cells 2 to 3 days following the treatment.

**Figure EV3:**
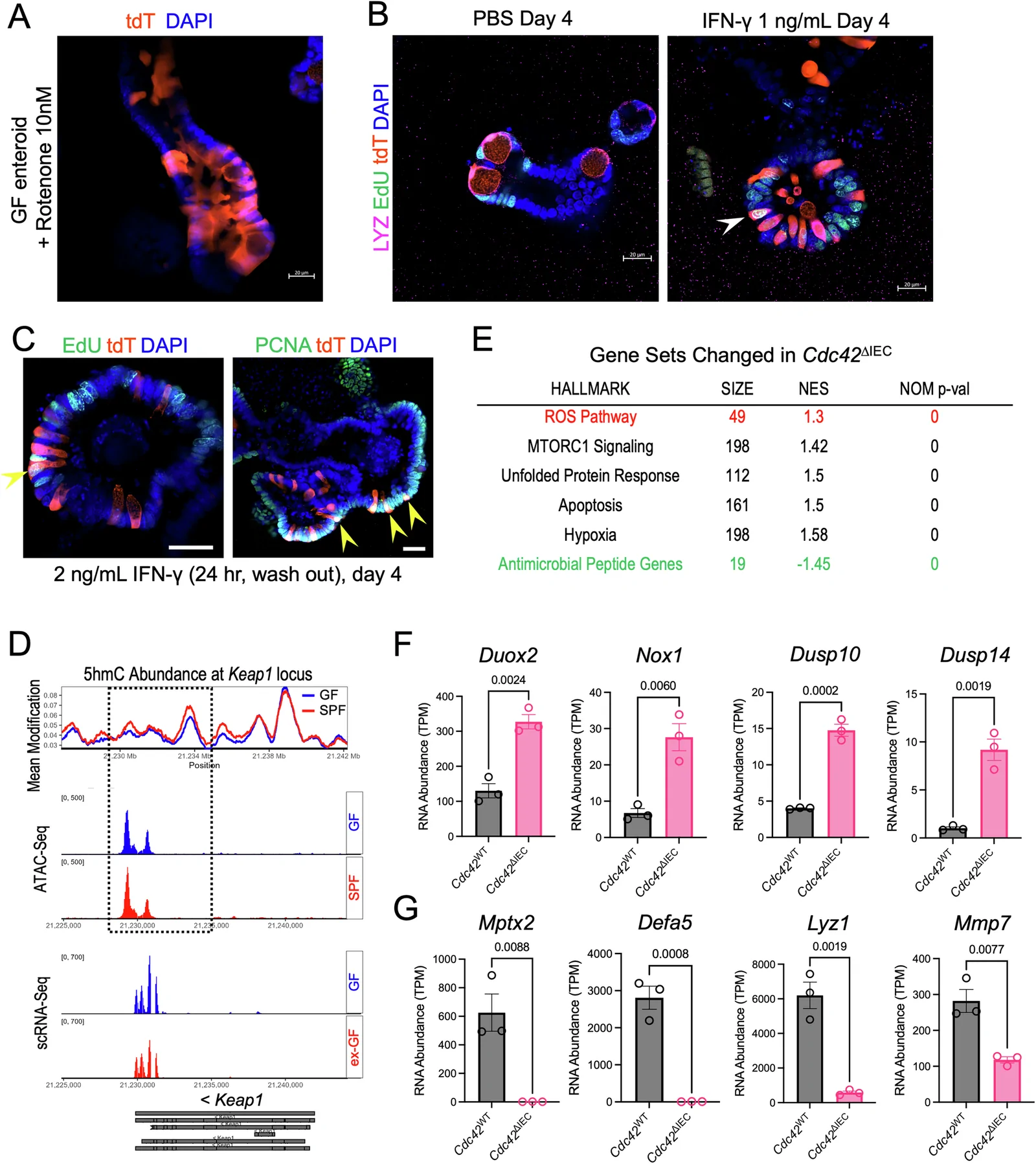
*Cdc42* deficiency upregulates the ROS pathway and suppresses the AMP gene program. (**A**) Enteroids were grown from GF Paneth cell reporter mice. On day 3, 4-OHT was added, and on the following day enteroids were treated with 10 nM rotenone. The image was taken of an enteroid that showed a tdT^+^ stripe. Scale bar, 20 µm. (**B**) Enteroids from Paneth cell reporter mouse duodenum were continuously treated with 1 ng/mL IFN-γ or PBS for 4 days, labeled with EdU 1 h before fixation, and stained for lysozyme, EdU, tdT, and DAPI. The white arrow indicates a LYZ^+^EdU^+^tdT^+^ cell. Scale bar, 20 µm. (**C**) Enteroids from Paneth cell reporter mouse duodenum were treated with 2 ng/mL IFN-γ for 24 h, washed out, then cultured to day 4, labeled with EdU for 1 h, fixed and stained for lysozyme, EdU, tdT, and DAPI; or PCNA, tdT, and DAPI. Yellow arrows indicate PCNA^+^tdT^+^ cells. Scale bar, 20 µm. (**D**) Paneth cell specific multi-modality omics for 5hmC DNA nanopore-sequencing, ATAC-seq and scRNA-seq of Paneth cells from GF and SPF PC reporter mice. (**E**) Bulk RNA-seq was performed using WT vs. *Cdc42*^ΔIEC^ mouse small intestinal epithelia (*n* = 3 per condition). Hallmark gene sets for reactive oxygen species (red) and antimicrobial peptides (green) are significantly increased and decreased in *Cdc42*^ΔIEC^ mice, respectively. (**F**) RNA abundances of ROS production and regulatory enzymes in *Cdc42*^WT^ (*n* = 3) and *Cdc42*^ΔIEC^ (*n* = 3) mice are shown. A two-tailed Student’s *t*-test was used to compare the differences. Exact *p*-values are provided above the comparison bars. Data are presented as mean ± SEM. (**G**) RNA abundances of AMP genes in *Cdc42*^WT^ (*n* = 3) and *Cdc42*^ΔIEC^ (*n* = 3) mice. A two-tailed Student’s *t*-test was used to compare the differences. Exact *p*-values are provided above the comparison bars. Data are presented as mean ± SEM.

We performed similar experiments in enteroids grown from GF PC reporter mice and were able to observe similar lineage tracing events with a clearly reduced frequency by approximately 67% (Fig. EV3A), suggesting that microbiota may not be required yet could modify the frequency of these ROS-induced events. Treating enteroids with 1 ng/mL IFN-γ continuously for 4 days caused death of 70% of enteroids. Almost all surviving IFN-γ-treated enteroids contained EdU^+^tdT^+^ cells, but none of these tdT^+^ cells formed lineage-tracing ribbons (Fig. EV3B), as seen in ROS-treated enteroids. Pulse treatment of enteroids with 2 ng/mL IFN-γ for 24 h, wash-out, and continued culture up to 4 days did not induce lineage-tracing ribbons either, and this was confirmed by staining for EdU or PCNA (Fig. EV3C).

### CDC42 is a regulator of cellular oxidative stress

**Figure 4:**
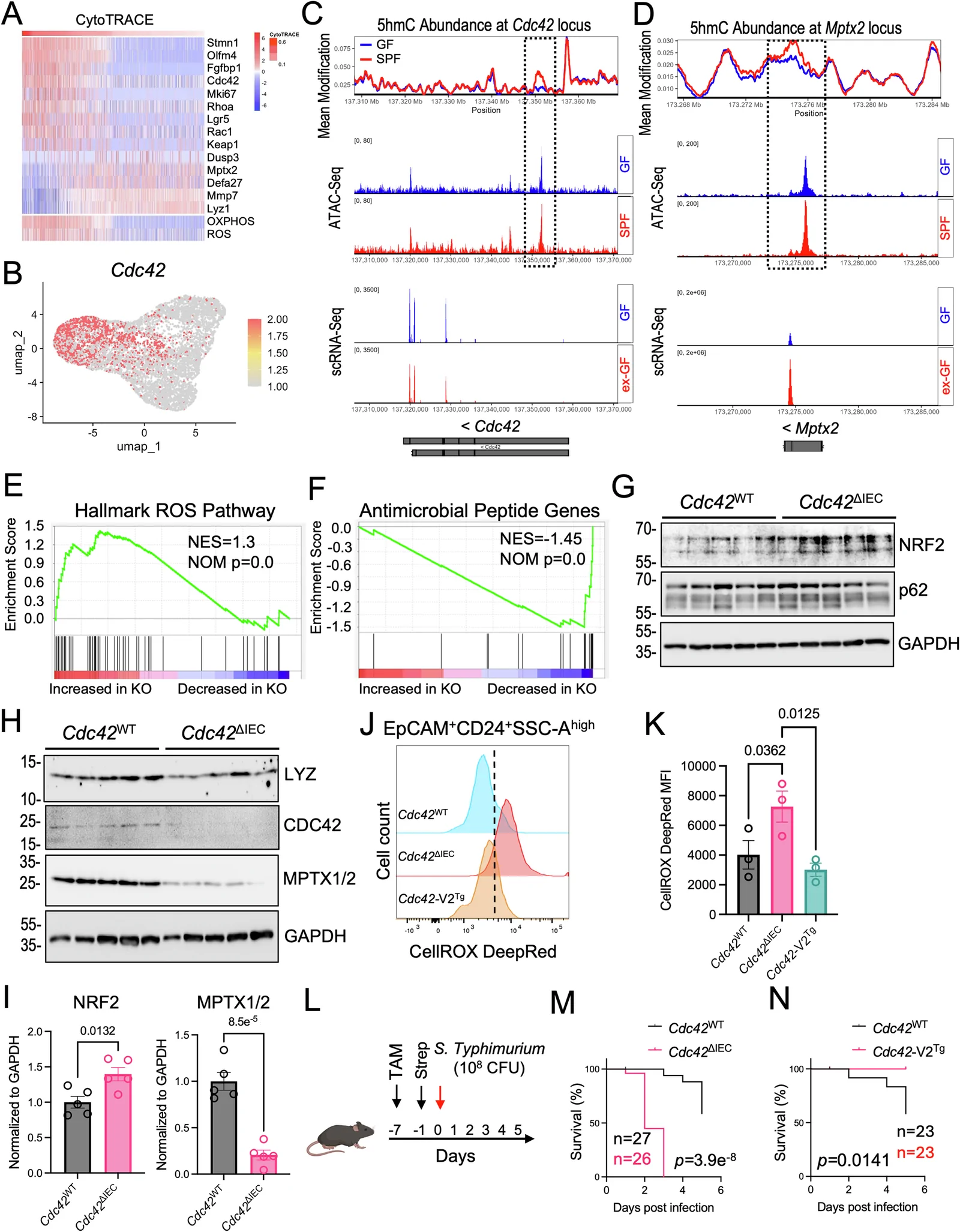
Cdc42 is a ROS and AMP regulator. (**A**) CytoTRACE2 v1.0 was used to compute potency scores for each cell, enabling the identification of progenitor and mature PC clusters. A generalized linear model was fitted for each gene to identify those whose expression levels significantly covaried with pseudo-time. Reactive oxygen species and OXPHOS pathway activity scores were computed using AUCell v1.26 with hallmark gene sets from the Molecular Signatures Database (MSigDB). The endogenous ROS inhibitor Cdc42 is among multiple ISC genes and ROS machinery genes (e.g., Rac1, Keap1, Dusp3). (**B**) UMAP was used to visualize Cdc42 expression across Paneth cell populations. (**C**,** D**) Paneth cells were isolated from GF or SPF PC reporter mouse ilea for analysis of 5hmC DNA modifications and chromatin status via nanopore sequencing and ATAC-Seq. PC specific multi-modality omics profiles at the *Cdc42* (**C**) and *Mptx2* (**D**) gene loci are shown. Boxed regions illustrate correlations of peaks for 5hmC modifications and chromatin accessibility. For ATAC-seq, *n* = 4 for SPF mice and *n* = 3 for GF mice. For nanopore sequencing, *n* = 4 for SPF mice and *n* = 5 for GF mice. (**E**, **F**) Bulk RNA-seq was performed using *Cdc42*^WT^ vs. *Cdc42*^ΔIEC^ mouse small intestines (*n* = 3 per condition). GSEA for the hallmark ROS pathway and for the antimicrobial peptide gene (AMP) pathway was plotted, and the normalized enrichment score (NES) and nominal *p*-values are shown. An empirical phenotype-based permutation test was used to estimate nominal *p*-values. (**G**,** H**) Western blots were performed for protein targets related to the ROS pathway and AMP products of PCs from *Cdc42*^WT^ and *Cdc42*^ΔIEC^ intestines (*n* = 5 mice per genotype). (**I**) A Student’s *t*-test was used to compare the protein abundances in panels G and H as quantified by ImageJ. Exact *p*-values are provided above the comparison bars. Data are presented as mean ± SEM. (**J**,** K**) Flow cytometry for CellROX was performed on ileal PCs gated as EpCAM^+^CD24^+^SSC-A^high^, from *Cdc42*^WT^, *Cdc42*^ΔIEC^, and *Cdc42-*V2^Tg^ mice. *n* = 3 mice per genotype. Statistical comparisons between groups were performed using an ordinary one-way ANOVA followed by Fisher’s LSD test. Exact *p*-values are provided above the comparison bars. In (**K**), data are presented as mean ± SEM. (**L**) Mice (all littermates) were injected with tamoxifen. The next day, mice were gavaged with streptomycin and, one day later, inoculated with 10^8^ colony forming units (CFU) of *S. Typhimurium*. All mice were euthanized by 5 d.p.i. (**M**) Survival of *Cdc42*^WT^ (*n* = 27) and *Cdc42*^ΔIEC^ (*n* = 26) littermates following the *S. Typhimurium* infection. Note: When the body weight of any given mouse dropped 20% or more from the initial weight, the mouse was humanely euthanized and counted as a death. This was applied across all genotypes. Survival analysis and a log-rank test were performed. (**N**) Survival of *Cdc42*^WT^ (*n* = 23) and *Cdc42-*V2^Tg^ (*n* = 23) littermates following the *S. Typhimurium* infection. Survival analysis and the individual uncorrected log-rank test were performed. Source data are available online for this figure.

We next searched for intrinsic mechanisms that may manage oxidative stress in Paneth cells. Ranking genes using correlation scores for CytoTRACE and pseudotime based on the single cell transcriptome, we found that ROS-regulatory genes, such as *Rac1, Keap1, Dusp3*, and *Cdc42*, having the highest correlation scores (Fig. 4A). UMAP shows that *Cdc42* is enriched in the progenitor PCs (Fig. 4B). Using PC-specific nanopore DNA sequencing and Assay for Transposase-Accessible Chromatin (ATAC)-Seq, we determined the genome-wide DNA modification by 5-hydroxymethylcytosine, an oxidative mark on cytosine that regulates chromatin accessibility and gene transcription (Gonzalez et al, 2023). These multi-modality assays on isolated PCs from GF and SPF mice revealed that, at the *Cdc42* gene locus, the 5-hydroxymethylcytosine sites are aligned with the open chromatin status revealed by ATAC-Seq (Fig. 4C). Similar correlative marks were found at the loci of *Mptx2* (Fig. 4D) and *Kelch-like ECH-associated protein 1 (Keap1)* (Fig. EV3D), a negative regulator of NRF2’s antioxidant response.

Loss of CDC42 has been shown to increase cellular ROS in immune and non-immune cell types in vivo (Diebold et al, 2004; Lee et al, 2013; Tackenberg et al, 2021; Zheng et al, 2024). We and others have reported that in unchallenged IEC-specific *Cdc42* knockout mice, PCs are mis-localized to the villus compartment (Melendez et al, 2013; Sakamori et al, 2012; Zhang et al, 2020), indicating a potential regulation of PC maturation and plasticity by CDC42. Bulk RNA-Seq of *Cdc42*^ΔIEC^ mouse IECs revealed a significant increase in the hallmark ROS signaling pathway gene signature (Figs. 4E and EV3E), including *Duox2, Nox1* (ROS generators), *Dusp10*, and *Dusp14* (ROS-regulating enzymes) (Fig. EV3F). In contrast, the AMP-related genes, e.g., *Mptx2*, *Lyz1*, *matrix metallopeptidase-7* (*Mmp7)*, and *α-defensins*, were diminished in *Cdc42*^ΔIEC^ mice (Figs. 4F and EV3E,G). Protein level analysis validated these ROS and AMP changes (Fig. 4G–I).

Flow cytometry gated on EpCAM^+^CD24^+^SSC-A^high^ cells (PCs) showed that cells from *Cdc42*^ΔIEC^ mice have significantly higher CellROX activity than cells from the *Cdc42*^WT^ littermates (Fig. 4J,K). These data revealed CDC42 as a potential regulator of the balance of oxidative stress and antimicrobial peptide production in the gut epithelium.

**Figure EV4:**
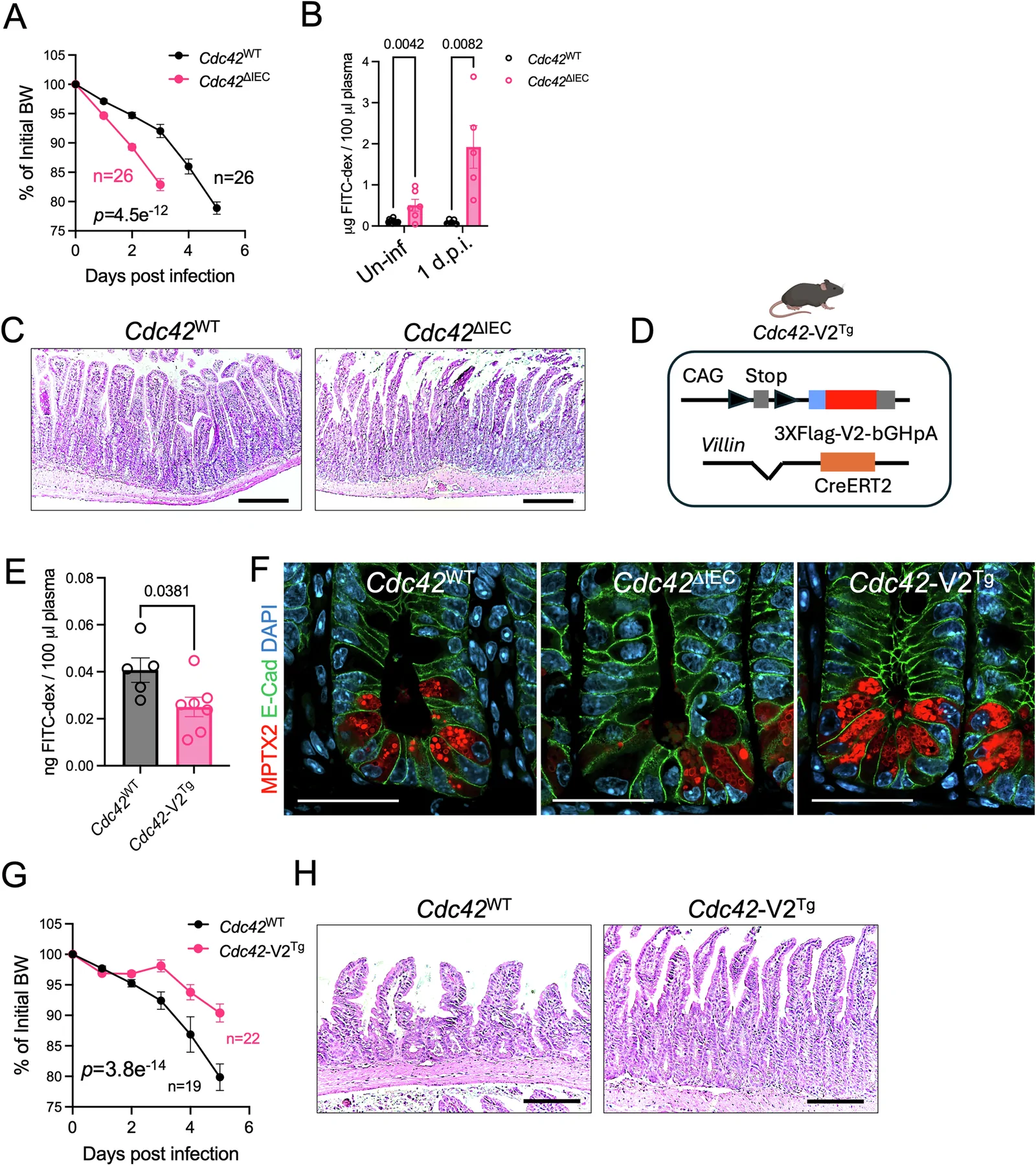
Cdc42 deficiency increases susceptibility to *S. Typhimurium* infection. (**A**) Body weight changes of *Cdc42*^*WT*^ and *Cdc42*^*ΔIEC*^ littermates following the *S. Typhimurium* oral inoculation. *n* = 26 for each condition. Curves were modeled using exponential (Malthusian) growth regression. Curves were compared using the Extra sum-of-squares F test. Data are presented as mean ± SEM. (**B**) An in vivo gut permeability assay was performed before the mice were sacrificed. Two hours after oral dosing of FITC-conjugated 4 kDa dextran, nanograms of dextran circulating in the plasma of uninfected mice and mice infected for one day were detected, for *Cdc42*^*WT*^ and *Cdc42*^*ΔIEC*^ mice. Statistical comparisons were performed using multiple unpaired *t*-tests with the two-stage step-up method of Benjamini, Krieger, and Yekutieli. Exact *p*-values are provided above the comparison bars. Data are presented as mean ± SEM. (**C**) Histology of infected *Cdc42*^WT^ and *Cdc42*^ΔIEC^ mouse ileum at 1 d.p.i. Scale bar, 100 µm. (**D**) Schematic diagram showing that *Cdc42*-V2^Tg^; Villin-CreER^T2^ mice overexpress the CDC42 isoform. (**E**) Mice were orally gavaged with FITC-conjugated 4 kDa dextran 2 h before sacrifice. Nanograms of fluorescent dextran were measured in plasma of *Cdc42*^WT^ (*n* = 5) and *Cdc42-*V2^Tg^ (*n* = 7) mice. A Student’s *t*-test was used to compare the difference. Exact *p*-value is provided above the comparison bar. Data are presented as mean ± SEM. (**F**) Immunofluorescent staining was performed for E-Cad and MPTX2 in *Cdc42*^WT^, *Cdc42*^ΔIEC^, and *Cdc42-*V2^Tg^ mice. Scale bar, 50 µm. (**G**) Body weights were measured from infected *Cdc42-*V2^Tg^ (*n* = 22) and *Cdc42*^WT^ (*n* = 19) littermates. Curves were modeled using exponential (Malthusian) growth regression. Curves were compared using the Extra sum-of-squares F test. Data are presented as mean ± SEM. (**H**) Histology of infected *Cdc42*^WT^ and *Cdc42-*V2^Tg^ mouse ileum. Scale bar, 100 µm.

ROS and AMP are both integral components of the innate defense system against pathogen infection. To test the impact of infection on these mice, we challenged *Cdc42*^ΔIEC^ mice along with their WT littermates with *S. Typhimurium*. All mice received a single injection of tamoxifen (75 mg/kg in corn oil) 7 days prior to infection, followed by oral administration of streptomycin 1 day before infection (Fig. 4L). Upon oral inoculation of *S. Typhimurium* (10^8^ CFU/mouse), the loss of body weight in *Cdc42*^ΔIEC^ mice was greater than that of WT littermates (Fig. EV4A), and all *Cdc42*^ΔIEC^ mice succumbed to the infection within 3 days (Fig. 4M). With FITC-dextran analysis, *Cdc42*^ΔIEC^ mice exhibited increased gut permeability prior to infection and 1 d.p.i. (days post infection) compared to the WT littermates (Fig. EV4B). Some knockout survivors analyzed at 1 d.p.i. showed a pronounced epithelial disruption and inflammation (Fig. EV4C).

In addition, we tested if overexpressing *Cdc42* would be protective against infection. The *Villin*-CreER^T2^*; Cdc42*-V2^Tg^ (IEC-V2^Tg^) mice (Zhang et al, 2020) previously reported by us were engineered to carry an inducible CDC42-variant 2 (V2) isoform expressed specifically in IECs upon CreER induction by tamoxifen (Fig. EV4D). CDC42-V2 isoform carries a palmitoylation site at its C terminal motif to increase plasma membrane-trafficking (Zhang et al, 2020). IEC-V2^Tg^ mice have an improved gut barrier at baseline (Fig. EV4E) and their PCs are densely packed with MPTX2 in secretory granules indicating their mature states (Fig. EV4F). IEC-V2^Tg^ mice subjected to the same regime of infection by *S. Typhimurium* showed a protection reflected by reduced mortality within the experimental window of post-infection (Fig. 4N), less body weight loss (Fig. EV4G), and improved pathology (Fig. EV4H).

### Cdc42-deficiency impairs mitochondrial function and increases oxidative stress in PCs

**Figure 5:**
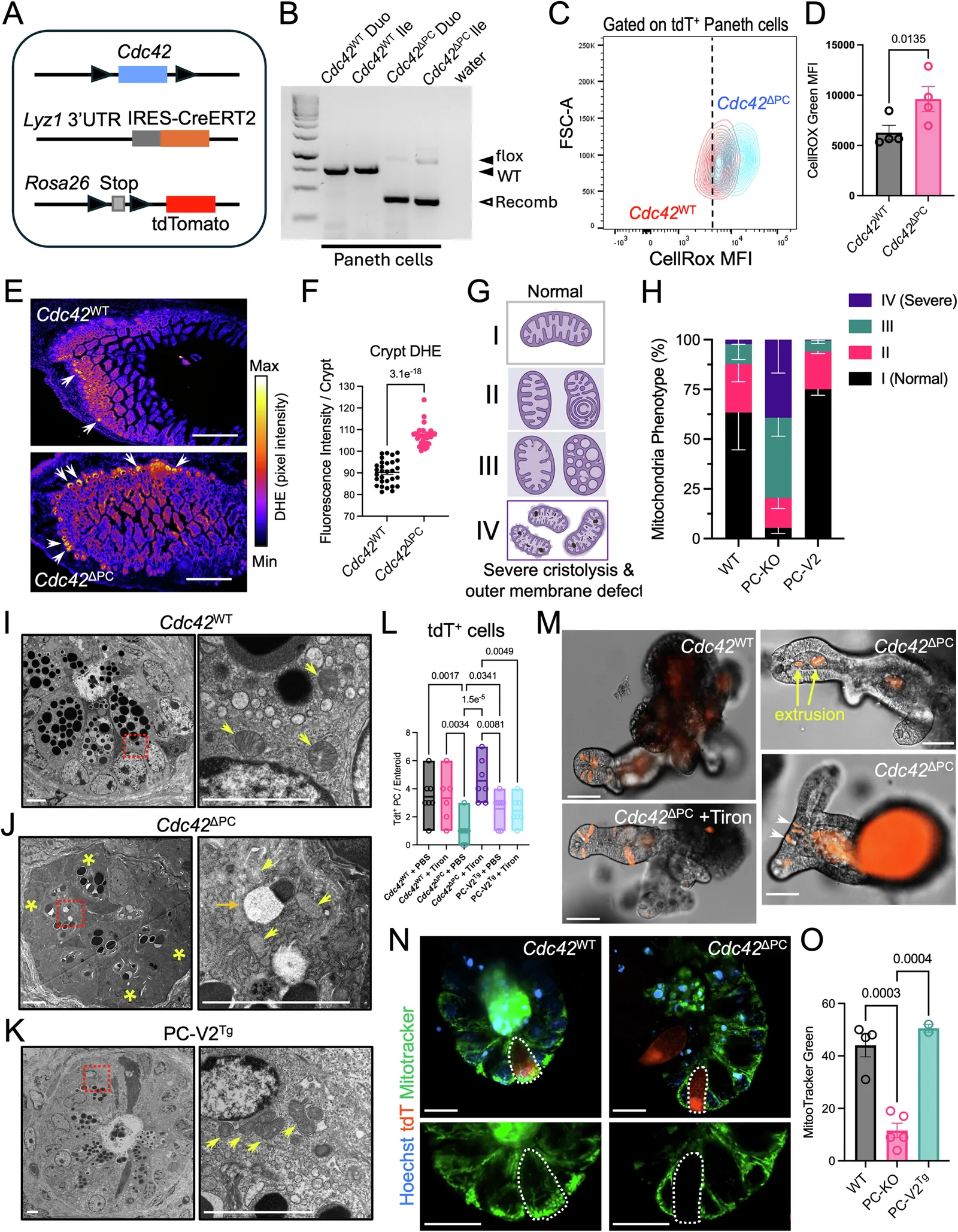
Ablation of Cdc42 in Paneth cells impairs mitochondrial function and elevates ROS. (**A**) Schematic diagram of *Cdc42*^ΔPC^ mice, in which the *Cdc42* is specifically deleted in Paneth cells by *Lyz1*^3’UTR-IRES-CreERT2^. (**B**) Genomic DNA PCR, using primers flanking the loxP sites, was performed to assess the efficacy of Cre-mediated recombination in flow-sorted tdT^+^ PCs. The arrow points to DNA fragments of the residual flox allele that was not recombined in duodenal and ileal PCs. (**C**) CellROX abundances were determined by flow cytometry in PCs from the ilea of *Cdc42*^WT^ and *Cdc42*^ΔPC^ mice. (**D**) A paired Student’s *t*-test was used to compare CellROX Green MFI. *n* = 4 mice per genotype. Data are presented as mean ± SEM. Exact *p*-value is provided above the comparison bar. (**E**) Cdc42^WT^ and Cdc42^ΔPC^ mouse ilea were stained for dihydroethidium (DHE). Fluorescent images were taken. White arrows indicate the crypts with high DHE intensity. Scale bar, 200 µm. (**F**) DHE intensities were quantified from randomly selected crypts of 3 mice per genotype and statistically analyzed using a Student’s *t*-test. Exact *p*-value is provided above the comparison bar. Data are presented as mean ± SEM. (**G**) Schematic diagrams detailing four categories of mitochondrial impairment based on the degree of cristolysis, and inner/outer membrane defects. I: normal (< 25% damage to cristae); II: loss of 25–50% of cristae; III: loss of 50–75% of cristae; IV: severe cristolysis, affecting >75% of cristae, in addition to inner or outer membrane defects. (**H**) TEM was performed on three *Cdc42*^WT^, three *Cdc42*^ΔPC^ (PC-KO), and two PC-V2^Tg^ mouse ileal samples. A total of 226 *Cdc42*^WT^, 302 *Cdc42*^ΔPC^, and 209 PC-V2^Tg^ mitochondria in Paneth cells were phenotyped according to panel G. Stacked bars represent the relative proportions within each genotype, normalized to 100%, with data shown as mean ± SEM. (**I**–**K**) Representative TEM images were taken of PCs of *Cdc42*^WT^, *Cdc42*^ΔPC^, and PC-V2^Tg^ mice. Yellow arrows indicate mitochondria. In the left panel of (**J**), yellow asterisks mark Paneth cells. Scale bar, 4 µm. (**L**,** M**) Enteroids were cultured from *Cdc42*^WT^ and *Cdc42*^ΔPC^ mouse duodenum. On day 3, 4-OHT was added to induce *Cdc42* deletion and tdT expression in PCs. Long yellow arrows show the extrusion of some PCs into the lumen, while short white arrows point to the up-migrating PCs in *Cdc42*^ΔPC^ enteroids. Wild-type or *Cdc42*^ΔPC^ enteroids treated with Tiron did not show extrusion of PCs. Scale bar, 50 µm. In (**L**), an ordinary one-way ANOVA followed by Fisher’s LSD test was performed to compare the total tdT^+^ cell number per enteroid in different conditions. Exact *p*-values are provided above the comparison bars. Data were obtained from three independent experiments and are presented as floating bars (min to max) with the central line indicating the mean. (**N**,** O**) Enteroids were cultured from *Cdc42*^WT^ and *Cdc42*^ΔPC^ mouse duodenum. On day 3, 4-OHT was added to induce *Cdc42* deletion and tdT expression in PCs. MitoTracker Green was used to label mitochondria and images were taken of live enteroids. White dotted lines delineate tdT^+^ Paneth cells. Scale bar, 10 µm. In (**O**), mitochondrial abundances per Paneth cell from *Cdc42*^WT^ (*n* = 4), *Cdc42*^ΔPC^ (*n* = 5), and PC-V2^Tg^ (*n* = 2) duodenal enteroids were analyzed. Statistical comparisons between groups were performed using an ordinary one-way ANOVA followed by Fisher’s LSD test. Exact *p*-values are provided above the comparison bars. Data are presented as mean ± SEM. Source data are available online for this figure.

To investigate CDC42 specifically in PCs, we generated PC-specific *Cdc42* knockout mice (*Cdc42*^ΔPC^) and mice with PC-specific expression of CDC42-V2 (PC-V2^Tg^) using the *Lyz1*^3’UTR-CreER^ driver (Balasubramanian et al, 2023) (Fig. 5A). Genomic DNA PCR from sorted tdT^+^ PCs from *Cdc42*^ΔPC^ mice showed an efficient recombination at the *Cdc42* locus in both the duodenum and ileum, with less than 5% of the floxed DNA alleles remaining un-recombined (Fig. 5B). Flow analysis revealed an elevated CellROX in *Cdc42*-deficient PCs (Fig. 5C,D). Dihydroethidium (DHE) staining, which probes for superoxide and H_2_O_2_, revealed a significantly elevated oxidative stress in the crypts of *Cdc42*^ΔPC^ intestines (Fig. 5E,F).

**Figure EV5:**
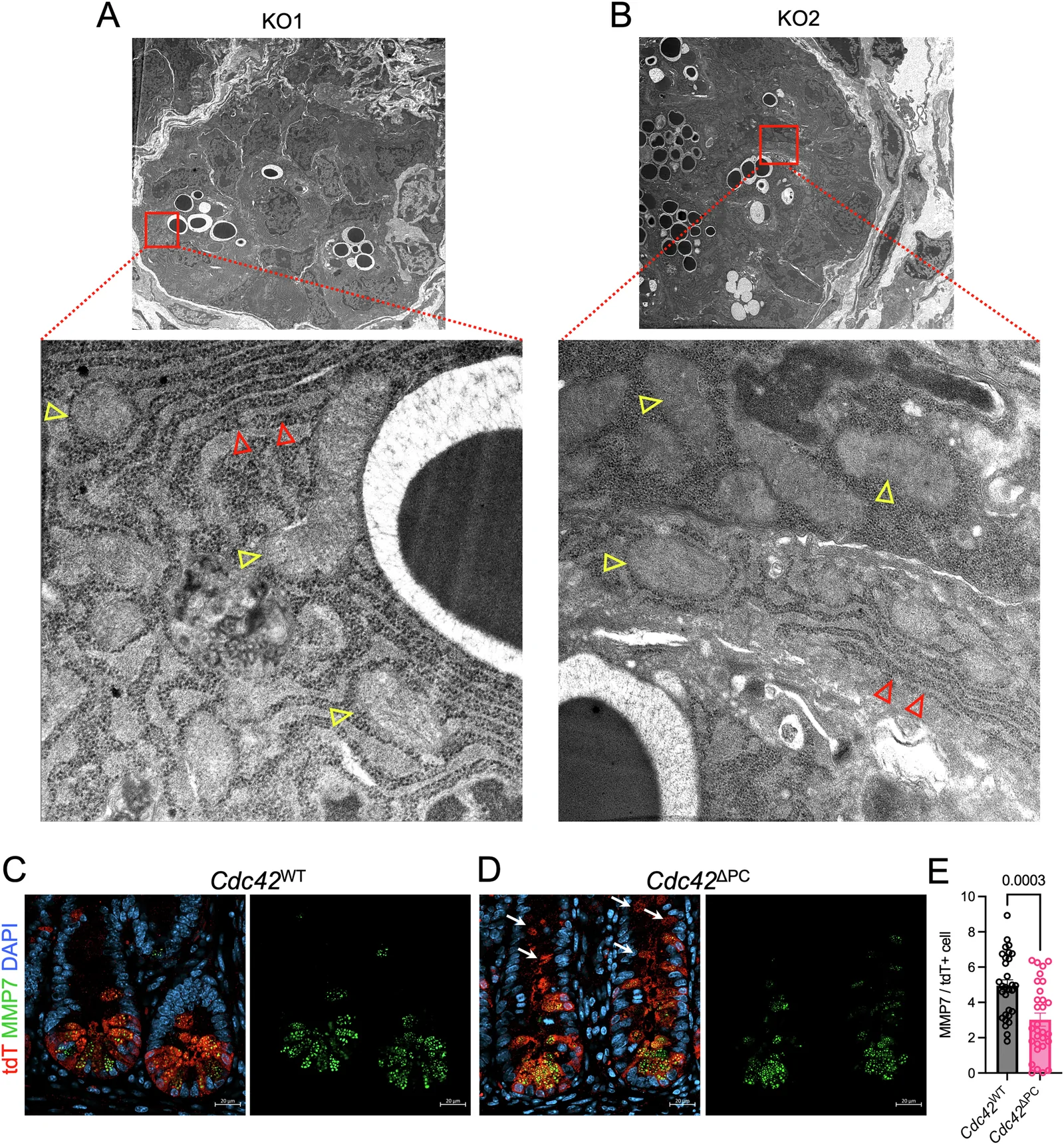
PC-specific deletion of *Cdc42* impairs mitochondrial integrity and MMP7 production. (**A**,** B**) TEM micrographs of *Cdc42*^ΔPC^ mouse ilea. These representative images illustrate the severe mitochondrial cristolysis (yellow arrows) and pronounced ER lumen dilation (red arrows) in virtually all the PCs of *Cdc42*^ΔPC^ mice. (**C**,** D**) Confocal immunofluorescence analysis of MMP7 and tdT in *S. Typhimurium* infected *Cdc42*^WT^ and *Cdc42*^ΔPC^ mice. Arrows in panel F point to up-migrating tdT^+^ cells that do not express MMP7. (**E**) MMP7 abundances in individual tdT^+^ cells were quantified from *Cdc42*^WT^ and *Cdc42*^ΔPC^ mice, and statistically compared by a Student’s *t*-test. Exact *p*-value is provided above the comparison bar. Data are presented as mean ± SEM.

Transmission electron microscopic (TEM) analysis revealed a variety of subcellular defects in PCs of *Cdc42*^ΔPC^ mice, with the loss of integrity in the inner and outer mitochondrial membranes being the most noticeable changes (yellow arrows, Fig. 5G–J, Fig. EV5A,B). Approximately 80% mitochondria in *Cdc42*-deficient PCs showed moderate to severe loss of crista structures (arrowheads, Fig. 5J). Virtually all *Cdc42*-deficient PCs showed ER swelling (e.g., with a dilated lumen), which is a prominent signature of the ER stress response (red arrows, Fig. EV5A,B). In addition, *Cdc42*-deficient PCs contained morphologically aberrant secretory granules with the electron dense core shifted to the side of the vesicles (arrow in Fig. 5J). *Cdc42*^WT^ and PC-V2^Tg^ mouse PCs contained 68% and 74% healthy mitochondria, respectively (Fig. 5G–I,K).

We then grew the enteroids from *Cdc42*^ΔPC^ and *Cdc42*^WT^ mice and monitored tdT^+^ cells following the administration of 4-OHT. Interestingly, PCs in *Cdc42*^ΔPC^ enteroids exhibited two different behaviors: some were extruded into the lumen (yellow arrows, Fig. 5M) while others migrated up to the villus compartment (white arrows, Fig. 5M), which likely led to a significant reduction in the number of PCs per *Cdc42*^ΔPC^ enteroid compared to *Cdc42*^WT^ enteroid (Fig. 5L). Treating *Cdc42*^ΔPC^ enteroids with Tiron significantly elevated (e.g., restored) PC number (Fig. 5L), suggesting that reducing oxidative stress could reduce PC extrusion. Live imaging of enteroids loaded with MitoTracker green showed reduced fluorescent signals indicating an impaired mitochondrial integrity in *Cdc42*-deficient PCs (Fig. 5N,O).

### Cdc42-deficient PCs have elevated plasticity

**Figure 6:**
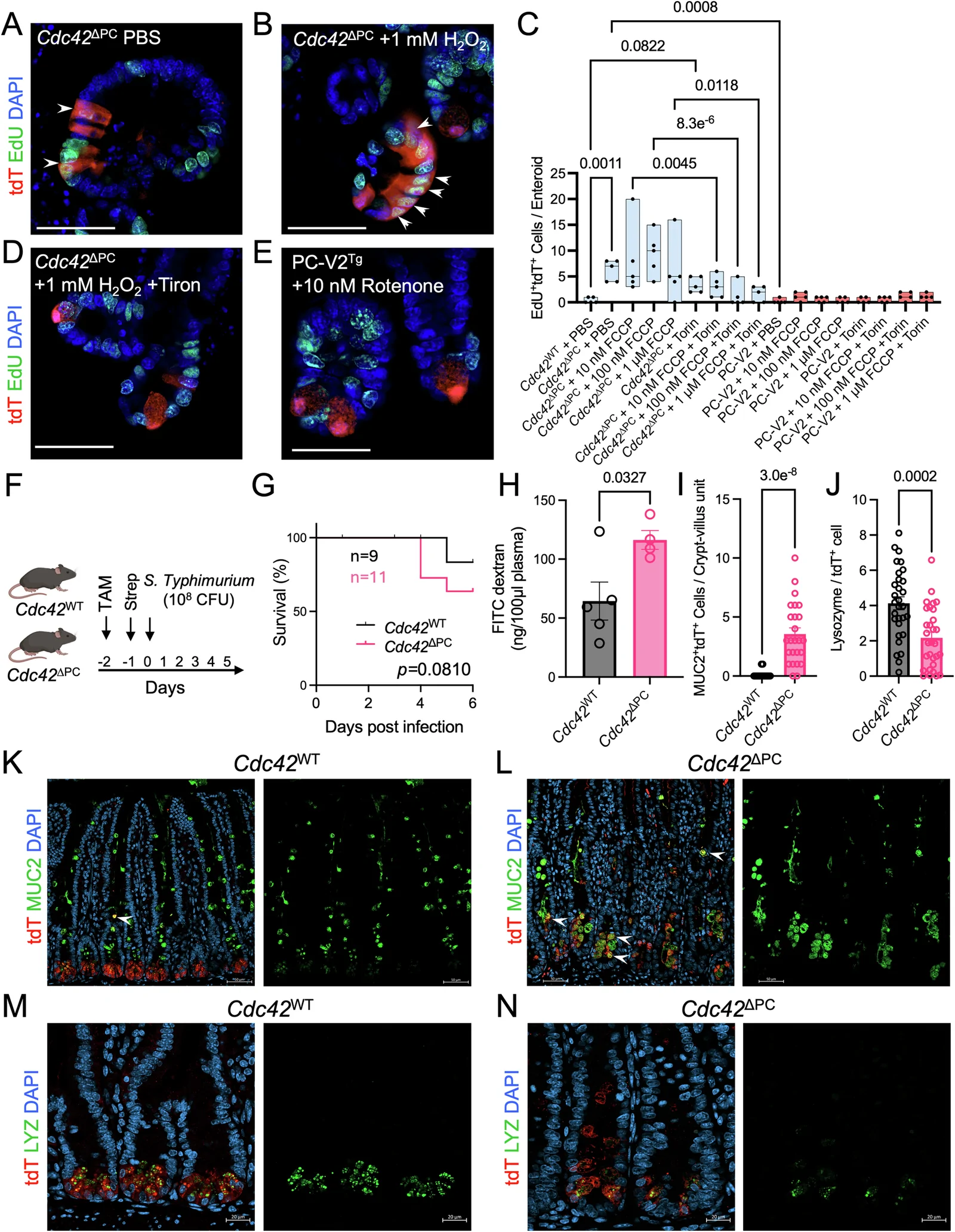
Cdc42-deficient PCs show plasticity ex vivo and in vivo. (**A**,** B**) Enteroids were grown from *Cdc42*^ΔPC^ mouse duodenum and induced with 4-OHT for *Cdc42* deletion and tdT expression. Enteroids were treated with PBS or H_2_O_2_ and labeled with EdU for 1 h before fixation and staining. Representative images show the detection of EdU^+^tdT^+^ cells (arrowheads) in both PBS-treated and H_2_O_2_-treated enteroids. Scale bar, 20 µm. (**C**) EdU^+^tdT^+^ cells in individual enteroids across different treatments were recorded. An ordinary one-way ANOVA followed by Fisher’s LSD test was performed to compare the statistical difference. Exact *p*-values are provided above the comparison bars. (**D**) Similar experiments to the above were performed for *Cdc42*^ΔPC^ enteroids treated with Tiron. (**E**) Similar experiments to the above were performed for PC-V2^Tg^ enteroids treated with rotenone. Scale bar, 50 µm. Experiments were repeated twice. (**F**) Schematic diagram outlining the *S. Typhimurium* infection experimental time-course for *Cdc42*^WT^ and *Cdc42*^ΔPC^ littermates. (**G**) Survival analysis and a one-tailed Gehan-Breslow-Wilcoxon test were performed for infected *Cdc42*^WT^ (*n* = 9) and *Cdc42*^ΔPC^ (*n* = 11) mice. (**H**) Two hours after oral dosing of FITC-conjugated 4 kDa dextran, nanograms of dextran circulating in the plasma of infected *Cdc42*^WT^ (*n* = 5) and *Cdc42*^ΔPC^ (*n* = 4) mice were measured. Comparison was made using a Student’s *t*-test. Exact *p*-value is provided above the comparison bar. Data are presented as mean ± SEM. (**I**) Quantification of the numbers of MUC2^+^tdT^+^ cells from 25 crypt-villus units in the ilea of infected *Cdc42*^WT^ (*n* = 2) and *Cdc42*^ΔPC^ (*n* = 2) mice, based on confocal fluorescence analysis (**K**, **L**). Comparison was made by a Student’s *t*-test. Exact *p*-value is provided above the comparison bar. Data are presented as mean ± SEM. (**J**) Quantification of the lysozyme abundance in 30 individual tdT^+^ cells from the ilea of infected *Cdc42*^WT^ (*n* = 2) and *Cdc42*^ΔPC^ (*n* = 2) mice, based on confocal fluorescence analysis (**M**,** N**). Comparison was made by a Student’s *t*-test. Exact *p*-value is provided above the comparison bar. Data are presented as mean ± SEM. (**K**–**N**) Confocal fluorescence analysis was performed for tdT, MUC2 or LYZ in *Cdc42*^WT^ and *Cdc42*^ΔPC^ mouse ilea. Arrows in (**K**, **L**) point to MUC2^+^tdT^+^ cells. Source data are available online for this figure.

An EdU incorporation assay for 1 h showed that PCs in *Cdc42*^ΔPC^ enteroids had an elevated number of EdU^+^tdT^+^ cells at the baseline (Fig. 6A,C). The number of EdU^+^tdT^+^ cells was further increased by H_2_O_2_, Rotenone, or FCCP treatment in *Cdc42*^ΔPC^ enteroids (Fig. 6A,B). These increases could be blocked by Tiron (Fig. 6A,C,D). In contrast, PCs in PC-V2^Tg^ mouse enteroids, where CDC42 is specifically overexpressed in Paneth cells, had a diminished response to ROS treatments (Fig. 6C,E), suggesting that loss of CDC42 elevates PC cell-cycle entry likely through ROS activity.

We then examined in vivo whether *Cdc42*-deficient PCs exhibit elevated plasticity upon challenges. All mice were administered tamoxifen, pretreated with streptomycin 24 h later, and orally inoculated with 10^8^ CFU *S. Typhimurium* on the subsequent day (Fig. 6F). The mortality of *Cdc42*^ΔPC^ mice upon infection was moderately increased compared to that of *Cdc42*^WT^ littermates (Fig. 6G) with elevated gut permeability at 4 d.p.i. (Fig. 6H). Lineage tracing of *Cdc42*-deficient PCs in the infected mice showed that a significantly increased number of tdT^+^ cells gave rise to MUC2^+^ goblet cells (Fig. 6I,K,L); whereas there was a greater number of tdT^+^ cells that did not express lysozyme (Fig. 6J,M,N) or MMP7 (Fig. EV5C–E) anymore, supporting an increased plasticity of *Cdc42*-deficient PCs.

**Figure 7:**
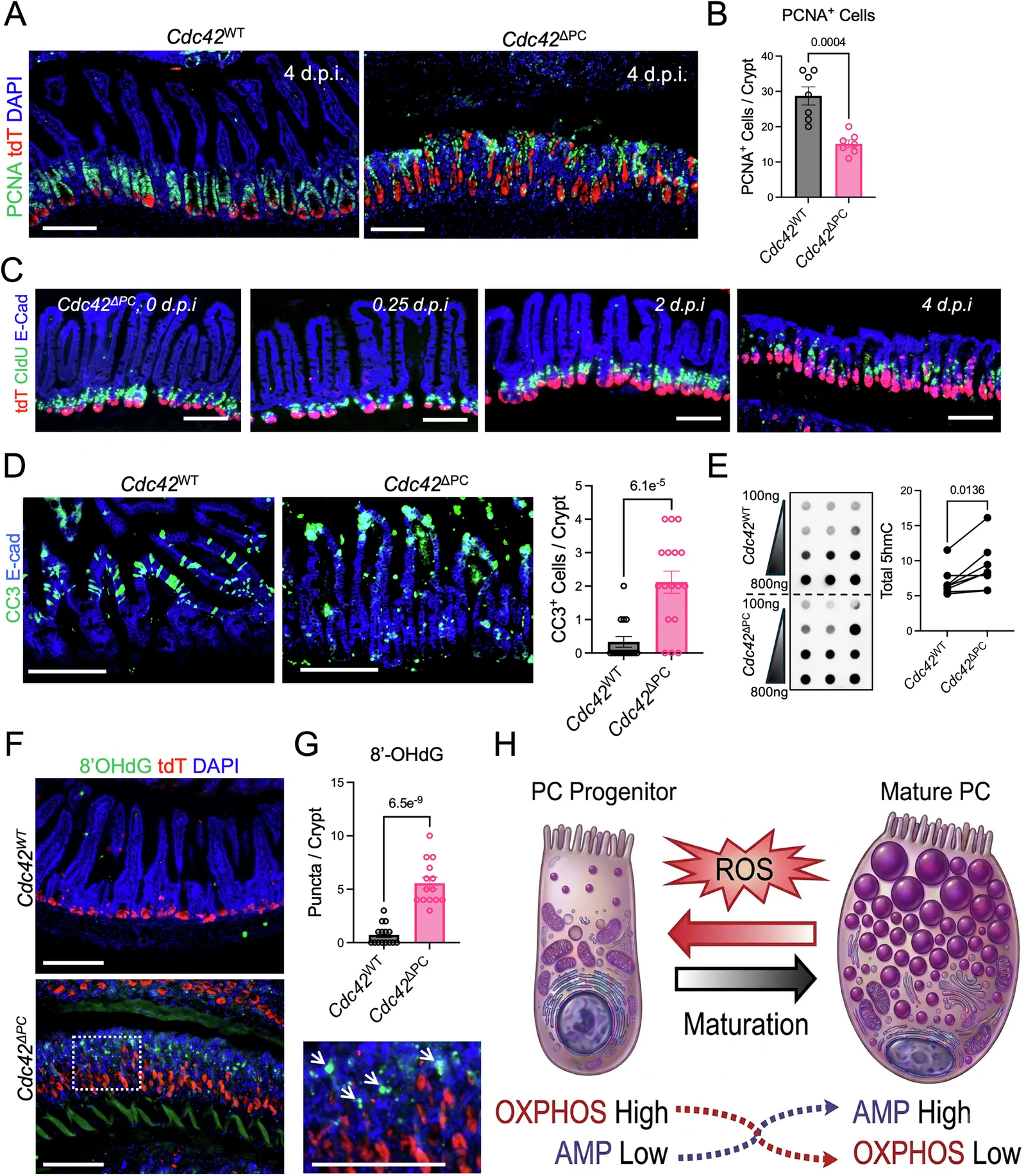
PC-specific Cdc42 knockout mice show elevated oxidative stress during infection. (**A**,** B**) Representative immunofluorescence images for tdT, PCNA, and DAPI of *Cdc42*^*WT*^ and *Cdc42*^*ΔPC*^ mouse ilea 4 d.p.i. PCNA^+^ cell numbers in individual crypts were determined and statistically compared by a Student’s *t*-test. Exact *p*-value is provided above the comparison bar. Scale bar, 100 µm. Data are presented as mean ± SEM. (**C**) *Cdc42*^*ΔPC*^ mice were infected with *S. Typhimurium* and sacrificed after 6 h, 2 days, and 4 days. All mice were injected with 5-chloro-2′-deoxyuridine (CldU) 1 h prior to sacrifice. Representative immunofluorescence images of tdT (red), CldU (green), and E-Cad (blue). Scale bar, 100 µm. (**D**) Immunofluorescence staining was performed for cleaved caspase 3 (CC3) in infected *Cdc42*^*WT*^ and *Cdc42*^*ΔPC*^ mouse ilea and compared using a Student’s *t*-test. Exact *p*-value is provided above the comparison bar. Data are presented as mean ± SEM. Scale bar, 100 µm. (**E**) Dot blotting analysis for 5hmC was performed using IECs’ DNA extracted from WT and Cdc42-KO littermates and quantified by a paired Student’s *t*-test. Exact *p*-value is provided above the comparison bar. n = 8 per genotype. (**F**,** G**) Immunofluorescence staining was performed for 8-hydroxy-2’ -deoxyguanosine (8-OHdG) in infected *Cdc42*^*WT*^ and *Cdc42*^*ΔPC*^ mouse ilea (*n* = 3 mice) and the 8-OHdG puncta (arrows) were determined in individual crypts. Scale bars, 100 µm. In (**G**), the difference was compared using a Student’s *t*-test. Exact *p*-value is provided above the comparison bar. Data are presented as mean ± SEM. (**H**) Schematic diagram showing a physiological decline in OXPHOS during the maturation of Paneth cells. This developmental program may be reverted by a pathological oxidative stress, caused by extracellular ROS production (such as during pathogen infection and inflammation) or by impairment in mitochondrial oxidative respiration. This pathological oxidative stress diminishes the antimicrobial function of PCs. Source data are available online for this figure.

**Figure EV6:**
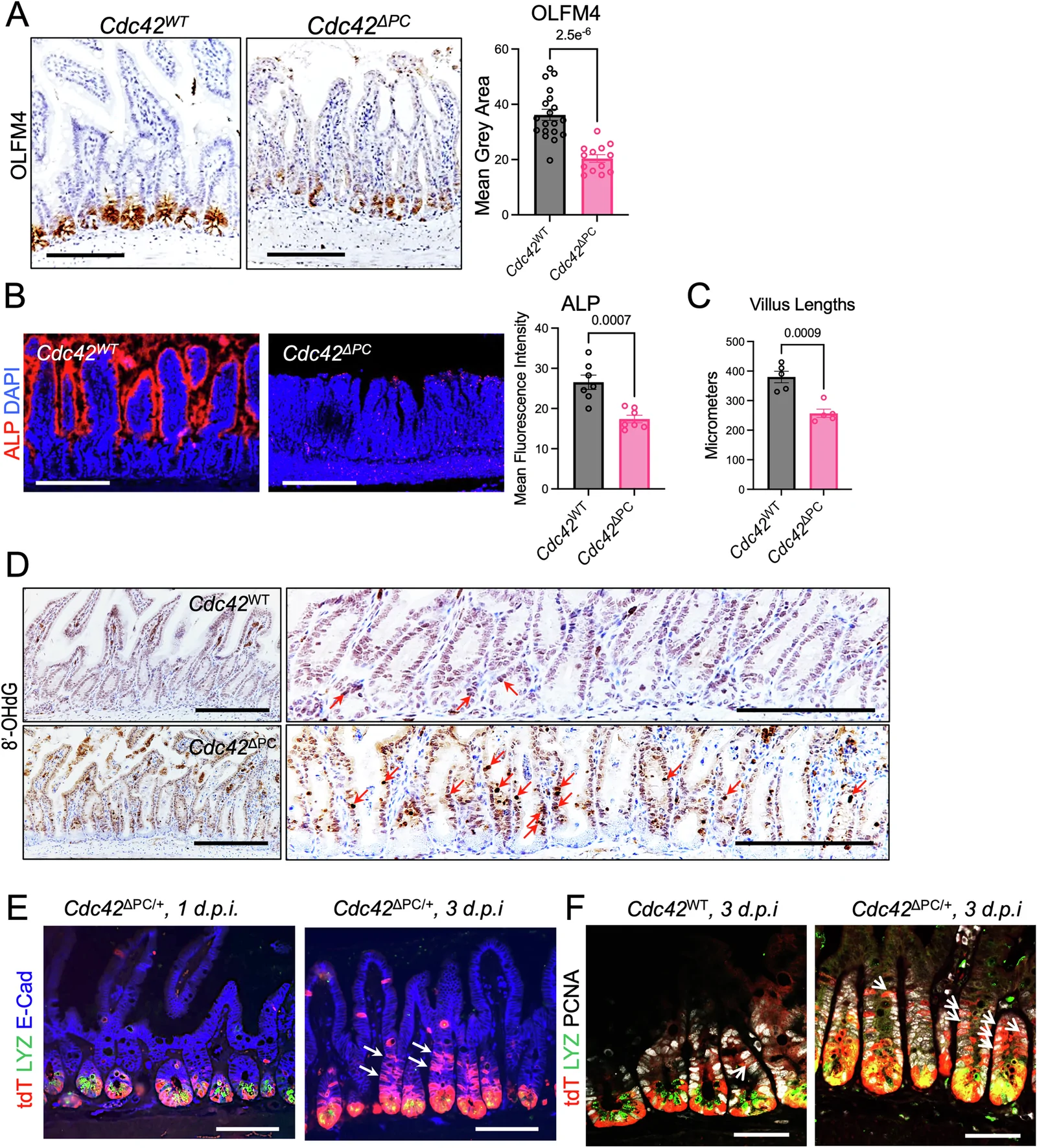
PC-specific *Cdc42* knockout reduces stem cell and enterocyte populations and elevates DNA oxidation during *S. Typhimurium* infection. (**A**) Immunostaining was performed for OLFM4 in infected *Cdc42*^WT^ and *Cdc42*^ΔPC^ mouse intestines. A Student’s *t*-test was performed to compare OLFM4 abundance in crypts between the two groups. Exact *p*-value is provided above the comparison bar. Scale bar, 100 µm. Data are presented as mean ± SEM. (**B**) Immunostaining was performed for alkaline phosphatase (ALP) in infected *Cdc42*^WT^ and *Cdc42*^ΔPC^ intestines. Scale bar, 100 µm. A Student’s *t*-test was performed to compare the ALP between the two groups. Exact *p*-value is provided above the comparison bar. Data are presented as mean ± SEM. (**C**) Villus lengths were measured from the infected *Cdc42*^WT^ and *Cdc42*^ΔPC^ mice at 4 d.p.i. and analyzed by a Student’s *t*-test. Exact *p*-value is provided above the comparison bar. Data are presented as mean ± SEM. (**D**) Immunostaining for 8’-OHdG using infected *Cdc42*^WT^ and *Cdc42*^ΔPC^ intestinal sections. Red arrows point to 8’-OHdG puncta. Scale bar, 100 µm. (**E**) Tamoxifen was injected into *Cdc42*^ΔPC/+^ mice carrying a PC reporter. Mice were infected with *S. Typhimurium and* sacrificed at 1 and 3 d.p.i. Sections were stained for tdT and lysozyme. Arrows point to up-migrating tdT^+^ cells. Scale bar, 50 µm. (**F**) Tamoxifen was injected into *Cdc42*^WT^ and *Cdc42*^ΔPC/+^ carrying a PC reporter. Mice were infected with *S. Typhimurium and* sacrificed at 3 d.p.i. Sections were stained for tdT, lysozyme, and PCNA. Arrows point to PCNA^+^tdT^+^ cells. Scale bar, 50 µm.

Severe oxidative stress is known to cause collateral tissue damage such as DNA oxidation (Barzilai and Yamamoto, 2004) that is detrimental to proliferative cells. PCNA staining suggested that infected *Cdc42*^ΔPC^ intestines at 4 d.p.i. have a disrupted transit-amplifying cell compartment (Fig. 7A,B) and a reduced OLFM4^+^ cell population in the crypts (Fig. EV6A). Some severely ill *Cdc42*^ΔPC^ mice exhibited a total loss of enterocytes (Fig. EV6B) and villus blunting (Fig. EV6C). We examined the kinetics of PCs’ response to infection in *Cdc42*^ΔPC^ mice by sacrificing the animals at 0, 6-h, 2 and 4 d.p.i., and we observed a gradual loss of dividing cells (CIdU^+^) in these mice, with tdT^+^ cells remaining in the damaged epithelial layer (4 d.p.i., Fig. 7C). Crypt epithelial cell death was significantly elevated in *Cdc42*^ΔPC^ mice (Fig. 7D).

Biochemical and immunohistochemistry analysis for DNA oxidation markers: 5-hydroxymethylcytosine (Niu et al, 2015) (Fig. 7E) and 8-hydroxydeoxyguanosine (8’-OHdG) (Bhattacharyya et al, 2014) (Figs. 7F,G and EV6D), revealed significant accumulation of DNA oxidation in these infected *Cdc42*^ΔPC^ intestines. These 8’-OHdG positive cells identified by 8’-OHdG puncta (white arrows, Fig. 7F) are located near the *Cdc42*-deficient tdT^+^ cells, suggesting that there was an increased oxidative stress level that might be caused by the PC defects, contributing to an overall elevated oxidative damage across the tissue compartment. Because *Cdc42* plays diverse roles in multiple pathways crucial for cell survival, we next tested whether maintaining a single allele of *Cdc42* could preserve cellular survivability while allowing an enhanced lineage tracing. *Cdc42*^ΔPC/+^ mice were infected with 10^8^ CFU *S. Typhimurium* via oral gavage 2 days after tamoxifen injection. At 3 d.p.i, a notable population of tdT^+^ cells migrated into villus compartments (Fig. EV6E). Some of these up-migrating *Cdc42*^ΔPC/+^ tdT^+^ cells are positive for PCNA supporting their transit-amplifying states (Fig. EV6F).

## Discussion

To our knowledge, the Paneth cell lineage-tracing experiments reported here provide the first evidence that excessive oxidative stress, often produced in tissues under pathological conditions such as infection and inflammation, can directly stimulate a subset of Paneth cells to proliferate and dedifferentiate. The observed ROS-induced proliferative progenies from Paneth cells have reduced secretory granules but take upon a tall-columnar morphology reminiscent of the transit-amplifying (TA) cell types. The expansion of these ROS-activated progenies take place within 1–2 days of stimulation. A small subset of these activated progenies then dedifferentiates, transit amplifies, and re-differentiates into other IEC cell types, suggesting a feature of self-renewal activated by oxidative stresses. We and others have reported Paneth cell plasticity in mice in response to whole body irradiation or pro-inflammatory chemicals (Jones et al, 2018; Schmitt et al, 2018; Yu et al, 2018). All these challenges incur massive oxidative stresses in the gut tissues. Inflammatory immune cells such as the neutrophils can be a major source of the extra-epithelial cell ROS. As dividing cells are more sensitive to oxidative stress, it is plausible that the loss of proliferative stem or TA cells under severe pathological oxidative stress stimulate some of the ROS-tolerating Paneth cells to contribute to epithelial renewal while dampening their canonical AMP functions.

Mouse GPX2 is enriched in the crypt cells (Florian et al, 2010; Komatsu et al, 2001), and in human intestine, these glutathione peroxidase are also enriched in Paneth cells (Florian et al, 2001). Loss of *Gpx1* and *Gpx2* in mice caused ileocolitis (Chu et al, 2004), which could, in part, attribute to an impaired antioxidant capacity in Paneth cells. In comparison, loss of the epithelial Superoxide dismutase 1, another antioxidant enzyme in mice induced apoptosis in the stem cell regions though no reactive oxygen species change was observed in the organoids (Wang et al, 2022). A decline in the amount of lysozyme granule was observed in *Cat*^-/-^ or *Nnt*^-/-^ mice that lack the antioxidant enzymes catalase and nicotinamide nucleotide transhydrogenase, though no reduction of Paneth cell number was observed (Ramos-Leon et al, 2024). These observations agree with suppression of antimicrobial function by oxidative stress. Our Paneth cell-specific scRNA transcriptome reveals that the progenitor Paneth cells have enriched mitochondrial oxidative phosphorylation and reactive oxygen species gene sets, suggesting that the newly born Paneth cells likely inherit these transcriptional programs from the ISCs. During the transition of Paneth cell progenitors to mature Paneth cells, these transcriptomes are dampened, suggesting that a dampened oxidative signaling may be required for Paneth cells to fully mature into antimicrobial and secretory phenotype. This inverse correlation of ROS and antimicrobial function was speculated (Simmons et al, 2016), and our study here provided direct evidence that oxidative stresses diminish antimicrobial secretory features in Paneth cells (Fig. 7H).

Approximately half of Paneth cell population carries high OXPHOS transcriptomic signature based on our transcriptome and flow cytometry analysis. These intrinsic differences among Paneth cell populations appear independent of gut microbiota, as Paneth cells of GF mice maintained a similar difference. However, Paneth cells of SPF mice have a higher mitochondrial activity than CD24-negative non-Paneth cells (e.g., enterocytes), and this difference is lost in GF mice, suggesting that microbiota can modify mitochondrial and oxidative respiration programs in Paneth cells.

Previous studies showed that a physiological mitochondrial respiration is required for ISCs to differentiate (Rodriguez-Colman et al, 2017) and ISC-specific deletion of the mitochondrial chaperon Hsp60 led to aberrant Paneth cell phenotype (Khaloian et al, 2020). Our study expands on the concept by showing Paneth cell dedifferentiation into mitotic progenitors upon uncoupling the mitochondrial oxidative respiration. Thus, the physiological and pathological reactive oxygen species signaling plays a role in epithelial differentiation and dedifferentiation shown in this study. Interestingly, mice lacking Prohibitin 1, an inner mitochondrial membrane protein for respiratory chain assembly showed antimicrobial peptide granule defects in Paneth cells prior to spontaneous inflammation (Jackson et al, 2020). It was unknown if Prohibitin 1 knockout Paneth cells behave in a similar fashion as shown by *Cdc42*-knockout Paneth cells. It is possible that any genetic or environmental factors disrupting the redox management in Paneth cells could affect their plasticity and granulation phenotype.

We and others have reported that IEC-specific ablation of *Cdc42* affects ISC function (Sakamori et al, 2012), Tuft cell development (Long et al, 2022; Zhang et al, 2020), colonic tumorigenesis (Sakamori et al, 2014), MHCII expression in villus IECs (Tuganbaev et al, 2020), and transcytosis of commensal bacterial antigens to regulate T-cell function (Ladinsky et al, 2019). However, the finding of CDC42-regulated oxidative stress and antimicrobial responses in Paneth cells using the *Cdc42*^ΔPC^ mouse model is novel. Similar to the observed detrimental outcome of infected *Cdc42*^ΔPC^ mice in our study, loss of Cdc42 in neutrophil also resulted in fetal airway infection (Lee et al, 2013), collectively reflecting a potentially unifying innate defense mechanism by CDC42. At the molecular level, a loss of *Cdc42* in Paneth cells may increase the production of reactive oxygen species, as it was reported in an earlier study that CDC42 inhibits the production of superoxide upon leukocyte stimulation via a NOX-dependent mechanism (Diebold et al, 2004). There, an antagonism between RAC and CDC42 modulate ROS generation. In another study, CDC42 was shown to inhibit neutrophil extracellular trap via the mitochondria (Tackenberg et al, 2021). Abnormal reactive oxygen species accumulation was also reported in *Cdc42*-deficient dental epithelium through mitochondrial disruption (Zheng et al, 2024). The regulation of reactive oxygen species production by CDC42 may be conserved even in unicellular fungal cells (Kowalewski et al, 2021).

Upon induced *Cdc42* ablation in *Cdc42*^ΔPC^ enteroids, we observed two distinct behaviors in the knockout Paneth cells: rapid extrusion into the lumen or cell-cycle entrance with migrating into the villus compartment. Both behaviors were suppressed by ROS scavenger indicating that they are likely caused by oxidative stress. The extrusion phenotype is reminiscent of IFN-γ (Farin et al, 2014), which was reported to indeed stimulate mitochondrial activity, ROS production, and Paneth cell secretion (Encarnacion-Garcia et al, 2024). De novo CDC42 mutations (p.Tyr64Cys; p.Cys81Tyr; p.Arg186Cys; p.Cys188Tyr; and p.*192C*24 causing a read-through and adding 24 amino acids to the C-terminus) are identified in patients of neonatal-onset severe auto-inflammation (Gernez et al, 2019), syndromic immunodeficiency (Szczawinska-Poplonyk et al, 2020), hepatosplenomegaly and hemophagocytic lymphohistiocytosis (Lam et al, 2019), systemic inflammation, Takenouchi-Kosaki syndrome, myelofibrosis (Bucciol et al, 2020; Motokawa et al, 2018). These CDC42 mutations affect its C-terminal, switch II domain, or the nucleotide-binding pocket. The p.Tyr64Cys mutation abolishes the entire GTPase activity while Arg186Cys causes aberrant subcellular localization, all of which could potentially affect its ROS-regulatory capacities. We observed a protection against lethal infection in mice overexpressing the CDC42-V2 isoform in the IECs that previously also showed a protection against irradiation injury to the gut (Zhang et al, 2020). Paneth cells overexpressing CDC42 seem to have reduced cellular oxidative stress, while lacking CDC42 robustly impair mitochondrial integrity and trigger ER stress, based on the electron microscopy study. Enhancing oxidative management in Paneth cells may offer a protection to overall gut health.

Several limitations of this work should be noted. First, *Lyz1* is also expressed in certain types of immune cells, including macrophages such as the alveolar macrophages (Arroyo Portilla et al, 2023; Garcia-Gonzalez et al, 2025). *Cdc42* deletion by *Lyz1*^3’UTR-IRES-CreER^ driver may also affect immune cells thereby the in vivo phenotypes of infected mice may attribute to non-PC cell defects, which remains to be elucidated in the future. However, the enteroid results in this study definitively proved the direct ROS effects on epithelial cells. Additionally, we found that PCs in ileal enteroids can often tolerate higher doses of ROS without giving rise to lineage-tracing events ex vivo. This resistance may reflect underlying differences in gene expression between ileal and duodenal PCs, as previously reported by our group and others (Balasubramanian et al, 2023; Timmermans et al, 2024).

## Methods

**Table:** Reagents and tools table

Reagent/resource	Reference or source	Identifier or catalog number
**Experimental models**		
*Cdc42*^flox^ mice	Wu et al, 2006	
*Cdc42*-V2^Tg^ mice	Our Lab	N/A (Zhang et al, 2020)
*Lyz1*^3’UTR-IRES-CreER^ mice	Our Lab	N/A (Balasubramanian et al, 2023)
B6.Cg-Tg(Vil1-cre/ERT2)23Syr/J	The Jackson Laboratory	Strain: 020282
B6.Cg-Gt(ROSA)26Sor^tm14(CAG-tdTomato)Hze^/J	The Jackson Laboratory	Strain: 007914
*Salmonella typhimurium* strain SL1344	Beth McCormick (University of Massachusetts Medical School)	N/A (Hoiseth et al, 1981)
**Antibodies**		
APC/Fire™ 750 anti-mouse CD24 Antibody (Clone: M1/69)	Biolegend	Cat#101840, RRID: AB_2650876
PE/Cyanine7 anti-mouse CD326 (Ep-CAM) Antibody (Clone: G8.8)	Biolegend	Cat#118216, RRID: AB_1236471
Purified Anti-Mouse CD16/CD32 (Fc Shield) (2.4G2)	TONBO® REAGENTS	Cat#70-0161-M001, RRID: AB_2621487
Mouse anti-GPX1/2	Santa Cruz Biotech	Cat#133160, RRID: AB_2112237
Villin Antibody (1D2C3)	Santa Cruz Biotech	Cat#sc-58897, RRID: AB_2304475
PCNA (PC10)	Santa Cruz Biotech	Cat#sc-56, RRID: AB_628110
Olfm4 (D6Y5A) Rabbit Monoclonal Antibody	Cell Signaling Technology	Cat#39141, RRID: AB_2650511
Cdc42 Antibody	Cell Signaling Technology	Cat#2462, RRID: AB_2078085
Rabbit Anti-MMP7 Monoclonal Antibody	Cell Signaling Technology	Cat#3801, RRID: AB_2144465
PCNA (PC10) Mouse mAb	Cell Signaling Technology	Cat#2586, RRID: AB_2160343
Cleaved Caspase-3 (Asp175) (5A1E) Rabbit Monoclonal Antibody	Cell Signaling Technology	Cat#9664, RRID: AB_2070042
Lysozyme Polyclonal Antibody	ThermoFisher Scientific	Cat#PA1-29680, RRID: AB_1955851
Nrf2 Polyclonal Antibody	ThermoFisher Scientific	Cat#PA5-27882, RRID: AB_2545358
Donkey anti-Rabbit IgG (H + L) Highly Cross-Adsorbed Secondary Antibody, Alexa Fluor™ 488	ThermoFisher Scientific	Cat#A-21206, RRID: AB_2535792
Donkey anti-Goat IgG (H + L) Cross-Adsorbed Secondary Antibody, Alexa Fluor™ 568	ThermoFisher Scientific	Cat#A-11057, RRID: AB_2534104
Donkey anti-Rabbit IgG (H + L) Highly Cross-Adsorbed Secondary Antibody, Alexa Fluor™ 647	ThermoFisher Scientific	Cat#A-31573, RRID: AB_2536183
Donkey anti-Mouse IgG (H + L) Highly Cross-Adsorbed Secondary Antibody, Alexa Fluor™ 647	ThermoFisher Scientific	Cat#A-31571, RRID: AB_162542
Anti-DCAMKL1 antibody [EPR6085]	Abcam	Cat#ab109029, RRID: AB_10864128
Anti-MUC2 antibody [EPR23479-47]	Abcam	Cat#ab272692, RRID: AB_2888616
Anti-MPTX + MPTX2 antibody [EPR20920-19]	Abcam	Cat#ab238123, RRID: AB_3714714
Mouse Anti-8-Hydroxy-2’-deoxyguanosine Monoclonal Antibody	Abcam	Cat#ab48508, RRID: AB_867461
SQSTM1 monoclonal antibody (M01), clone 2C11	Abnova	Cat#H00008878-M01, RRID: AB_437085
GAPDH antibody [GT239]	GeneTex	Cat#GTX627408, RRID: AB_11174761
Anti-lysozyme	BioGenex	Cat#AR024, RRID: AB_2890235
Rabbit Anti-RFP Polyclonal Antibody	Rockland	Cat#600-401-379, RRID: AB_2209751
Goat Anti-RFP Polyclonal Antibody	Rockland	Cat#200-101-379, RRID: AB_2744552
Mouse Anti-5-Hydroxymethylcytosine (5-hmC) Antibody	Active Motif	Cat#39999, RRID: AB_2566808
Rat Anti-Brdu (used for detecting Cldu in this study)	Novus Biologicals	Cat#NB500-169, RRID: AB_10002608
Sheep Anti-Mouse IgG, HRP Conjugated	Cytiva	Cat#NA931, RRID: AB_772210
Donkey Anti-Rabbit IgG, HRP Conjugated	Cytiva	Cat#NA934, RRID: AB_772206
Mouse anti-E-Cadherin (E-Cad)	BD Biosciences	Cat#610182; RRID: AB_397581
**Oligonucleotides and other sequence-based reagents**		
Fwd primer for mouse Cdc42: CTATACTTCATATACCACACG	IDT	N/A
Rev primer for mouse Cdc42: TCTGCCATCTACACATACAC	IDT	N/A
**Chemicals, enzymes, and other reagents**		
B-27™ Supplement (50X), serum free	ThermoFisher Scientific	Cat#17504044
N2 supplement (100x)	ThermoFisher Scientific	Cat#17502001
Advanced DMEM/F-12	ThermoFisher Scientific	Cat#12634010
HEPES (1 M)	ThermoFisher Scientific	Cat#15630080
GlutaMAX™ Supplement	ThermoFisher Scientific	Cat#35050061
Mouse Noggin Recombinant Protein	ThermoFisher Scientific	Cat#250-38-100UG
Mouse IFN-gamma Recombinant Protein	ThermoFisher Scientific	Cat#315-05-100UG
eBioscience™ Permeabilization Buffer (10X)	ThermoFisher Scientific	Cat#00-8333-56
CellROX™ Deep Red Reagent	ThermoFisher Scientific	Cat#C10422
CellROX™ Green Reagent	ThermoFisher Scientific	Cat#C10444
Click-iT™ Plus EdU Cell Proliferation Kit for Imaging, Alexa Fluor™ 488 dye	ThermoFisher Scientific	Cat#C10637
TrypLE™ Express Enzyme (1X), phenol red	ThermoFisher Scientific	Cat#12605028
MitoTracker™ Deep Red FM	ThermoFisher Scientific	Cat#M22426
MitoTracker Green	ThermoFisher Scientific	Cat#M46750
Prolong Gold Antifade Mountant	ThermoFisher Scientific	Cat#P36930
4’,6-Diamidino-2-Phenylindole (DAPI)	ThermoFisher Scientific	Cat#D1306
LIVE/DEAD™ Fixable Violet Dead Cell Stain Kit	ThermoFisher Scientific	Cat#L34964
Hoechst 33342 solution, 20 mM	ThermoFisher Scientific	Cat#62249
0.5 M EDTA	Fisher Scientific	Cat#BP24821
Hydrogen Peroxide (30% in Water)	Fisher Scientific	Cat#BP2633500
Triton™ X-100	Fisher Scientific	Cat#BP151-100
Methanol	Fisher Scientific	Cat#A412-4
PBS without Ca^2+^ and Mg^2+^	Corning	Cat#21-040-CM
Matrigel, Growth Factor Reduced	Corning	Cat#356231
Recombinant Mouse R-Spondin 1 Protein	R&D	Cat#3474-RS-250
Recombinant Mouse EGF Protein	R&D	Cat#2028-EG-200
Recombinant Mouse IL-17A Protein	R&D	Cat#421-ML-025/CF
Recombinant Mouse IL-22 Protein	R&D	Cat#582-ML-010/CF
FCCP	MedChemExpress	Cat#HY-100410
Rotenone	MedChemExpress	Cat#HY-B1756
Primocin	InvivoGen	Cat#ant-pm-1
4-Hydroxytamoxifen	Sigma-Aldrich	Cat#H6278
TWEEN® 20	Sigma-Aldrich	Cat#P1379
N-Acetyl-L-cysteine	Sigma-Aldrich	Cat#A9165
Bovine Serum Albumin	Sigma-Aldrich	Cat#A3294
Normal Donkey Serum	Sigma-Aldrich	Cat#D9663
Dihydroethidium	Sigma-Aldrich	Cat#37291
Acetonitrile, J.T.Baker®	VWR	Cat#BAKR9017-03
Amersham™ ECL Select™ Western Blotting Detection Reagent	Cytiva	Cat#RPN2235
RNase-Free DNase Set (50)	Qiagen	Cat#79254
Tissue-Tek® O.C.T. Compound	Electron Microscopy Sciences	Cat#62550-01
Sodium cacodylate buffer, 0.2 M, pH 7.4	Electron Microscopy Sciences	Cat#11650
8% Glutaraldehyde (EM grade)	Electron Microscopy Sciences	Cat#16019
16% Paraformaldehyde (EM grade)	Electron Microscopy Sciences	Cat#15700
4% Osmium Tetroxide (OsO_4_)	Electron Microscopy Sciences	Cat#19150
EMBed-812 kit	Electron Microscopy Sciences	Cat#14120
VECTASHIELD® PLUS Antifade Mounting Medium	Vector	Cat#H-1900-10
**Software**		
Graphpad Prism (v. 11.0.2)	Graphpad	https://www.graphpad.com/
FlowJo software (v. 10.9.0)	FlowJo LLC	https://www.flowjo.com/flowjo10/overview
Excel	Microsoft	N/A
Zen lite	Zeiss	https://www.zeiss.com/microscopy/us/products/software/zeiss-zen.html
ImageJ	NIH	https://imagej.nih.gov/ij/
El-MAVEN	Agrawal et al, 2019	N/A

### Mice

The *Cdc42*^flox^ mice (Wu et al, 2006), *Cdc42*-V2^Tg^ (Zhang et al, 2020), *Villin*-CreER (El Marjou et al, 2004), *Lyz1*^3’UTR-IRES-CreER^ (Balasubramanian et al, 2023), *Rosa26R*^tdTomato^ mice (Madisen et al, 2010) (JAX# 007914) have been previously described and characterized. A colony of PC reporter mice (*Lyz1*^3’UTR-IRES-CreER^; *Rosa26R*^tdTomato^) was rederived into germ-free (GF) condition at the National Gnotobiotic Rodent Resource Center at UNC-Chapel Hill, USA. All mice were developed and maintained on a C57BL/6 genetic background, on a 12-h light, 12-h dark cycle and provided with food and water ad libitum in individually ventilated cages under specific pathogen-free (SPF) or gnotobiotic facilities at the Rutgers University animal facility. All experiments were performed using age-matched littermates of both sexes between 8 and 16 weeks of age. Mouse candidates were randomly allocated to experimental groups prior to treatment or infection.

### Mouse organoid culture, tdTomato induction, and treatment with reactive oxygen species and cytokines

Small intestinal crypts were isolated from adult mice as previously described (Sato et al, 2009) and cultured in organoid growth medium (1x B27, 1x N2, 1 mM NAC, 1 µg/ml R-spondin, 100 ng/ml Noggin, 50 ng/ml EGF, 100 µg/ml Primocin, 1x GlutaMax, and 10 mM HEPES in Advanced DMEM/F-12). The crypts were counted and suspended in Matrigel mixed with medium (4:1 ratio) at a density of 5 crypts/µL. The suspension was seeded into an 8-well chambered cover glass at 20 µL per well. The medium was changed daily to maintain organoid culture. On day 3, organoids were treated overnight with 1 µM 4-hydroxytamoxifen (4-OHT) and then switched back to regular organoid growth medium for a 12-h recovery period. Following recovery, organoids were treated with rotenone, FCCP, or H₂O₂ for 24 h. For short-term experiments, organoids under treatment were incubated with 10 µM EdU for 1 h and then fixed for immunofluorescence. For long-term experiments, the treatments were withdrawn, and organoids were cultured in regular organoid growth medium for an additional 3 days before EdU labeling and fixation. Cytokines were used in the experiments at the following concentrations: IFN-γ (1 or 2 ng/mL), IL-17 (50 ng/mL), and IL-22 (4 ng/mL).

### Immunofluorescence for fixed enteroids

Organoids embedded in Matrigel were fixed with 2% paraformaldehyde at room temperature for 30 min, followed by permeabilization with 0.5% Triton X-100 in PBS. After permeabilization, organoids were washed with wash buffer (2.5% BSA, 0.05% Tween-20 in PBS) and then incubated in blocking solution containing 2.5% normal donkey serum in wash buffer at room temperature for 1 h. Blocked organoids were incubated with primary antibodies overnight at 4 °C. The next day, organoids were washed with wash buffer and incubated with secondary antibodies at room temperature for 2 h. After washing, organoids were mounted and imaged using a ZEISS LSM 980 microscope with Airyscan 2.

### Live cell imaging on enteroids stained with MitoTracker

Duodenal crypts from *Cdc42*^WT^, *Cdc42*^ΔPC^, and PC-V2^Tg^ mice were counted and suspended in Matrigel mixed with medium (1:1 ratio) at a density of 5 crypts/µL. The suspension was seeded into an 8-well chambered cover glass as described. The enteroids were treated with 0.25 µM 4-OHT from day 3 to day 5. On day 5, the enteroids were washed twice with warm PBS and incubated in HBSS buffer (with calcium and magnesium) containing 100 nM MitoTracker Deep Red FM (Thermofisher, M22426) for 30 min in a cell culture incubator. After incubation, the enteroids were washed twice with warm PBS and imaged using a ZEISS LSM 980 microscope equipped with Airyscan 2 while maintained at 37 °C and 5% CO_2_.

### Paneth cell sorting from enteroids for untargeted LC-MS metabolomic analysis

Three PC reporter mice were administered a single dose of 75 mg/kg tamoxifen through intraperitoneal injection. Two days post-injection, duodenal crypts were isolated, counted, and suspended in Matrigel mixed with culture medium (4:1 ratio) at a density of 5 crypts/µL. The suspension was seeded into 6-well plates at 20 µL/dome (10 domes/well). Three days after seeding, the enteroids were treated with 1 µM 4-OHT. On the following day, the enteroids were treated with PBS, 1 µM FCCP or 1 mM H_2_O_2_, each in the presence of 0.25 µM 4-OHT for 24 h. After treatment, the enteroids were washed twice with warm PBS and cultured in enteroid culture medium supplemented with 17.5 mM D-(+)-Glucose-^13^C_6_ (Cayman, 26707) for 17 h. The enteroids were collected from the plates using Cell Recovery Solution (Corning, 354253) and dissociated into single cells using TrypLE Express (ThermoFisher, 12605028) containing 1 µM Y-27632 dihydrochloride (Sigma, Y0503) at room temperature. The cells were stained with DAPI to exclude dead cells and sorted into DAPI^-^tdT^-^ and DAPI^-^tdT^+^ populations into 1.5 mL Eppendorf tubes.

The sorted cells were washed twice with ice-cold PBS containing 1 µM Y-27632 and then lysed in 1 mL of ice cold extraction solvent (2:2:1 methanol:acetonitrile:water with 0.5% formic acid) by vortexing for 1 min at maximum speed. The lysates were kept on ice for 5 min before 50 µl of 15% NH_4_HCO_3_ was added. The tubes were centrifuged at 15,000 × *g* for 10 min at 4 °C to pellet cell debris and proteins. From each sample, 800 µL of the supernatant was transferred to a new 1.5 mL tube on ice, stored at −80 °C, and submitted to the core facility for LC-MS metabolomic analysis the following day. The LC-MS/MS was performed on a Q Exactive PLUS hybrid quadrupole-orbitrap mass spectrometer coupled to a Vanquish Horizon UHPLC system (Thermo Fisher Scientific). Prior to analysis, 1 µM [^2^H_4_]-lactate was spiked into each sample for absolute quantification of lactate. Metabolite data were analyzed using the El-MAVEN software package.

### In vivo *S. Typhimurium* infections

All animal experiments were conducted according to protocols approved by Rutgers University’s Institutional Animal Care and Use Committee. To induce CreER recombination, a single dose of tamoxifen (75 mg/kg body weight; Sigma, T5648) in corn oil was injected intraperitoneally either 2 days (for Lyz1^3′-UTR-CreER^) or 7 days (for Villin-CreER) prior to infection. Groups of WT and transgenic 13- to 20-week-old mice were orally gavaged with 15 mg of streptomycin (S6501; Sigma) one day prior to infection. Mice were then infected by oral gavage with 10^8^ CFU of wild-type (WT) *S. Typhimurium* SL1344, or 200 µL PBS as controls. Infected animals were monitored, and body weight was recorded for up to 6 days post infection. Blood, colonic feces, spleens, livers, and the entire intestinal tract were aseptically harvested upon sacrifice.

### Analysis of systemic bacterial burden

Blood was removed aseptically by cardiac puncture in 5 mM EDTA. *S. Typhimurium* CFU in blood were determined by plating whole blood as well as serial dilutions on XLD Agar plates, which were incubated at 37 °C overnight for growth. Feces, spleens, livers, and mesenteric lymph nodes were removed aseptically, and 5–10 µl PBS per mg tissue was added to each sample, followed by homogenization at 4 °C. Serial dilutions of each homogenate were plated on XLD agar plates, followed by overnight incubation at 37 °C and CFU quantification the following day. Minimal detectable CFU values were as described (Barthel et al, 2003). Briefly, these were 5 CFU/fecal pellet, 20 CFU/mg spleen, 100 CFU/mg liver, and 10 CFU/mg of mesenteric lymph nodes.

### Intestinal permeability analyses

Two hours prior to sacrifice, food was removed, and mice were orally gavaged with 60 mg/100 g body weight fluorescein isothiocyanate–dextran (FITC-Dextran) (Sigma, FD4). Upon sacrifice, blood was removed aseptically by cardiac puncture in 5 mM EDTA and centrifuged at 3000 × *g* for 5 min. Plasma was separated from the blood cell pellet for each sample. For quantification of FITC-Dextran in plasma samples, a standard curve was created through serial dilutions from 0.1 picogram/mL to 1 nanogram/mL in control plasma. 100 µl of each standard and each experimental sample were transferred to a black-opaque-bottomed 96-well plate. A Promega^TM^ GloMax^®^ Plate Reader was used to determine fluorescence in samples with an excitation at 485 nm and emission at 530 nm.

### Droplet-based single-cell RNA sequencing

Paneth cell number and viability were determined by a Propidium Iodide-based fluorescence assay using the Moxi GO II System, Orflo Prod#MXG102 (ORFLO Technologies, LLC). Droplet-based single-cell partitioning and single-cell RNA-Seq libraries were generated using the Chromium Single-Cell 3′ Reagent v3 Kit (10X Genomics, Pleasanton, CA) on the 10X Chromium Controller as per the manufacturer’s protocol. Live cells in single-cell suspension with 95% viability were mixed with Gel beads, RT reagents, and Partitioning Oil into a Single-Cell 3′ Chip and loaded onto a 10X Chromium Controller for GEM generation. Briefly, the protocol includes RT, cleanup, cDNA amplification, fragmentation, end repair & A-tail prep, adapter ligation, and incorporation of sample indices into finished libraries, which are compatible with Illumina next-generation sequencing platforms. Sample quantification and quality control were determined using a Qubit Fluorometer (Invitrogen, Life Technologies) and TapeStation (Agilent Technologies, Santa Clara, CA), respectively. cDNA libraries were sequenced on an Illumina NovaSeq 6000 sequencer (Illumina, San Diego, CA) with a configuration of 28/8/0/91-bp for cell barcode, sample barcode, i5 index, and mRNA reads, respectively, as a recommendation by 10X Genomics. The Chromium Single Cell Software is used to analyze and visualize single-cell 3’ RNA-seq data produced by the 10X Chromium Platform. The 10X Chromium software package includes Cell Ranger Pipelines and Loupe Cell Browser. Cell Ranger pipelines use raw 10X single-cell sequencing data from an Illumina sequencer and perform demultiplexing, unique barcode processing, and single-cell 3’ gene counting.

### Computational analyses of scRNA-seq expression data

Single-cell RNA-seq samples were processed using Seurat v5 (Hao et al, 2024). For each sample, cells with fewer than 1000 detected genes, more than 90,000 unique molecular identifiers (UMIs), or over 10% of counts originating from mitochondrial genes were excluded. Doublets were predicted using DoubletFinder v2.0.4 (McGinnis et al, 2019) and subsequently removed. Cell cycle phase scores were calculated based on mouse orthologs of canonical human S and G2/M genes. After quality control, samples were integrated and normalized using SCTransform (Hafemeister and Satija, 2019), with cell cycle effects regressed out during normalization. Principal component analysis (PCA) was performed, and the top 5 principal components were selected based on the elbow point in the standard deviation plot for Louvain clustering. CytoTRACE2 v1.0 (Kang et al, 2025) was used to compute potency scores for each cell, enabling identification of progenitor and differentiated Paneth cell clusters. Trajectory analysis was performed using monocle3 v1.3.7 (Trapnell et al, 2014), with the progenitor cluster set as the starting root of the pseudo-time axis. A generalized linear model was then fitted for each gene to identify those whose expression levels significantly covaried with pseudo-time. Pathway activity scores were computed using AUCell v1.26 (Aibar et al, 2017) with hallmark gene sets from the Molecular Signatures Database (MSigDB) (Liberzon et al, 2015).

### Bulk RNA-Seq of mouse intestines and Gene Set Enrichment Analysis

RNA-Seq data of WT and *Cdc42*-deficient IECs were previously deposited at the National Center for Biotechnology Information’s BioProject with accession number GSE124848 (Ladinsky et al, 2019; Zhang et al, 2020). For Gene Set Enrichment Analysis (GSEA) (Subramanian et al, 2005) performed on the antimicrobial peptide list and reactive oxygen species pathway, pre-ranked files of differentially expressed genes were calculated by the rank metric = −log(*p*-value) *SIGN (logFC) (Jia et al, 2015). Normalized enrichment scores (NES) and *p*-values were documented (K-S Test).

### Genomic DNA extraction for nanopore, data processing, multi-omic visualization

Paneth cells were isolated from the small intestine of GF and SPF PC reporter mice using fluorescence-activated cell sorting (FACS). Genomic DNA was extracted from the sorted Paneth cells using the Qiagen AllPrep DNA/RNA Kit, following the manufacturer’s instructions. For Nanopore data processing, sequencing data were basecalled and modified bases were identified using Dorado with the model dna_r10.4.1_e8.2_400bps_hac@v4.3.0_5mC_5hmC@v1. Reads were aligned to the mm10 mouse reference genome. The resulting aligned BAM files containing base modification information were processed using the DSS R package to identify differentially modified regions/sites (DMRs/DMSs). Visualization was performed using the NanoMethViz R/Bioconductor package (Su et al, 2021; Wu et al, 2015). Multi-modal data integration and visualization, including scRNA-seq, ATAC-seq, and Nanopore nascent DNA sequencing, were carried out using the R packages NanoMethViz and ggcoverage (Song and Wang, 2023). Composite figures were generated using the patchwork package in R to combine individual panels.

### Paneth cell ATAC-sequencing analysis

For ATAC-seq, freshly FACS-isolated tdTomato^+^ live Paneth cells from GF or SPF Paneth cell reporter mice were processed according to the OMNI-ATAC-Seq protocol (Corces et al, 2017). Briefly, approximately 50,000 Paneth cells were washed in cold PBS and resuspended in 50 µL of ATAC Resuspension Buffer (RSB) (10 mM Tris-HCl pH 7.4, 10 mM NaCl, 3 mM MgCl_2_, 0.1% IGEPAL CA630, 0.1% Tween-20, 0.01% digitonin). Cells were incubated on ice for 3 min followed by a wash step with 1 mL of RSB containing only 0.1% Tween-20 and a centrifugation step at 500 × *g* for 10 min at 4 °C. Nuclear pellets were treated with 50 µL Nextera Tn5 Transposase mixture (Illumina) for 30 min at 37 °C. Samples were then purified with the Zymo DNA Clean and Concentrator-5 kit and pre-amplified using the NEBnext 2X Master Mix for 5 cycles. 5 µL of the pre-amplified sample was subjected to qRT-PCR amplification for an additional 20 cycles to determine the optimal additional cycles of the remaining 45 µL of sample. The PCR-amplified libraries were purified; fragment size was selected using Pippin Prep and sequenced on an Illumina NextSeq 150 cycle mid output. For ATAC-seq analysis: paired-end ATAC-seq fastq files were quality checked using fastQC. Nextera transposase adapter reverse complement was removed from the 3′ end of the ATAC-Seq reads using CutAdapt, version 1.9.1. Trimmed reads were aligned to the mouse genome build mm9 using Bowtie2. Picard (v2.18.27) was used to determine the median alignment size of each alignment bam file. Peak region bed files were called from each alignment bam file using MACS2 (v2.1.0). MACS2 was run with a “shift” distance of −0.5 times the median alignment size and an “extsize” distance equal to the median alignment size for each alignment bed file. Peak files were filtered against the ENCODE blacklist (Amemiya et al, 2019) and BEDtools (Quinlan and Hall, 2010) was used to subtract the sites on the ENCODE blacklist. The Integrative Genomics Viewer (IGV 2.4.13) (Robinson et al, 2011) was used to visualize ATAC-seq bigwig tracks. DiffBind (v2.4.7) FDR < 0.01 as the cutoff for significance.

### Immunofluorescent staining and immunohistochemistry

Intestinal tissues were collected and fixed in 10% neutral formalin buffer overnight at 4 °C and embedded in paraffin. 4–5 µm sections were sliced and dewaxed. Slides were immersed into antigen retrieval buffer at a sub-boiling temperature for 15 min. Sections were then blocked in PBS containing 0.1% Triton X-100, 2% BSA and 2% normal serum for at least 1 h at room temperature, and then probed with primary antibodies at 4 °C overnight for the following antigens (anti-E-cadherin, 1:500, BD Biosciences 610182; anti-Lysozyme, 1:200, Biogenex AR024-10R; anti-Mmp7, 1:100, Cell Signaling Technologies 3801T; anti-Muc2, 1:10,000, Abcam ab272692, anti-DCLK1, 1:1000, Abcam ab109029; anti-Olfm4, 1:200, Cell Signaling Technologies 39141; anti-PCNA, 1:200, SantaCruz sc-56; rabbit anti-RFP, 1:200, Rockland 600-401-379; goat anti-RFP, 1:100, Rockland 200-101-379; anti-8’OHdG, 1:200, Abcam ab48508. The following morning, slides were washed in PBS three times and probed with biotinylated secondary antibody (for IHC) or fluorescence-conjugated secondary antibodies with DAPI counter-staining (for immunofluorescence). After 1-h incubation at room temperature, slides were washed for three times. For immunofluorescence, slides were air-dried and mounted with Prolong Gold antifade medium. For IHC, slides were incubated with streptavidin-conjugated horseradish peroxidase (HRP) for an hour at room temperature and DAB HRP substrate kit was used for development. All images were obtained using ZEISS LSM 980, and LSM 510, Nikon Eclipse TE2000. Micrographs were analyzed using ImageJ Fiji software.

### Western blotting

Mouse intestinal tissues were lysed in RIPA buffer (Thermofisher, 89901) with proteinase inhibitor (Sigma, 11836170001). The following primary antibodies were used for immunoblotting: NRF2 (Invitrogen, PA5-27882; 1:1000), p62 (Abnova, H00008878M01; 1:1000), GAPDH (Gene Tex, GTX627408; 1:2000), lysozyme (Invitrogen, PA1-29680; 1:1000), CDC42 (Cell Signaling Technology, 2462S; 1:1000), and MPTX1/2 (Abcam, ab238123; 1:2500).

### Dot blotting

Dot blots were done according to methods found in Gonzalez et al (2023). In brief, DNA was purified from the samples using tail lysis buffer with Proteinase K and precipitated via isopropanol. Following washes with 70% ethanol, DNA was resuspended in TE Buffer. Samples were subjected to light sonication using a qSonica Q800R at 4 °C for 20 min to fragment the DNA. Serial dilutions were made from 800 ng to 100 ng using DNA in TE buffer and denaturing buffer (1 M NaOH, 25 mM EDTA), followed by denaturing them at 95 °C for 10 min. Ice cold 2 M Ammonium Acetate (pH 7.0) was added to the samples, which were maintained on ice for 10 min. The nitrocellulose membrane was prepared by washes with ddH_2_O and 6x SSC buffer. The dot blot apparatus was assembled and hydrated with TE buffer prior to loading samples. After the samples were passed through, 2x SSC was used to wash the wells and membrane following the disassembly of the apparatus. The membrane was air-dried before being subjected to UV crosslinking on both sides for 10 min total. The membrane was blocked using 5% skim milk in TBST for 1 h and 5hmC primary antibody in 5% skim milk in TBST (1:5000, Active Motif, 39999) was added for incubation overnight. Membrane was washed 3x for 20–30 min in TBST, and a goat anti-mouse HRP-conjugated secondary antibody (1:5000, BioRad, 1706516) in 5% milk in TBST was incubated for 1 h. The membrane was washed three times for 20–30 min and was developed using chemiluminescence (Thermofisher, 34096). Post development the membrane was washed with deionized water before being subjected to methylene blue staining (0.1% methylene blue, 0.5 M Sodium Acetate pH 5.2) for 10–15 min and subsequent washes with deionized water. Chemiluminescent and methylene blue images were quantified by densitometry via ImageJ.

### Transmission electron microscopy

The procedure for transmission electron microscopy was described previously (Balasubramanian et al, 2023; Yu et al, 2014). *Cdc42*^WT^*, Cdc42*^ΔPC^, and PC-V2^Tg^ mice were injected with tamoxifen (75 mg/kg, intraperitoneally) once daily for 3 consecutive days. Three days after the final injection, all the mice were euthanized and around 1 mm of their distal ileum was fixed in 0.1 M sodium cacodylate containing 2% glutaraldehyde (EM grade) and 2% paraformaldehyde (EM grade) overnight at 4 °C on a shaker. The fixed samples were washed twice with 0.1 M sodium cacodylate, then post fixed with 1% buffered OsO_4_ and stained with 1% uranyl acetate. These samples were then dehydrated using increasing concentrations of ethanol followed by propylene oxide. The samples were further infiltrated with EMbed-812/propylene oxide (1:1) and 100% EMbed-812, after which they were embedded using the EMbed-812 kit (Electron Microscopy Sciences, Catalog No. 14120).

### PCR validation of *Cdc42* deletion in Paneth cells

A forward primer was designed upstream of the 5’ LoxP site and a reverse primer was designed downstream of the 3’ LoxP site. Between primers, the WT sequence is 744 bp, the excised sequence is ~350 bp, and the sequence with deletion is ~400 bp. Forward (F) and reverse (R) primers are as follows: F: 5’ CTATACTTCATATACCACACG 3’; R: 5’ TCTGCCATCTACACATACAC 3’. The reaction was as follows: 95 °C for 5 min, followed by 40 cycles of 95 °C for 45 s, 52 °C for 45 s, and 72 °C for 90 s, with a final step of 72 °C for 5 min.

### Dihydroethidium (DHE) staining

Freshly isolated whole ilea of *Cdc42*^WT^ and *Cdc42*^ΔPC^ were washed in PBS and embedded in O.C.T. (Tissue-Tek). Tissues were immediately sectioned 30 µm thick on a cryostat (Leica Biosystems, Buffalo Grove, IL, USA), mounted onto slides and incubated at 37 °C for 30 min in 10 µM DHE (Millipore Sigma, #37291-25MG). Tissues were then washed 3 times in PBS and mounted with Prolong Gold antifade medium. Slides were then imaged on the LSM 510 Laser Scanning Microscope with an excitation/emission of 390 nm/415 nm.

### Epithelial isolation for flow cytometry analysis

The isolated crypts were resuspended in 2 mL TrypLE Express (ThermoFisher, 12605028) containing 273 Kunitz units of DNase I (Qiagen, 79254) and 1 µM Y-27632 dihydrochloride (Sigma, Y0503) and incubated at room temperature for 4 min with pipetting 5 times per minute. After digestion, the cell suspension was diluted in 10 mL FACS buffer (F12/DMEM containing 2 µM ROCK inhibitor, 2% BSA, 2% FBS, and 2 mM EDTA), passed through a 40-µm filter, and centrifuged at 500 × *g* for 5 min at 4 °C. Cell pellets were washed with 2% FBS in PBS containing 2 µM Y-27632 and stained with LIVE/DEAD™ Fixable Violet in this buffer (ThermoFisher L34955). After washing with FACS buffer, cells were stained for surface markers in FACS buffer at 4 °C for 30 min. This was followed by a wash and incubation with 500 nM CellROX Green reagent (ThermoFisher, C10444) in HBSS (with Ca^2+^ and Mg^2+^) containing 1 µM Y-27632 for 30 min at 37 °C. Cells were then washed, fixed with 2% paraformaldehyde and stained for intracellular markers in FACS buffer containing 1× permeabilization buffer (ThermoFisher, 00-8333-56) overnight at 4 °C. Flow cytometry was performed on a BD LSRFortessa X-20 (BD Biosciences), and imaging flow cytometry was conducted using the AMNIS ImageStream^X^ Mark II (Amnis Corporation).

### Quantification and statistical analyses

All graphic data and statistical analyses were conducted using GraphPad Prism 11 (https://www.graphpad.com) and Microsoft Excel. All graphs contain individual data points aggregated from at least 3 independent experiments. Animal numbers are indicated on graphs reporting the experimental results. The data were reported as mean ± SEM. Statistical comparisons for two groups were performed by an unpaired Student’s *t*-test, and those for multiple groups were performed by a one-way or two-way ANOVA. To minimize subjective bias, investigators were blinded to sample identities during image quantification and LC-MS metabolomics analysis.

## Supplementary information

Peer Review File

Source data Fig. 1

Source data Fig. 2

Source data Fig. 3

Source data Fig. 4

Source data Fig. 5

Source data Fig. 6

Source data Fig. 7

Expanded View Figures

## Data Availability

The datasets and computer code produced in this study are available in the following databases: Paneth cell scRNA-Seq datasets: Gene Expression Omnibus GSE237329. Paneth cell-specific ATAC-seq datasets: Gene Expression Omnibus GSE302951. Paneth cell Nanopore sequencing datasets: Gene Expression Omnibus GSE301987. Enteroids LC-MS datasets: Computational Mass Spectrometry MSV000101844. Script and orthologs files for the scRNA-seq analysis: GitHub (https://github.com/Yeqing95/Paneth_Cdc42_project). The source data of this paper are collected in the following database record: biostudies:S-SCDT-10_1038-S44318-026-00869-y.
