# Supplementary material for: Reactive oxygen species stimulate Paneth cell plasticity and diminish antimicrobial function
Source: EMBO J. 2026 Jul 18;45(19):6723–58. doi: 10.1038/s44318-026-00869-y (PMC13631327; doi:10.1038/s44318-026-00869-y)
Supplement: Supplementary file 1 [file 44318_2026_869_MOESM1_ESM.pdf]

THE EMBO JOURNAL

Reactive oxygen species stimulate Paneth cell plasticity and diminish antimicrobial function

Jiaxing Yang, Sheila Bandyopadhyay, Dahui Wang, Edward Gonzalez, Yeqing Chen, Jagannatham Bhupana, Oscar Pellon-Cardenas, Helen Park, Iyshwarya Balasubramanian, Jared Bianchi-Smak, Xiao Zhang, Zhi Wei, Edward Bonder, Michael Verzi, Jean-Pierre Etchegaray, and Nan Gao

Corresponding authors: Nan Gao (ngao@njms.rutgers.edu)

Review Timeline:

Submission Date:	26th Nov 25
Editorial Decision:	21st Jan 26
Revision Received:	25th May 26
Editorial Decision:	20th Jun 26
Revision Received:	1st Jul 26
Accepted:	6th Jul 26

Editor: Ieva Gailite

Transaction Report:

(Note: With the exception of the correction of typographical or spelling errors that could be a source of ambiguity, letters and reports are not edited. Depending on transfer agreements, referee reports obtained elsewhere may or may not be included in this compilation. Referee reports are anonymous unless the Referee chooses to sign their reports.)

1st Editorial Decision 21st Jan 2026

Dear Nan,

I hope you have had a good start into the year! Thank you for submitting your manuscript for consideration by the EMBO Journal. I sincerely apologise for the protracted assessment process of your manuscript due to the holiday period and the resulting delays in reviewer report submission. We have now received comments from a full set of reviewers, which are included below for your information.

As you will see, all reviewers are generally positive in their evaluation and appreciate the contribution of the study to the research field. They also indicate a number of constructive concerns that would need to be addressed before they can support publication here. From my side, I find these comments generally reasonable. Therefore, I invite you to submit a revised manuscript in response to the comments by all reviewers. I would also be happy to discuss the revision in more detail via email or phone/videoconferencing.

We generally allow three months as standard revision time, which can be extended to six months in the case of major revisions. Should you foresee a problem in meeting this deadline, please let us know in advance to discuss an extension.

As a matter of policy, competing manuscripts published during this period will not negatively impact on our assessment of the conceptual advance presented by your study. However, please contact me as soon as possible upon publication of any related work to discuss the appropriate course of action.

When preparing your letter of response to the referees' comments, please bear in mind that this will form part of the Review Process File and will therefore be available online to the community. For more details on our Transparent Editorial Process, please review our Editorial Policies: https://link.springer.com/partners/embo-press/editorial-policies. Please also see the attached instructions for further guidelines on preparation of the revised manuscript.

Please feel free to contact me if you have any further questions regarding the revision. Thank you for the opportunity to consider your work for publication. I look forward to discussing your revision.

With best wishes,

Ieva

Ieva Gailite, PhD
Senior Scientific Editor
The EMBO Journal
Meyerhofstrasse 1
D-69117 Heidelberg
Tel: +4962218891309
i.gailite@embojournal.org

Instructions for preparing your revised manuscript:

Read our guidance for manuscript revisions and related editorial policies: https://link.springer.com/journal/44318/submission-guidelines#cms-Revised-submissions

Please make sure you upload a letter of response to the referees' comments together with the revised manuscript.

Please also check that the title and abstract of the manuscript are brief, yet explicit, even to non-specialists.

When assembling figures, please refer to our figure preparation guideline in order to ensure proper formatting and readability in print as well as on screen:
https://media.springernature.com/original/springer-cms/rest/v1/content/27825798/data/v1

At EMBO Press we ask authors to provide source data for the main manuscript figures. You will receive a separate email with instructions for providing source data with your revised manuscript, including how to upload and organize the files.

IMPORTANT: When you send the revision we will require
- a point-by-point response to the referees' comments, with a detailed description of the changes made (as a word file).
- a word file of the manuscript text.

- individual production quality figure files (one file per figure)
- a complete author checklist
- Expanded View files (replacing Supplementary Information)
- a Reagents and Tools Table as part of the Methods section

Please remember: Digital image enhancement is acceptable practice, as long as it accurately represents the original data and conforms to community standards. If a figure has been subjected to significant electronic manipulation, this must be noted in the figure legend or in the 'Materials and Methods' section. The editors reserve the right to request original versions of figures and the original images that were used to assemble the figure.

We realize that it is difficult to revise to a specific deadline. In the interest of protecting the conceptual advance provided by the work, we recommend a revision within 3 months (21st Apr 2026). Please discuss the revision progress ahead of this time with the editor if you require more time to complete the revisions.

Referee #1:

In this manuscript, the authors provide a detailed investigation into how oxidative stress affects Paneth cell plasticity and function. The authors use scRNA-seq, flow cytometry, and organoid cultures to show that a large proportion of Paneth cells are highly tolerant of oxidative stress and can be stimulated into the cell cycle by reactive oxygen species and oxidative stress. In addition, Paneth cell-specific knockout and overexpression mouse models were used to show that Cdc42 is involved in regulating the balance between oxidative stress response and antimicrobial production. The study represents an interesting and novel approach to better understand how Paneth cells respond to oxidative stress. The use of lineage tracing in these experiments represents a step forward in understanding the behavior of Paneth cells under oxidative stress and antimicrobial production. The involvement of Cdc42 in regulating the balance between oxidative stress response and antimicrobial production in Paneth also represents an advance in understanding the function of this protein in Paneth cells. The study is well thought out and executed; additional experiments are suggested that might add to the work.
Major comments:

• Paneth and goblet cells are thought to emerge from a common ancestor and have been shown to undergo fate switching under a variety of stress conditions. Although the experiments study Paneth cell maturation and how oxidative stress affects this, the authors do not examine how this affects goblet cells and whether fate switching is occuring. Multiple types of stress conditions have been known to trigger dedifferentiation of Paneth cells to a Paneth/goblet intermediate cell state marked by the appearance of both lysozyme and mucin granules. This is often characterized by co-staining of lysozyme and goblet cell markers like Muc2 or TFF3 in Paneth cells. Performing additional immunofluorescence experiments to determine Muc2, TFF3 levels in Paneth cells would be helpful. It would enhance the work if the authors were to determine if increasing oxidative stress and/or reactive oxygen species triggers the appearance of these intermediate cells and how this fits into the dedifferentiation-mediated entrance into the cell cycle of Paneth cells.
• Further characterization, using transmission electron microscopy, of the changes occurring to the secretory granules following increased oxidative stress and exposure to ROS would also be helpful. This type of analysis will also aid in understanding more precisely what is occurring when Paneth cells lose their normal morphology and adopt a slender one that appears to be devoid of secretory granules.
Minor comments
• There were a few places where there was no space between the end of a sentence and the reference.
• It was generally unclear how much tamoxifen was given (and by what route) and how often to induce lineage tracing in vivo. Please add this information to the figures where appropriate.
• In the "Infection exacerbates oxidative stress in Cdc42ΔPC mice" paragraph references Figure 6F and 6H and it reads like it should be Figure 6H and 6D.
• In the "The oxidative stress in Cdc42ΔPC mice cause damage and death to the neighboring crypt cells" paragraph the Figure 7D and Extended Fig. 7A reference should read Figure 7E and Extended Fig. 7A.

Referee #2:

Increasing evidence suggests that oxidative stresses influence gut stem cell activity. Performing single cell analysis of Paneth cells and organoid culture, the authors showed that oxidative phosphorylation activity declines, as Paneth cells become matured, while reactive oxygen species can activate Paneth cell proliferation. Employing gain and loss of function studies in vivo, the authors demonstrate that Rho GTPase CDC42 regulates Paneth cells' oxidative response. Together, this study provides new insight into Paneth cells' response to oxidative stresses. Since oxidative stress response is known to be activated in

inflammation and infection, their work would likely be useful for the development of new therapies targeting oxidative stress pathways and Paneth cells' function. The following suggestions may further improve the current manuscript.

The authors showed that the strong expression of Gpx2, which is known to catalyzes the reduction of hydrogen peroxide, in the immature Paneth cells. How does this expression pattern explain high levels of oxidative respiration activity in immature Paneth cells, compared to mature Paneth cells? Shouldn't Gpx2 be able to reduce oxidative stress?

In Figure 2, the authors utilized organoid culture and demonstrated that exogenous and mitochondrial reactive oxygen species drive Paneth cells into cell cycle. This data is very interesting. What percent of immature Paneth cells are proliferating? How is their proliferation regulated despite of the high levels of ROS and OXPHOS activity. Could Paneth cells entering cell cycle also give rise to Lg5+ stem cells and their progeny in vivo (the authors could address this question by performing their tdTomato lineage tracing)? If so, this would demonstrate dedifferentiation of Paneth cells back to stem cells by ROS and OXPHOS activity.

Elegant mouse genetic studies demonstrated that CDC42 regulates Paneth cells' oxidative response. However, it remains unclear how CDC42 regulates their response? How does CDC42 suppress ROS and OXPHOS activity in Paneth cells?

Interestingly, Cdc42 deletion reduced OLFM4+ stem cell numbers (Figure 7C). How does CDC42 control stem cells (via ROS and OXPHOS activity or not)? Did also the addition of reactive oxygen species to organoid culture also impair stem cells? If so, could this stem cell impairment rescued by modulating ROS and OXPHOS activity?

Referee #3:

The paper discusses interesting aspects of Paneth cells, which are under-represented in biomedical research, despite they are really essential. The paper is generally well written, and the figures are good quality. The story is of interest and this journal looks ideal for it to be published when certain aspects have been corrected and addressed.
1. Though I wrote that the paper is well written, there are a relatively high amount of language errors and the paper should be well edited. For example, in the abstract, there are some grammatical issues (two times a 'the' where inappropriate).
2. The Paneth cell-specific cre mouse that was used looks like a great tool. Probably it was explained in the original 2023 paper, but also for this paper it is essential to know what fraction of site-specific recombination is found after TAM injection in the mice. Are all PCs showing TdT expression after TAM? And what about Cdc42 after TAM injection?
3. Also in relation to this crucial tool: The Lyz1 gene is strongly expressed in PCs. That is a fact, and thus the expression of Cre from that gene's promotor looks logical. However, Lyz1 is known to show ectopic expression in other cells (such as, but not limited to, Alveolar Macrophages, see PMID: 40636116). In this paper, it might not matter so much (although AMs might play a role in Salmonella survival), but it should be stated clearly.
4. There is crucial info lacking on how the PCs were isolated from mice. It looks like this is done via TdT and via FACS. As the authors will have read in the literature (PMID: 39273007), there is significant difference in PC transcriptomes in relation to the source of the PCs (duodenum, jejunum or ileum), so it has to be stated very clearly in results, figure legends and Materials where exactly the PCs were recruited and how many cells were obtained.
5. I can see the point of the UMAPs in Fig.1, I am just wondering if the transcripts are the best ones to choose. I think the authors should add Lyz1 and TdT as positive controls, as well as Defa24 or Defa30, because these last ones are the strongest expressed out of the 28 Defa genes, to make sure all cells are really PCs, because I have my doubts given the previous point, and given the Fig1P and others, which may seem to suggest that some stem cells were co-sorted. A UMAP figure with data of Defa24 or Defa30 (or CD24) would be reassuring (Defa27 is one of the weakest Defa genes expressed in PCs (See PMID: 39981239).
6. The fact that PCs, when maturing, have less TCA activity is well-known from the work of Riccardo Fodde. It is also considered that this is an essential step for PCs to produce lactate from pyruvate (and not send the pyruvate to the mitochondria, in order to sustain the stemness of these neighboring stem cells. The authors must consider this in their paper. Like everything in life, also for PCs, they must give and take something at the cost of something. Oxidative stress (as given here by hydrogen peroxide, very harsh) is a part of live and PCs use this to get their function done, maybe by the cost of some Defa expression, but honestly, this is the result of millions of years of evolution and seems a good thing.
7. Fig. 4B, this says 'probability to die'. What is this? Is this not a real survival curve? How is the probability calculated or so? Why is the survival curve stopped at Day5? Are there no further datapoints? Or did no further deaths occur? This issue about Day5 also applies to other curves in this paper. And Fig4C lacks a legend on the Y axis.
8. Figure 5, I have the same question as before: after TAM, if the PCs were sorted on TdT signal, of course only cells that showed cre activity are sorted, and will be cdc42 KO, which provides a beautiful PCR gel, but this is not correct to estimate the KO in all PCs of the mice after TAM. A classical sort via CD24 and c-Kitligand should be done, or another technique, maybe RNAscope, to investigate the degree of Cdc42 in the IECs.
9. Fig. 5G: this is interesting, and should be confirmed with a Lyz1 staining and Defa24 (or 27) staining, perhaps via RNAscope. This is essential. Right now, the data suggest there is TdT expression via Cre activity, thus from Lyz1 promotor activity, but if this means that these signals are coming from new PCs developed should be confirmed, as written here, maybe also via TEM.
10. Fig6B: this is a key figure, yet it is not optimal. It has low numbers of mice, and the experiment stops too soon. There is a * there, but the biological significance is very uncertain. Why are there no body weights here. This figure, along with the cdc42

VillKO rather suggest a very limited role of PCs in this story (the survival to Salmonella).
11. The story lacks a convincing link between the cdc42 part and the possibility of animals or PCs to kill bacteria. One possibility is to purify PCs, lyse them and confront them with bacteria, as published by some groups. There should also be a look at MMP7 activity in this study, for obvious reasons.
12. Fig1P (and elsewhere): this is a picture, so what does it mean when it is written in the figure legend "Data represent 5 independent mice"

POINT-BY-POINT RESPONSE

Referee #1:

In this manuscript, the authors provide a detailed investigation into how oxidative stress affects Paneth cell plasticity and function. The authors use scRNA-seq, flow cytometry, and organoid cultures to show that a large proportion of Paneth cells are highly tolerant of oxidative stress and can be stimulated into the cell cycle by reactive oxygen species and oxidative stress. In addition, Paneth cell-specific knockout and overexpression mouse models were used to show that Cdc42 is involved in regulating the balance between oxidative stress response and antimicrobial production. The study represents an interesting and novel approach to better understand how Paneth cells respond to oxidative stress. The use of lineage tracing in these experiments represents a step forward in understanding the behavior of Paneth cells under oxidative stress and antimicrobial production. The involvement of Cdc42 in regulating the balance between oxidative stress response and antimicrobial production in Paneth also represents an advance in understanding the function of this protein in Paneth cells. The study is well thought out and executed; additional experiments are suggested that might add to the work.

Major comments:
• Paneth and goblet cells are thought to emerge from a common ancestor and have been shown to undergo fate switching under a variety of stress conditions. Although the experiments study Paneth cell maturation and how oxidative stress affects this, the authors do not examine how this affects goblet cells and whether fate switching is occuring. Multiple types of stress conditions have been known to trigger dedifferentiation of Paneth cells to a Paneth/goblet intermediate cell state marked by the appearance of both lysozyme and mucin granules. This is often characterized by co-staining of lysozyme and goblet cell markers like Muc2 or TFF3 in Paneth cells. Performing additional immunofluorescence experiments to determine Muc2, TFF3 levels in Paneth cells would be helpful. It would enhance the work if the authors were to determine if increasing oxidative stress and/or reactive oxygen species triggers the appearance of these intermediate cells and how this fits into the dedifferentiation-mediated entrance into the cell cycle of Paneth cells.

Response: We thank the reviewer for this important point, which we have now addressed using several experimental approaches.

First, we have now performed co-staining for MUC2 on the intestinal tissue sections and found that some lineage-traced dedifferentiated Paneth cells expressed this goblet cell marker (Fig. 6K-L), and we quantified this difference (Fig. 6I).

Second, in the ROS-stimulated enteroid culture experiments, we co-stained for MUC2, and observed that tdT^{+} PC lineage-traced cells contained MUC2+ cells, in addition to MUC2- cells (Fig. 3J), suggesting that the dedifferentiated PCs become multiple epithelial cell types that include MUC2 producing goblet-like cells.

Third, we did transmission electron microscopic (TEM) analysis on WT and Cdc42ΔPC mice and illustrated the changed granule phenotypes in some of the Cdc42-deficient Paneth cells (Fig. 5J, Expanded View Fig. 5A-B).

From these additional data, Paneth cells, when stimulated to dedifferentiate by ROS or by Cdc42 deletion, can become goblet-like cells at molecular and morphological levels. This new information helped enhance the manuscript, thank you.

• Further characterization, using transmission electron microscopy, of the changes occurring to the secretory granules following increased oxidative stress and exposure to ROS would also be helpful. This type of analysis will also aid in understanding more precisely what is occurring when Paneth cells lose their normal morphology and adopt a slender one that appears to be devoid of secretory granules.

Response: We addressed this granule morphological question with two experimental approaches.

First, using live enteroid imaging, we monitored live Paneth cells, and examined the PC morphology changes along with a MitoTracker Green (Fig. 5N). We also quantified the results in Fig. 5O.

Second, we also performed TEM analysis to examine the Paneth cell granule morphology in WT, PC specific Cdc42-knockout and Cdc42-overexpressing mice. We observed abnormal granules showing hybrid features of Paneth cell and goblet cell granular patterns (Fig. 5J). We also quantified the mitochondrial integrity in the Paneth cells of these mice (Fig. 5G-H).

These new morphological results helped improve the manuscript and linked mitochondrial defects to granular reduction in stressed Paneth cells.

Minor comments

• There were a few places where there was no space between the end of a sentence and the reference.

Response: Fixed.

• It was generally unclear how much tamoxifen was given (and by what route) and how often to induce lineage tracing in vivo. Please add this information to the figures where appropriate.

Response: We added this information to method.

• In the "Infection exacerbates oxidative stress in Cdc42ΔPC mice" paragraph references Figure 6F and 6H and it reads like it should be Figure 6H and 6D.

Response: Fixed.

• In the "The oxidative stress in Cdc42ΔPC mice cause damage and death to the neighboring crypt cells" paragraph the Figure 7D and Extended Fig. 7A reference should read Figure 7E and Extended Fig. 7A.

Response: Fixed.

Referee #2:

Increasing evidence suggests that oxidative stresses influence gut stem cell activity. Performing single cell analysis of Paneth cells and organoid culture, the authors showed that oxidative phosphorylation activity declines, as Paneth cells become matured, while reactive oxygen species can activate Paneth cell proliferation. Employing gain and loss of function studies in vivo, the authors demonstrate that Rho GTPase CDC42 regulates Paneth cells' oxidative response. Together, this study provides new insight into Paneth cells' response to oxidative stresses. Since oxidative stress response is known to be activated in inflammation and infection, their work would likely be useful for the development of new therapies targeting oxidative stress pathways and Paneth cells' function. The following suggestions may further improve the current manuscript.

The authors showed that the strong expression of Gpx2, which is known to catalyzes the reduction of hydrogen peroxide, in the immature Paneth cells. How does this expression pattern explain high levels of oxidative respiration activity in immature Paneth cells, compared to mature Paneth cells? Shouldn't Gpx2 be able to reduce oxidative stress?

Response: This is a great question. Typically, when the cellular oxidative activity is high, the reductive activity will also be high, a phenomenon driven by the cell's need to maintain redox homeostasis. We made this point clear in the revised result section. Oxidative stress activates the nuclear factor erythroid 2-related factor 2 (NRF2), which drives a reductive response by regulating the expression of antioxidant, detoxification, and NADPH-regenerating genes, including the Gpx genes here, to restore cellular redox homeostasis.

In Figure 2, the authors utilized organoid culture and demonstrated that exogenous and mitochondrial reactive oxygen species drive Paneth cells into cell cycle. This data is very interesting. What percent of immature Paneth cells are proliferating? How is their proliferation regulated despite of the high levels of ROS and OXPHOS activity.

Response: In untreated enteroids, the majority of them do not contain proliferative Paneth cells, but after stimulation by ROS or IFN-γ, etc., the percentage can go up to 75% in some conditions (Fig. 2M). In WT mice *in vivo*, 0.4% duodenal Paneth cells are Ki67^{+} while 1.8% ileal Paneth cells are Ki67^{+}. We suspect that these basal levels of Ki67 positivity are linked to the small subset of Paneth cells possessing physiologically higher OXPHOS activity. Our data overall suggest that pathologically elevated ROS activities can further increase Paneth cell proliferation.

Could Paneth cells entering cell cycle also give rise to Lg5+ stem cells and their progeny in vivo (the authors could address this question by performing their tdTomato lineage tracing)? If so, this would demonstrate dedifferentiation of Paneth cells back to stem cells by ROS and OXPHOS activity.

Response: To directly address the reviewer's question regarding Paneth cells stimulated by ROS and OXPHOS activities, we lineage traced Paneth cells in enteroids treated with H_2O_2 or OXPHOS modulators and examined OLFM4 expression in these Paneth cell-derived lineage derivatives. These new experimental data show that indeed a small subset of dedifferentiated Paneth cells gave rise to OLFM4^{+}EdU^{+} cells carrying the lineage reporter

marker (Fig. 2B-C, 2E, 2L, Expanded Figure 2H). In these new image data, the OLFM4 signal was shown in purple.

Elegant mouse genetic studies demonstrated that CDC42 regulates Paneth cells' oxidative response. However, it remains unclear how CDC42 regulates their response? How does CDC42 suppress ROS and OXPHOS activity in Paneth cells?

Response: Intracellular ROS is primarily produced through mitochondrial electron transport chain (ETC) activity, where high-energy electrons leak and reduce molecular oxygen to form superoxide. This process contributes up to 90% of total cellular ROS in many mammalian cell types. In addition, endoplasmic reticulum (ER) stress is another significant source of ROS production. Due to an accumulation of misfolded proteins and the unfolded protein response, ER stress increases ROS generation through oxidizing protein folding and enzymatic reactions. We used two experimental approaches to address the important question raised by this reviewer.

First, we performed TEM analysis on WT, Cdc42ΔPC, and CDC42-overexpressing intestinal tissues, and observed (1) a drastically impaired mitochondrial inner membrane integrity in Paneth cells of Cdc42ΔPC mice; and (2) a dramatic ER stress phenotype in virtually all Paneth cells of Cdc42ΔPC mice. New data are shown in Fig. 5G-K.

Second, using live enteroid imaging and mitochondrial tracking, we found that live Paneth cells from Cdc42ΔPC mouse enteroids showed a very low MitoTracker signal that could be due to metabolic stresses (Fig. 5N-O). In contrast, WT or CDC42-overexpressing Paneth cells showed higher MitoTracker signals indicating higher numbers of functional mitochondria.

In addition to the above new experimental results linking CDC42-deficiency to abnormal mitochondrial ROS production, literature has also established an overall role of Cdc42 in regulating mitochondrial function and ROS production in various cell types (references cited across the manuscript introduction and discussion). Thus, the ROS regulation by CDC42 should be a much broader phenomenon rather than being Paneth cell-specific. These points have been added to discussion.

Interestingly, Cdc42 deletion reduced OLFM4+ stem cell numbers (Figure 7C). How does CDC42 control stem cells (via ROS and OXPHOS activity or not)? Did also the addition of reactive oxygen species to organoid culture also impair stem cells? If so, could this stem cell impairment rescued by modulating ROS and OXPHOS activity?

Response: As the Cdc42 deletion in original Fig. 7C was specifically in Paneth cells, we speculate that the reduced OLFM4 stem cell numbers in these mice was largely due to an indirect impact from the impaired Paneth cell function. Of note, Paneth cells constitute one of the stem cell niche supporting components. Based on Zhang et al., Cdc42-deficient stem cells do not renew or regenerate very well in response to gut epithelial injury (PMID: 32686657), but at this moment it is not clear whether the mechanism was through ROS/OXPHOS in stem cells. Rodriguez-Colman and colleagues have reported that physiological level of OXPHOS activity stimulates intestinal stem cell differentiation in enteroid culture (PMID: 28273069).

Referee #3:

The paper discusses interesting aspects of Paneth cells, which are under-represented in biomedical research, despite they are really essential. The paper is generally well written, and the figures are good quality. The story is of interest and this journal looks ideal for it to be published when certain aspects have been corrected and addressed.

1. Though I wrote that the paper is well written, there are a relatively high amount of language errors and the paper should be well edited. For example, in the abstract, there are some grammatical issues (two times a 'the' where inappropriate).

Response: Thanks for pointing out the grammatical issues, and we have now asked English native writers to proofread the paper.

2. The Paneth cell-specific cre mouse that was used looks like a great tool. Probably it was explained in the original 2023 paper, but also for this paper it is essential to know what fraction of site-specific recombination is found after TAM injection in the mice. Are all PCs showing TdT expression after TAM? And what about Cdc42 after TAM injection?

Response: In the original 2023 paper where this allele was first reported, we described that the CreER recombines in virtually all Paneth cells from the proximal to distal small intestines. The conclusion was drawn from examining tdT expression in lysozyme-expressing Paneth cells in numerous mice. The genomic DNA PCR analysis using primers spanning the two loxP sites that flank Cdc42 may arguably be the most sensitive method to pick up any recombined vs. un-recombined DNAs. This was illustrated in Fig. 5B, which shows that

approximately <5% Cdc42 flox alleles (pointed by the arrowheads) were not recombined in tdT^+ Paneth cells.

3. Also in relation to this crucial tool: The Lyz1 gene is strongly expressed in PCs. That is a fact, and thus the expression of Cre from that gene's promotor looks logical. However, Lyz1 is known to show ectopic expression in other cells (such as, but not limited to, Alveolar Macrophages, see PMID: 40636116). In this paper, it might not matter so much (although AMs might play a role in Salmonella survival), but it should be stated clearly.

Response: This point is well taken. Indeed, Lyz1 is expressed in some immune cells in addition to Paneth cells. Therefore, the Salmonella survival changes are not entirely due to the impact of the Paneth cells. The use of the Salmonella model was expected to trigger a pathological oxidative stress in the gut mucosal tissue then examine how the Paneth cells handle the oxidative response. We have now elaborated these points in Result and Discussion sections. Thank you!

4. There is crucial info lacking on how the PCs were isolated from mice. It looks like this is done via TdT and via FACS. As the authors will have read in the literature (PMID: 39273007), there is significant difference in PC transcriptomes in relation to the source of the PCs (duodenum, jejunum or ileum), so it has to be stated very clearly in results, figure legends and Materials where exactly the PCs were recruited and how many cells were obtained.

Response: We have added the requested information to necessary texts. We indeed used tamoxifen to induce tdT expression in Paneth cells, then isolated them by FACS sorting. If missing from the original legends, the source of PCs is now described in the legends.

5. I can see the point of the UMAPs in Fig.1, I am just wondering if the transcripts are the best ones to choose. I think the authors should add Lyz1 and TdT as positive controls, as well as Defa24 or Defa30, because these last ones are the strongest expressed out of the 28 Defa genes, to make sure all cells are really PCs, because I have my doubts given the previous point, and given the Fig1P and others, which may seem to suggest that some stem cells were co-sorted. A UMAP figure with data of Defa24 or Defa30 (or CD24) would be reassuring (Defa27 is one of the weakest Defa genes expressed in PCs (See PMID: 39981239).

Response: Thank you for the excellent point, and we have now added UMAPs for Lyz1 (Fig. 1E) and for Defa24, Defa30, CD24 (Expanded View Fig. 1B).

6. The fact that PCs, when maturing, have less TCA activity is well-known from the work of Riccardo Fodde. It is also considered that this is an essential step for PCs to produce lactate from pyruvate (and not send the pyruvate to the mitochondria, in order to sustain the stemness of these neighboring stem cells. The authors must consider this in their paper. Like everything in life, also for PCs, they must give and take something at the cost of something. Oxidative stress (as given here by hydrogen peroxide, very harsh) is a part of live and PCs use this to get their function done, maybe by the cost of some Defa expression, but honestly, this is the result of millions of years of evolution and seems a good thing.

Response: We are grateful for this important point about the metabolic response of Paneth cells to oxidative stress. To address this, we have now performed metabolomic analysis, using LC/MS, of sorted Paneth cells from enteroids that are stimulated by H_2O_2, FCCP, or PBS. The results show that ROS-treated Paneth cells have a significantly increased [lactate]/[pyruvate] (L:P) ratio (Fig. 2K). Due to the equilibrium of the reaction catalyzed by lactate dehydrogenase, the concentrations of the metabolites follow this equation: $[Pyr][NADH][H^+]=K_{eq}[Lac][NAD^+]$. This ratio reflects cellular energy metabolism, mitochondrial disorders, and the cellular redox state ($NADH:NAD^+$). A high L:P ratio suggests defects in the respiratory chain, which is consistent with the ROS treatment of these enteroids.

In addition, we performed stable isotope $^{13}C_6$-glucose tracing in enteroids of unchallenged vs. FCCP-treated conditions. The results suggest that most of $^{13}C_6$-glucose was used to make lactate in Paneth cells in this 17-hr pulse-chase experiment (Expanded View Fig. 2F-G), whereas no tracer was detected in TCA metabolites.

Furthermore, the Paneth cells possess overall higher concentrations of lactate than non-Paneth cells in our experiments (Expanded View Fig. 2E). We have incorporated these new results into the main and extended view figures.

7. Fig. 4B, this says 'probability to die'. What is this? Is this not a real survival curve? How is the probability calculated or so? Why is the survival curve stopped at Day5? Are there no further datapoints? Or did no further deaths occur? This issue about Day5 also applies to other curves in this paper. And Fig4C lacks a legend on the Y axis.

Response: We revised the labels to “Survival”. The reason for us to previously use ”probability of survival” is below: when the body weight of any given mouse dropped to 80% or more after infection, they were humanely sacrificed according to the animal protocol

approved by our institutional IACUC. These mice were counted as “death”. Thus, in these experiments, death was recorded as observed mortality as well as animals reaching the humane sacrifice endpoint. As we use infection to trigger the oxidative stress and would like to analyze the intestinal tissues from these infected mice, death was not intended as the investigative focus of this study. Therefore, we typically terminate the infection experiments at 5-6 d.p.i. so we can collect tissues from the animals for our study. The survival plot contained surviving mice by this experimental endpoint, which is not 100% mortality.

8. Figure 5, I have the same question as before: after TAM, if the PCs were sorted on TdT signal, of course only cells that showed cre activity are sorted, and will be cdc42 KO, which provides a beautiful PCR gel, but this is not correct to estimate the KO in all PCs of the mice after TAM. A classical sort via CD24 and c-Kit ligand should be done, or another technique, maybe RNAscope, to investigate the degree of Cdc42 in the IECs.

Response: As described in above point #2, the tdT reporter labels all $Lyz1^+$ Paneth cells in these reporter mice. The PCR was used to demonstrate the recombination efficacy at the Cdc42 gene locus, which showed a residual non-recombined flox allele (pointed by arrowheads, Fig. 5B). So, these results demonstrate Cdc42 recombination in the vast majority, but not all, Lyz1+ PCs. We do not expect the Lyz1-CreER will be able to delete Cdc42 in Lyz1-negative Paneth cells.

9. Fig. 5G: this is interesting, and should be confirmed with a Lyz1 staining and Defa24 (or 27) staining, perhaps via RNAscope. This is essential. Right now, the data suggest there is TdT expression via Cre activity, thus from Lyz1 promotor activity, but if this means that these signals are coming from new PCs developed should be confirmed, as written here, maybe also via TEM.

Response: We have added tdT co-staining with lysozyme, MUC2, and MMP7, and quantified all the results, to address the reviewer’s point (Fig. 6I-N, Expanded View Fig. 5C-E). We could not find a reliable commercial Defa24 (or 27) antibody. We also performed TEM analysis on WT and Cdc42ΔPC mice and quantified the results (Fig. 5G-K, Expanded View Fig. 5A-B).

10. Fig6B: this is a key figure, yet it is not optimal. It has low numbers of mice, and the experiment stops too soon. There is a * there, but the biological significance is very uncertain. Why are there no body weights here. This figure, along with the cdc42 VillKO rather suggest a very limited role of PCs in this story (the survival to Salmonella).

Response: We agree with the reviewer's view that Cdc42ΔIEC mice were much more susceptible than Cdc42ΔPC mice to the infection. As the reviewer pointed out VilKO will remove Cdc42 from all IECs including the enterocytes, intestinal stem cells, transit-amplifying cells as well as Paneth cells. In contrast, the Cdc42ΔPC mice will only reveal the impact from impaired PC biology. Thus, the Cdc42ΔPC mice were indeed less susceptible than the Cdc42ΔIEC mice. Based on the data and the reviewer's comments, we would not overstate the PCs' role in defending against Salmonella, rather these experiments allowed us to examine the oxidative responses in Cdc42ΔPC intestinal tissues.

11. The story lacks a convincing link between the cdc42 part and the possibility of animals or PCs to kill bacteria. One possibility is to purify PCs, lyse them and confront them with bacteria, as published by some groups. There should also be a look at MMP7 activity in this study, for obvious reasons.

Response: We now performed new MMP7 staining in infected WT and Cdc42ΔPC mice, and the data showed significantly reduced MMP7 expression in tdT^{+} cells lineage-traced in Cdc42ΔPC mice compared to the WT littermates (Expanded View Fig. 5C-E). Likewise, these tdT^{+} lineage traced PCs in Cdc42ΔPC mice also had reduced lysozyme (Fig. 6J, M-N). We appreciate the idea about PC's capability of killing bacteria (in this study: Salmonella Typhimurium SL1344). However, we have not been able to demonstrate, in our hands, such bactericidal effects by several PC-specific antimicrobial peptides including lysozyme in our recent publication (Han et al., 2024, JBC, PMID: 38823640). Thus, the animal's disease susceptibility to Salmonella infection involves more complicated innate and adaptive mucosal immune responses. We intended to use infection to model pathological oxidative stress, and we would not like to overstate the significance of PCs in killing the pathogen.

12. Fig.1P (and elsewhere): this is a picture, so what does it mean when it is written in the figure legend "Data represent 5 independent mice"

Response: We wanted to state that the staining results represented consistent observation from 5 independent mice. We appreciate the reviewer pointing the ambiguity and have now revised the text.

1st Revision - Editorial Decision 20th Jun 2026

Dear Nan,

Thank you for submitting a revised version of your manuscript to The EMBO journal. Your study has now been seen by two of the original referees, who find that their previous concerns have been sufficiently addressed and now recommend publication of the manuscript.

There now remain only a few editorial points that need to be addressed before I can extend the official acceptance of the manuscript:

1. Please note that it is The EMBO Journal policy for the transcript of the editorial process (containing referee reports and your response letters) to be published as an online supplement to each paper. If you should prefer removal of any referee-only figures included in the point-by-point response(s), e.g. because they may still be used for future publication or because they have been reproduced from published work by others, please do let us know immediately via response email.
More information is available here: https://www.embopress.org/transparent-process#Review_Process
2. The headings and order of the sections in the manuscript text should be as follows: Abstract / Introduction / Results / Discussion / Methods / Data Availability / Acknowledgements / Disclosure and Competing Interests Statement / References / Figure Legends / Expanded View Figure Legends.
3. CRediT has replaced the traditional author contributions section because it offers a systematic, machine-readable author contributions format that allows for more effective research assessment. Please remove the Authors Contributions from the manuscript and use the free text boxes beneath each contributing author's name in our online submission system to add specific details on the author's contribution. More information is available in our guide to authors.
4. In the Data Availability section, please add a resolvable link to the datasets. More information about the format of this section can be found here: https://link.springer.com/partners/embo-press/editorial-policies#Data%20availability%20statement.
5. Please rename expanded view figures into Figure EV1 - EV6 in the manuscript text, legends and file names.
6. Please remove the Reagents and Tools table from the manuscript and upload as a separate file it as a separate file choosing the file type "Reagent Table".
7. During our standard numerical data check, we noticed some numerical repetitions in the source data for figure 5H, tab "All" (please see in the attachment). Please take a look and correct if needed. A brief explanation would be very helpful - I appreciate that these duplications can also occur due to specific measurement or calculation methods used.
8. Our data editors have flagged the following issues in figure legends that need correcting:
- Please define the annotated p values **/***/**** as well as provide the exact p-values in the legend of figure 6I,J; EV-3F,G.
- Please provide the exact p values in the legends of 1Q; 3F; 4I,K,M; 5D,F,L,O; 6C,G; 7B,D,E,G; EV-2G,I; EV-4A,B,E,G; EV-5E; EV-6A-C.
- Please indicate the statistical test used for data analysis in the legends of figures 4E,F,M.
- Please note that the box plots need to be defined in terms of centre, bounds of box and percentile in the legends of figures 2D,M; 5L; EV-2I.
- Please define the box plots in terms of minima, maxima, centre, bounds of box and whiskers, and percentile in the legends of figure 3F.
- Please provide information on the number and nature of replicates in the legends 2K,M; 5O; 6I,J; EV-2E,G,I; EV-3F,G.
- Please provide the error bars are not defined in the legends of figure 2K; 4I,K; 5D,F,H,O; 6H,I,J; 7B,D,G; EV-2E,G; EV-3F,G; EV-4A,B,E,G.
- Please define the scale bar for figure EV-6D.
- Please note that the scale bar and its definition are missing for figures 1P; 5I,J,K; EV-1F.
- Please define the yellow asterisks in the legend of figure 5J.
- Please define the white arrowheads in the legend of figure 1R; 3C; 5E; EV-3B.
- Please define the yellow arrowheads in the legend of figures 5I,J,K; EV-3C.
- Please define the white dotted lines in the legend of figures 5N.
9. Papers published in The EMBO Journal are accompanied online by a 'Synopsis' to enhance discoverability of the manuscript. It consists of A) a short (1-2 sentences) summary of the findings and their significance, B) 3-4 bullet points highlighting key results (the highlights currently included in the manuscript can be used here) and C) a synopsis image that is 550x300 pixels large (width x height, jpeg or png format). You can either show a model or key data in the synopsis image. Please note that the image size is rather small and that text needs to be readable at the final size.

Please let me know if you have any questions regarding any of these points. You can use the link below to upload the revised files.

Thank you again for giving us the chance to consider your manuscript for The EMBO Journal. I look forward to receiving the final version.

With best wishes,

Ieva

Ieva Gailite, PhD
Senior Scientific Editor
The EMBO Journal
Meyerhofstrasse 1
D-69117 Heidelberg
Tel: +4962218891309
i.gailite@embojournal.org

We realize that it is difficult to revise to a specific deadline. In the interest of protecting the conceptual advance provided by the work, we recommend a revision within 3 months (18th Sep 2026). Please discuss the revision progress ahead of this time with the editor if you require more time to complete the revisions.

Referee #2:

The authors have addressed my suggestions, having improved the manuscript.

Referee #3:

The revised paper is greatly improved and of added value to the community and fit for the journal.

The authors addressed the remaining editorial issues.

3rd Revision - Editorial Decision 6th Jul 2026

Dear Nan,

Thank you for incorporating most of the final editorial and formatting requests in the manuscript. I am now pleased to inform you that your manuscript has been accepted for publication. Congratulations with a nice study!

Before we forward your manuscript to our publishers, there remain a couple of points that I need your input on before we can forward your manuscript to our publishers for typesetting:

1. Datasets GSE302951 and GSE301987 appear to be scheduled for public release on July 3, 2029. Please ensure that these datasets are publicly available at the proofs stage to ensure a smooth publication process.
2. We still require information on the nature of error bars in figure panels 4I, EV4A, G. Please send me this information via email, and we will add it in the manuscript for you.
3. We would like to propose some edits in the manuscript abstract and synopsis and have provided an alternative title suggestion to improve the accessibility of your study to our more general readership. I have also written a short blurb that will accompany the title of your manuscript in our online table of contents. Please take a look at the proposed text changes in the attached text file - any corrections or edits from your side are very welcome.

Once we have these points resolved, I will be happy to proceed with the acceptance of your study and its transfer for typesetting. Your manuscript will be processed for publication by EMBO Press. It will be copy edited and you will receive page proofs prior to publication. Please note that you will be contacted by Springer Nature Author Services to complete licensing and payment information.

You may qualify for financial assistance for your publication charges - either via a Springer Nature fully open access agreement or an EMBO initiative. Check your eligibility: https://link.springer.com/journal/44318/how-to-publish-with-us

Should you be planning a Press Release on your article, please notify the production team via this form in order to coordinate publication and release dates: https://forms.cloud.microsoft/e/eH4A8JVP6g

If you have any questions, please do not hesitate to contact the Editorial Office. Thank you for this interesting contribution to The EMBO Journal!

i look forward to hearing from you soon.

With best wishes,

Ieva

Ieva Gailite, PhD
Senior Scientific Editor
The EMBO Journal
Meyerhofstrasse 1
69117 Heidelberg
i.gailite@embojournal.org

EMBO Press Author Checklist

Corresponding Author Name: Nan Gao	
Journal Submitted to: EMBO J	
Manuscript Number: EMBOJ-2025-122894	

USEFUL LINKS FOR COMPLETING THIS FORM
The EMBO Journal - Author Guidelines
EMBO Reports - Author Guidelines
Molecular Systems Biology - Author Guidelines
EMBO Molecular Medicine - Author Guidelines

Reporting Checklist for Life Science Articles (updated January
This checklist is adapted from Materials Design Analysis Reporting (MDAR) Checklist for Authors. MDAR establishes a minimum set of requirements in transparent reporting in the life sciences (see Statement of Task: 10.31222/osf.io/9sm4x). Please follow the journal's guidelines in preparing your
Please note that a copy of this checklist will be published alongside your article.

Abridged guidelines for figures
1. Data
The data shown in figures should satisfy the following conditions:
- the data were obtained and processed according to the field's best practice and are presented to reflect the results of the experiments in an accurate and unbiased manner.
- ideally, figure panels should include only measurements that are directly comparable to each other and obtained with the same assay.
- plots include clearly labeled error bars for independent experiments and sample sizes. Unless justified, error bars should not be shown for technical
- if n<5, the individual data points from each experiment should be plotted. Any statistical test employed should be justified.
- Source Data should be included to report the data underlying figures according to the guidelines set out in the authorship guidelines on Data

2. Captions
Each figure caption should contain the following information, for each panel where they are relevant:
- a specification of the experimental system investigated (eg cell line, species name).
- the assay(s) and method(s) used to carry out the reported observations and measurements.
- an explicit mention of the biological and chemical entity(ies) that are being measured.
- an explicit mention of the biological and chemical entity(ies) that are altered/varied/perturbed in a controlled manner.
- the exact sample size (n) for each experimental group/condition, given as a number, not a range;
- a description of the sample collection allowing the reader to understand whether the samples represent technical or biological replicates (including how many animals, litters, cultures, etc.).
- a statement of how many times the experiment shown was independently replicated in the laboratory.
- definitions of statistical methods and measures:
  - common tests, such as t-test (please specify whether paired vs. unpaired), simple $\chi 2$ tests, Wilcoxon and Mann-Whitney tests, can be unambiguously identified by name only, but more complex techniques should be described in the methods section;
  - are tests one-sided or two-sided?
  - are there adjustments for multiple comparisons?
  - exact statistical test results, e.g., P values = x but not P values < x;
  - definition of 'center values' as median or average;
  - definition of error bars as s.d. or s.e.m.

Please complete ALL of the questions below.
Select "Not Applicable" only when the requested information is not relevant for your study.

Materials

Newly Created Materials	Information included in the manuscript?	In which section is the information available? (Reagents and Tools Table, Materials and Methods, Figures, Data Availability Section)
New materials and reagents need to be available; do any restrictions apply?	Yes	MATERIAL AND METHOD

Antibodies	Information included in the manuscript?	In which section is the information available? (Reagents and Tools Table, Materials and Methods, Figures, Data Availability Section)
For **antibodies** provide the following information:<br>- Commercial antibodies: RRID (if possible) or supplier name, catalogue number and or/clone number<br>- Non-commercial: RRID or citation	Yes	MATERIAL AND METHOD

DNA and RNA sequences	Information included in the manuscript?	In which section is the information available? (Reagents and Tools Table, Materials and Methods, Figures, Data Availability Section)
**Short novel DNA or RNA including primers, probes**: provide the sequences.	Yes	MATERIAL AND METHOD

Cell materials	Information included in the manuscript?	In which section is the information available? (Reagents and Tools Table, Materials and Methods, Figures, Data Availability Section)
**Cell lines:** Provide species information, strain. Provide accession number in repository **OR** supplier name, catalog number, clone number, and/**OR** RRID.	Not Applicable	
**Primary cultures:** Provide species, strain, sex of origin, genetic modification status.	Yes	MATERIAL AND METHOD
Report if the cell lines were recently **authenticated** (e.g., by STR profiling) and tested for mycoplasma contamination.	Not Applicable	

Experimental animals	Information included in the manuscript?	In which section is the information available? (Reagents and Tools Table, Materials and Methods, Figures, Data Availability Section)
**Laboratory animals or Model organisms:** Provide species, strain, sex, age, genetic modification status. Provide accession number in repository **OR** supplier name, catalog number, clone number, **OR** RRID.	Yes	MATERIAL AND METHOD
**Animal observed in or captured from the field:** Provide species, sex, and age where possible.	Yes	MATERIAL AND METHOD
Please detail **housing and husbandry conditions**.	Yes	MATERIAL AND METHOD

Plants and microbes	Information included in the manuscript?	In which section is the information available? (Reagents and Tools Table, Materials and Methods, Figures, Data Availability Section)
**Plants:** provide species and strain, ecotype and cultivar where relevant, unique accession number if available, and source (including location for collected wild specimens).	Not Applicable	
**Microbes:** provide species and strain, unique accession number if available, and source.	Yes	MATERIAL AND METHOD

Human research participants	Information included in the manuscript?	In which section is the information available? (Reagents and Tools Table, Materials and Methods, Figures, Data Availability Section)
If collected and within the bounds of privacy constraints report on age, sex and gender or ethnicity for all study participants.	Not Applicable	

Core facilities	Information included in the manuscript?	In which section is the information available? (Reagents and Tools Table, Materials and Methods, Figures, Data Availability Section)
If your work benefited from core facilities, was their service mentioned in the acknowledgments section?	Yes	ACKNOWLEDGMENTS

Design

Study protocol	Information included in the manuscript?	In which section is the information available? (Reagents and Tools Table, Materials and Methods, Figures, Data Availability Section)
If study protocol has been **pre-registered, provide DOI in the manuscript**. For clinical trials, provide the trial registration number **OR** cite DOI.	Not Applicable	
Report the **clinical trial registration number** (at ClinicalTrials.gov or equivalent), where applicable.	Not Applicable	

Laboratory protocol	Information included in the manuscript?	In which section is the information available? (Reagents and Tools Table, Materials and Methods, Figures, Data Availability Section)
Provide DOI OR other citation details if **external detailed step-by-step protocols** are available.	Yes	MATERIAL AND METHOD

Experimental study design and statistics	Information included in the manuscript?	In which section is the information available? (Reagents and Tools Table, Materials and Methods, Figures, Data Availability Section)
Include a statement about **sample size** estimate even if no statistical methods were used.	Yes	FIGURE LEGEND
Were any steps taken to minimize the effects of subjective bias when allocating animals/samples to treatment (e.g. **randomization procedure**)? If yes, have they been described?	Yes	MATERIAL AND METHOD
Include a statement about **blinding** even if no blinding was done.	Yes	MATERIAL AND METHOD
Describe **inclusion/exclusion criteria** if samples or animals were excluded from the analysis. Were the criteria pre-established?<br><br>If sample or data points were omitted from analysis, report if this was due to attrition or intentional exclusion and provide justification.	Not Applicable	
For every figure, are **statistical tests** justified as appropriate? Do the data meet the assumptions of the tests (e.g., normal distribution)? Describe any methods used to assess it. Is there an estimate of variation within each group of data? Is the variance similar between the groups that are being statistically compared?	Yes	FIGURE LEGEND

Sample definition and in-laboratory replication	Information included in the manuscript?	In which section is the information available? (Reagents and Tools Table, Materials and Methods, Figures, Data Availability Section)
In the figure legends: state number of times the experiment was **replicated** in laboratory.	Yes	FIGURE LEGEND
In the figure legends: define whether data describe **technical or biological replicates**.	Yes	FIGURE LEGEND

Ethics

Ethics	Information included in the manuscript?	In which section is the information available? (Reagents and Tools Table, Materials and Methods, Figures, Data Availability Section)
Studies involving **human participants**: State details of **authority granting ethics approval** (IRB or equivalent committee(s), provide reference number for approval.	Not Applicable	
Studies involving **human participants**: Include a statement confirming that **informed consent** was obtained from all subjects and that the experiments conformed to the principles set out in the WMA Declaration of Helsinki and the Department of Health and Human Services Belmont Report.	Not Applicable	
Studies involving **human participants:** For publication of **patient photos**, include a statement confirming that consent to publish was obtained.	Not Applicable	
Studies involving experimental **animals**: State details of **authority granting ethics approval** (IRB or equivalent committee(s), provide reference number for approval. Include a statement of compliance with ethical regulations.	Yes	MATERIAL AND METHODS, MICE
Studies involving **specimen and field samples:** State if relevant **permits** obtained, provide details of authority approving study; if none were required, explain why.	Not Applicable	

Dual Use Research of Concern (DURC)	Information included in the manuscript?	In which section is the information available? (Reagents and Tools Table, Materials and Methods, Figures, Data Availability Section)
Could your study fall under dual use research restrictions? Please check biosecurity documents and list of **select agents and toxins** (CDC): https://www.selectagents.gov/sat/list.htm	Not Applicable	
If you used a select agent, is the security level of the lab appropriate and reported in the manuscript?	Not Applicable	
If a study is subject to dual use research of concern regulations, is the name of the **authority granting approval and reference number** for the regulatory approval provided in the manuscript?	Not Applicable	

Reporting

The MDAR framework recommends adoption of discipline-specific guidelines, established and endorsed through community initiatives. Journals have their own policy about requiring specific guidelines and recommendations to complement MDAR.

Adherence to community standards	Information included in the manuscript?	In which section is the information available? (Reagents and Tools Table, Materials and Methods, Figures, Data Availability Section)
State if relevant guidelines or checklists (e.g., **ICMJE, MIBBI, ARRIVE, PRISMA**) have been followed or provided.	Not Applicable	
For **tumor marker prognostic studies,** we recommend that you follow the **REMARK** reporting guidelines (see link list at top right). See author guidelines, under 'Reporting Guidelines'. Please confirm you have followed these guidelines.	Not Applicable	
For **phase II and III randomized controlled trials**, please refer to the **CONSORT** flow diagram (see link list at top right) and submit the CONSORT checklist (see link list at top right) with your submission. See author guidelines, under 'Reporting Guidelines'. Please confirm you have submitted this list.	Not Applicable	

Data Availability

Data availability	Information included in the manuscript?	In which section is the information available? (Reagents and Tools Table, Materials and Methods, Figures, Data Availability Section)
Have **primary datasets** been deposited according to the journal's guidelines (see 'Data Deposition' section) and the respective accession numbers provided in the Data Availability Section?	Yes	DATA AVAILABILITY SECTION
Were **human clinical and genomic datasets** deposited in a public access-controlled repository in accordance to ethical obligations to the patients and to the applicable consent agreement?	Not Applicable	
Are **computational models** that are central and integral to a study available without restrictions in a machine-readable form? Were the relevant accession numbers or links provided?	Not Applicable	
If publicly available data were reused, provide the respective **data citations in the reference list**.	Yes	DATA AVAILABILITY SECTION
