# Supplementary material for: Reactive oxygen species stimulate Paneth cell plasticity and diminish antimicrobial function
Source: EMBO J. 2026 Jul 18;45(19):6723–58. doi: 10.1038/s44318-026-00869-y (PMC13631327; doi:10.1038/s44318-026-00869-y)
Supplement: Supplementary file 2 [file 44318_2026_869_MOESM9_ESM.pdf]

Expanded View Figures

Figure EV1. Intracellular ROS levels vary across Paneth cell populations.

(**A**) CytoTRACE potency was calculated for individual Paneth cells. (**B–D**) UMAP for Cd24a, Defa24, and Defa30. (**E**) Live intestinal epithelial cells were resolved by the expression of tdT and CD24. (**F**) Imagestream analysis for CellROX was performed for tdT^{High} and tdT^{Low} Paneth cells, $tdT^{-}CD24^{+}$ EECs and $tdT^{-}CD24^{-}$ IECs. Scale bar, 10 μm.

 © The Author(s)

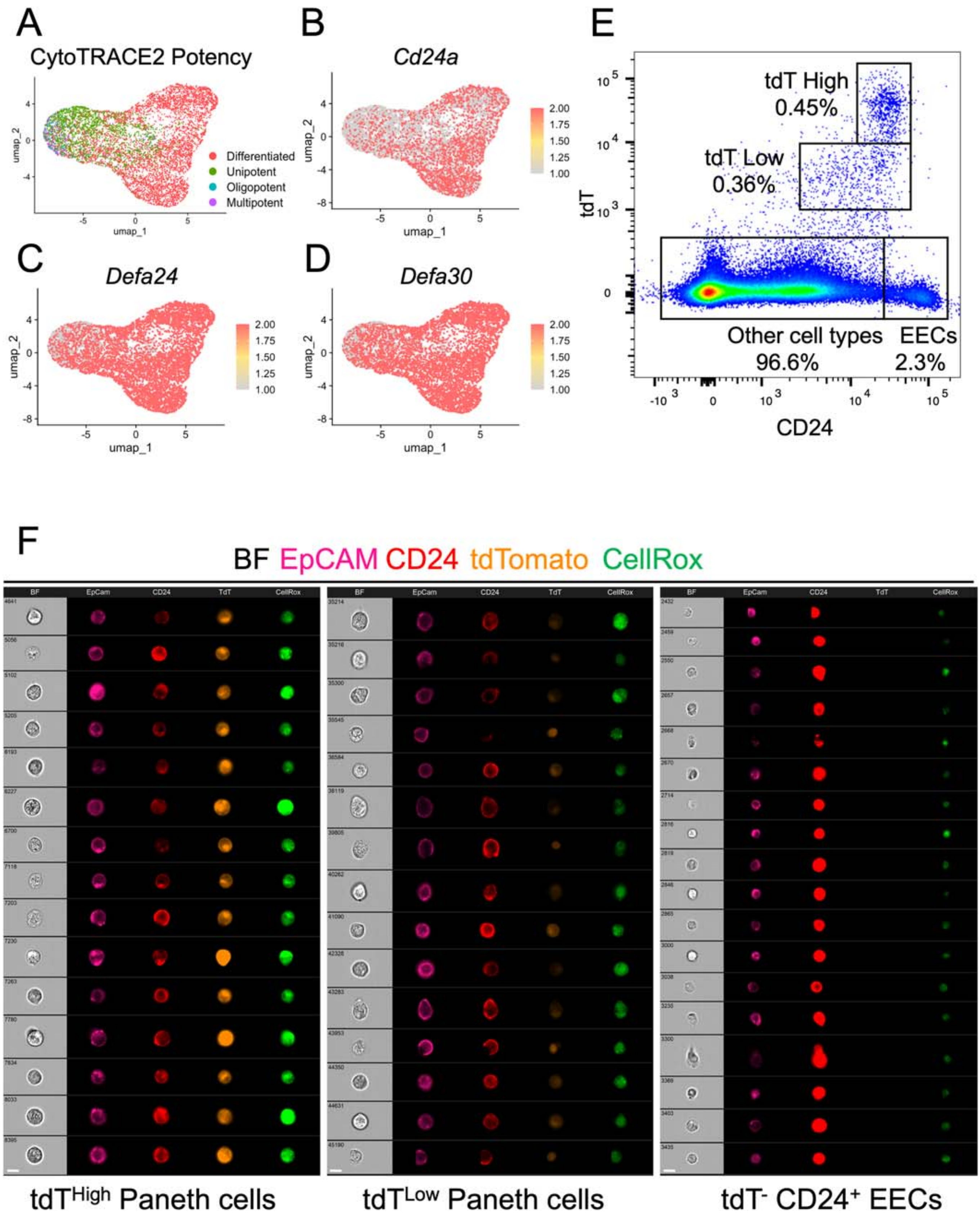
A
CytoTRACE2 Potency
Differentiated
Unipotent
Oligopotent
Multipotent
umap_1
umap_2
B
Cd24a
C
Defa24
D
Defa30
E
tdT High
0.45%
tdT Low
0.36%
Other cell types
96.6%
EECs
2.3%
tdT
CD24
F
BF EpCAM CD24 tdTomato CellRox
tdT^High Paneth cells
tdT^Low Paneth cells
tdT⁻ CD24⁺ EECs

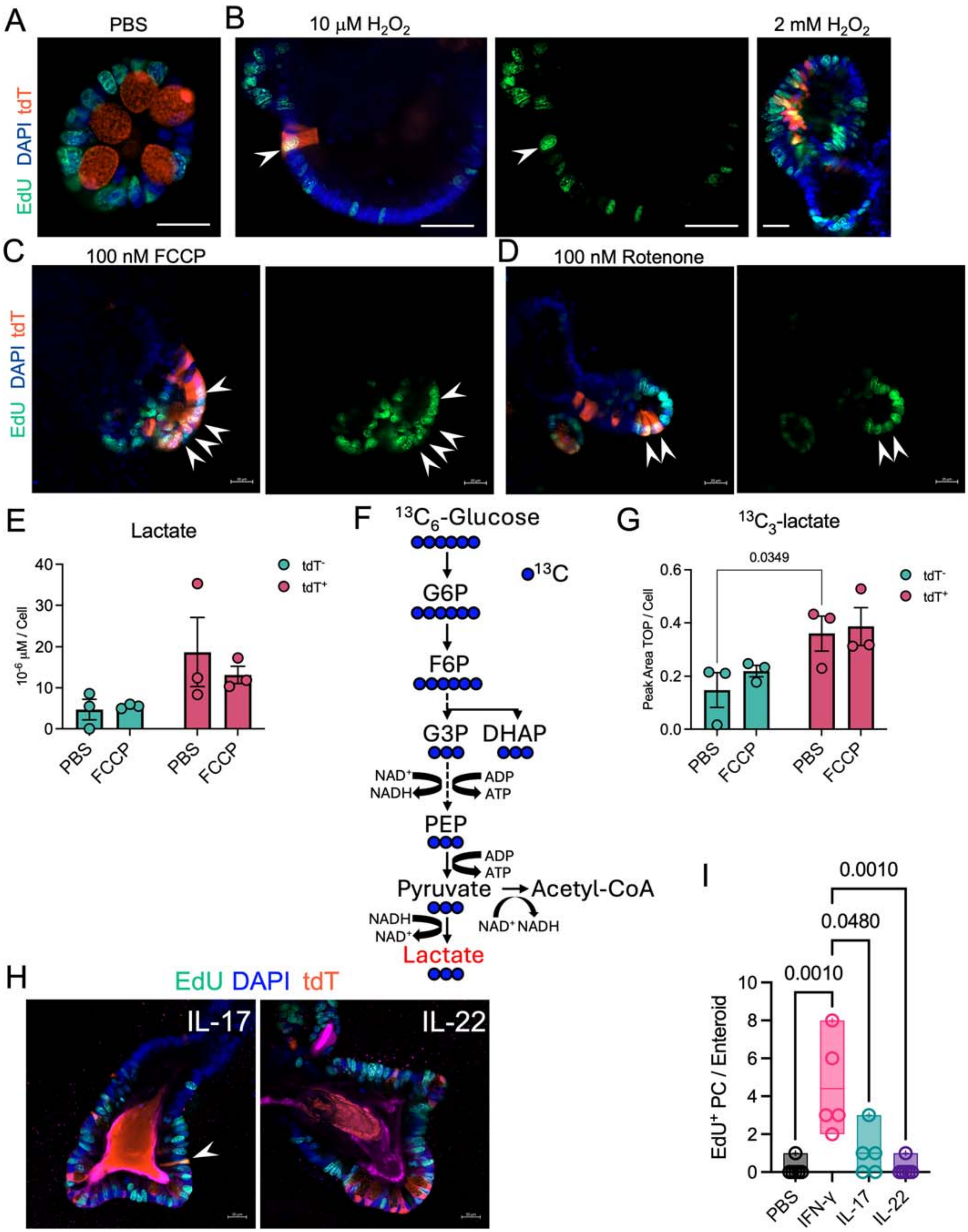
A
PBS
EdU DAPI tdT
B
10 μM H2O2
2 mM H2O2
C
100 nM FCCP
EdU DAPI tdT
D
100 nM Rotenone
E
Lactate
10-6 μM / Cell
tdT-
tdT+
PBS
FCCP
F
13C6-Glucose
13C
G6P
F6P
G3P
DHAP
NAD+
NADH
ADP
ATP
PEP
Pyruvate
Acetyl-CoA
NAD+ NADH
Lactate
G
13C3-lactate
0.0349
Peak Area TOP / Cell
H
EdU DAPI tdT
IL-17
IL-22
I
0.0010
0.0480
EdU+ PC / Enteroid
PBS
IFN-γ
IL-17
IL-22

Figure EV2. Impact of oxidative stress on cell-cycle entry and metabolism in Paneth cells.

(**A–D**) Enteroids were grown from PC reporter mouse duodenum, induced for tdT expression on day 3, and treated with various ROS for 24 h. Enteroids were labeled with EdU for 1 h before fixation and imaging. Scale bar, 20 µm. Arrowheads point to EdU^+tdT^+ cells. Scale bar, 20 µm. (**E**) Approximately 1000 duodenal enteroids from PC reporter mice ($n = 3$) were treated with FCCP for 24 h, washed out, and 17.5 mM $^{13}C_6$-glucose was added to the medium for a duration of 17 h before washing out and proceeding to FACS sorting. tdT^+ and tdT^- IECs were isolated for LC-MS analysis. Lactate concentrations were normalized against the cell number of individual samples and plotted. Data are presented as mean ± SEM. (**F**) Schematic diagram illustrating the glycolysis of glucose to pyruvate, which is either converted to lactate through anaerobic respiration or enters mitochondria to fuel the TCA cycle. (**G**) Using samples from the experiment described in (**E**), $^{13}C_3$-lactate abundance was quantified from individual samples treated with PBS or FCCP. A two-way ANOVA followed by Fisher's LSD test was performed to compare the difference. Exact *p*-value is provided above the comparison bar. Data are presented as mean ± SEM. (**H**) Enteroids were grown from PC reporter mouse duodenum, induced for tdT expression on day 3, and treated with 50 ng/mL IL-17 or 4 ng/mL IL-22 for 24 h. Enteroids were labeled with EdU for 1 h before fixation and imaging. Scale bar, 20 µm. Arrowheads point to an EdU^+tdT^+ cell in the IL-17-treated condition. (**I**) The number of EdU^+tdT^+ cells was calculated for individual enteroids treated with PBS ($n = 6$), IFN-γ ($n = 5$), IL-17 ($n = 6$), or IL-22 ($n = 5$). Statistical comparisons between groups were performed using a Kruskal-Wallis test followed by an uncorrected Dunn's multiple comparisons test. Exact *p*-values are provided above the comparison bars. Data are presented as floating bars (min to max) with the central line indicating the mean.

© The Author(s)

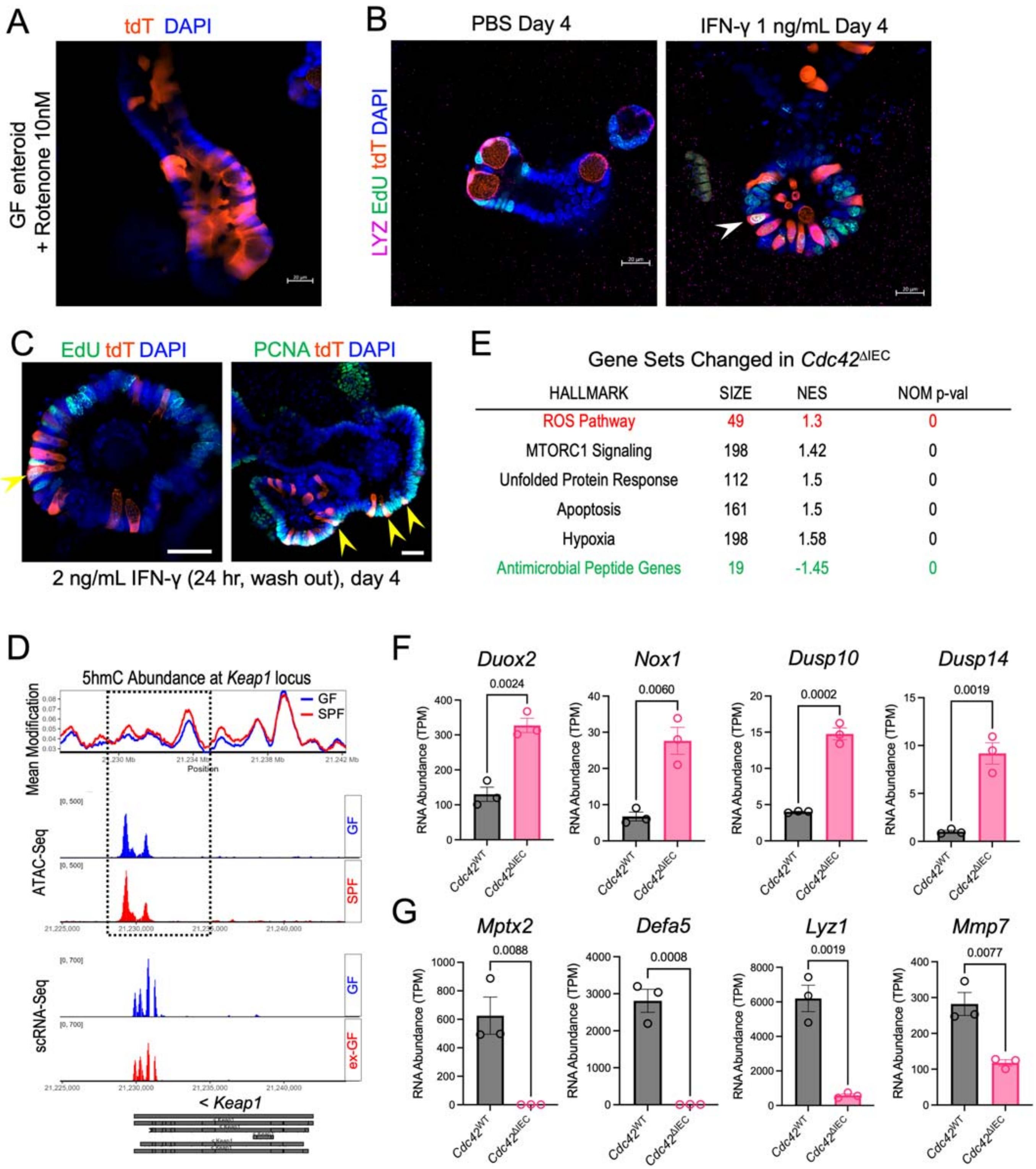
A
tdT DAPI
GF enteroid + Rotenone 10nM
B
PBS Day 4
IFN-γ 1 ng/mL Day 4
LYZ EdU tdT DAPI
C
EdU tdT DAPI
PCNA tdT DAPI
2 ng/mL IFN-γ (24 hr, wash out), day 4
E
Gene Sets Changed in Cdc42ΔIEC
HALLMARK SIZE NES NOM p-val
ROS Pathway 49 1.3 0
MTORC1 Signaling 198 1.42 0
Unfolded Protein Response 112 1.5 0
Apoptosis 161 1.5 0
Hypoxia 198 1.58 0
Antimicrobial Peptide Genes 19 -1.45 0
D
5hmC Abundance at Keap1 locus
Mean Modification
GF
SPF
Position
ATAC-Seq
scRNA-Seq
ex-GF
< Keap1
F
Duox2
0.0024
Nox1
0.0060
Dusp10
0.0002
Dusp14
0.0019
RNA Abundance (TPM)
Cdc42WT
Cdc42ΔIEC
G
Mptx2
0.0088
Defa5
0.0008
Lyz1
0.0019
Mmp7
0.0077

 © The Author(s)

Figure EV3. *Cdc42* deficiency upregulates the ROS pathway and suppresses the AMP gene program.

(**A**) Enteroids were grown from GF Paneth cell reporter mice. On day 3, 4-OHT was added, and on the following day enteroids were treated with 10 nM rotenone. The image was taken of an enteroid that showed a tdT$^+$ stripe. Scale bar, 20 µm. (**B**) Enteroids from Paneth cell reporter mouse duodenum were continuously treated with 1 ng/mL IFN-γ or PBS for 4 days, labeled with EdU 1 h before fixation, and stained for lysozyme, EdU, tdT, and DAPI. The white arrow indicates a LYZ$^+$EdU$^+$tdT$^+$ cell. Scale bar, 20 µm. (**C**) Enteroids from Paneth cell reporter mouse duodenum were treated with 2 ng/mL IFN-γ for 24 h, washed out, then cultured to day 4, labeled with EdU for 1 h, fixed and stained for lysozyme, EdU, tdT, and DAPI; or PCNA, tdT, and DAPI. Yellow arrows indicate PCNA$^+$tdT$^+$ cells. Scale bar, 20 µm. (**D**) Paneth cell specific multi-modality omics for 5hmC DNA nanopore-sequencing, ATAC-seq and scRNA-seq of Paneth cells from GF and SPF PC reporter mice. (**E**) Bulk RNA-seq was performed using WT vs. *Cdc42*$^{\Delta IEC}$ mouse small intestinal epithelia ($n = 3$ per condition). Hallmark gene sets for reactive oxygen species (red) and antimicrobial peptides (green) are significantly increased and decreased in *Cdc42*$^{\Delta IEC}$ mice, respectively. (**F**) RNA abundances of ROS production and regulatory enzymes in *Cdc42*^{WT} ($n = 3$) and *Cdc42*$^{\Delta IEC}$ ($n = 3$) mice are shown. A two-tailed Student's *t*-test was used to compare the differences. Exact *p*-values are provided above the comparison bars. Data are presented as mean ± SEM. (**G**) RNA abundances of AMP genes in *Cdc42*^{WT} ($n = 3$) and *Cdc42*$^{\Delta IEC}$ ($n = 3$) mice. A two-tailed Student's *t*-test was used to compare the differences. Exact *p*-values are provided above the comparison bars. Data are presented as mean ± SEM.

© The Author(s)

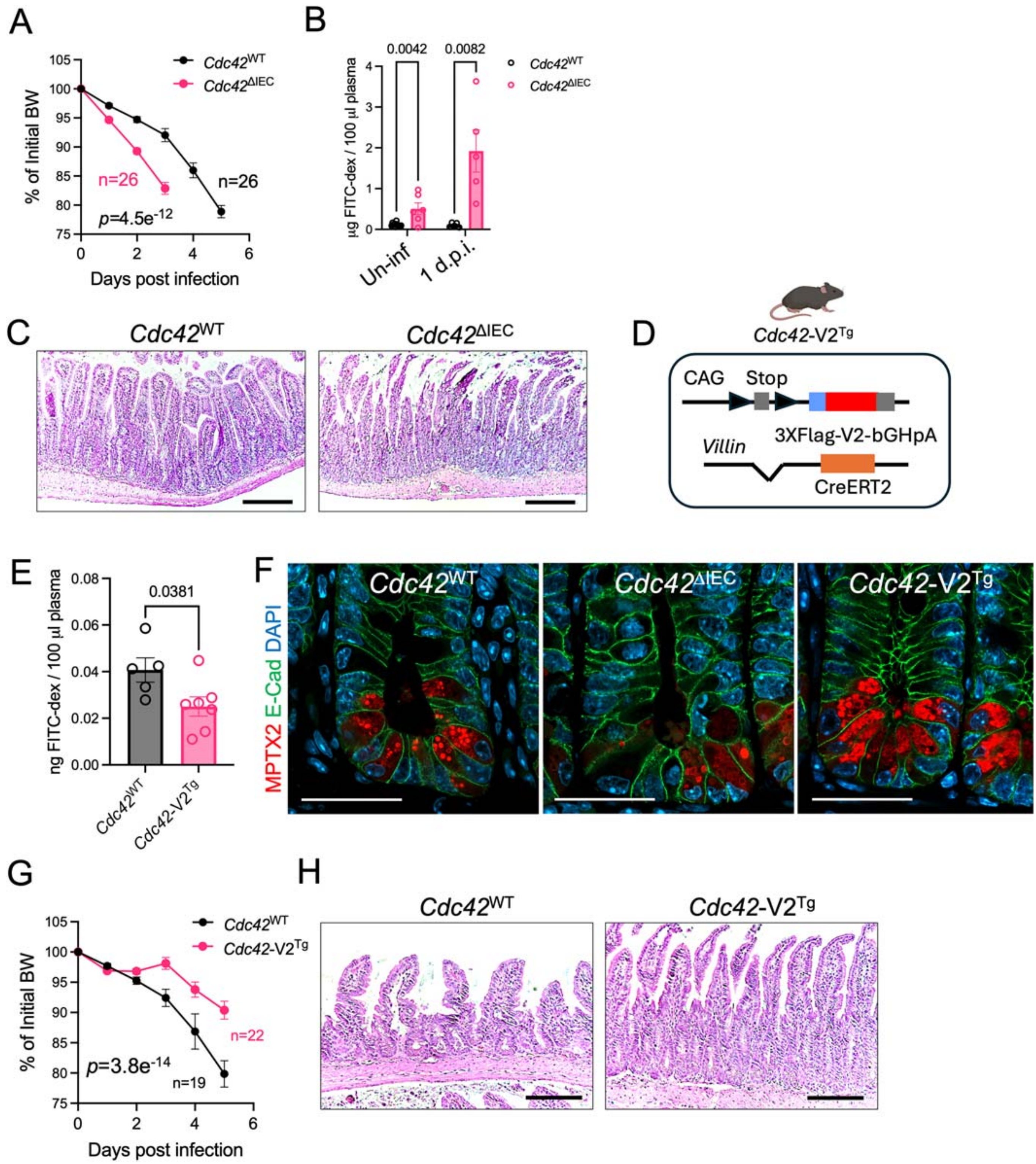
A
% of Initial BW
Cdc42WT
Cdc42ΔIEC
n=26
n=26
p=4.5e-12
Days post infection
B
μg FITC-dex / 100 μl plasma
0.0042
0.0082
Cdc42WT
Cdc42ΔIEC
Un-inf
1 d.p.i.
C
Cdc42WT
Cdc42ΔIEC
D
Cdc42-V2Tg
CAG
Stop
3XFlag-V2-bGHpA
Villin
CreERT2
E
ng FITC-dex / 100 μl plasma
0.0381
Cdc42WT
Cdc42-V2Tg
F
MPTX2 E-Cad DAPI
Cdc42WT
Cdc42ΔIEC
Cdc42-V2Tg
G
% of Initial BW
Cdc42WT
Cdc42-V2Tg
n=22
p=3.8e-14
n=19
Days post infection
H
Cdc42WT
Cdc42-V2Tg

 © The Author(s)

Figure EV4. Cdc42 deficiency increases susceptibility to *S. Typhimurium* infection.

(**A**) Body weight changes of *Cdc42*^{WT} and *Cdc42*$^{\Delta IEC}$ littermates following the *S. Typhimurium* oral inoculation. $n = 26$ for each condition. Curves were modeled using exponential (Malthusian) growth regression. Curves were compared using the Extra sum-of-squares F test. Data are presented as mean ± SEM. (**B**) An in vivo gut permeability assay was performed before the mice were sacrificed. Two hours after oral dosing of FITC-conjugated 4 kDa dextran, nanograms of dextran circulating in the plasma of uninfected mice and mice infected for one day were detected, for *Cdc42*^{WT} and *Cdc42*$^{\Delta IEC}$ mice. Statistical comparisons were performed using multiple unpaired *t*-tests with the two-stage step-up method of Benjamini, Krieger, and Yekutieli. Exact *p*-values are provided above the comparison bars. Data are presented as mean ± SEM. (**C**) Histology of infected *Cdc42*^{WT} and *Cdc42*$^{\Delta IEC}$ mouse ileum at 1 d.p.i. Scale bar, 100 µm. (**D**) Schematic diagram showing that *Cdc42*-V2^{Tg}; Villin-CreER^{T2} mice overexpress the CDC42 isoform. (**E**) Mice were orally gavaged with FITC-conjugated 4 kDa dextran 2 h before sacrifice. Nanograms of fluorescent dextran were measured in plasma of *Cdc42*^{WT} ($n = 5$) and *Cdc42*-V2^{Tg} ($n = 7$) mice. A Student's *t*-test was used to compare the difference. Exact *p*-value is provided above the comparison bar. Data are presented as mean ± SEM. (**F**) Immunofluorescent staining was performed for E-Cad and MPTX2 in *Cdc42*^{WT}, *Cdc42*$^{\Delta IEC}$, and *Cdc42*-V2^{Tg} mice. Scale bar, 50 µm. (**G**) Body weights were measured from infected *Cdc42*-V2^{Tg} ($n = 22$) and *Cdc42*^{WT} ($n = 19$) littermates. Curves were modeled using exponential (Malthusian) growth regression. Curves were compared using the Extra sum-of-squares F test. Data are presented as mean ± SEM. (**H**) Histology of infected *Cdc42*^{WT} and *Cdc42*-V2^{Tg} mouse ileum. Scale bar, 100 µm.

© The Author(s)

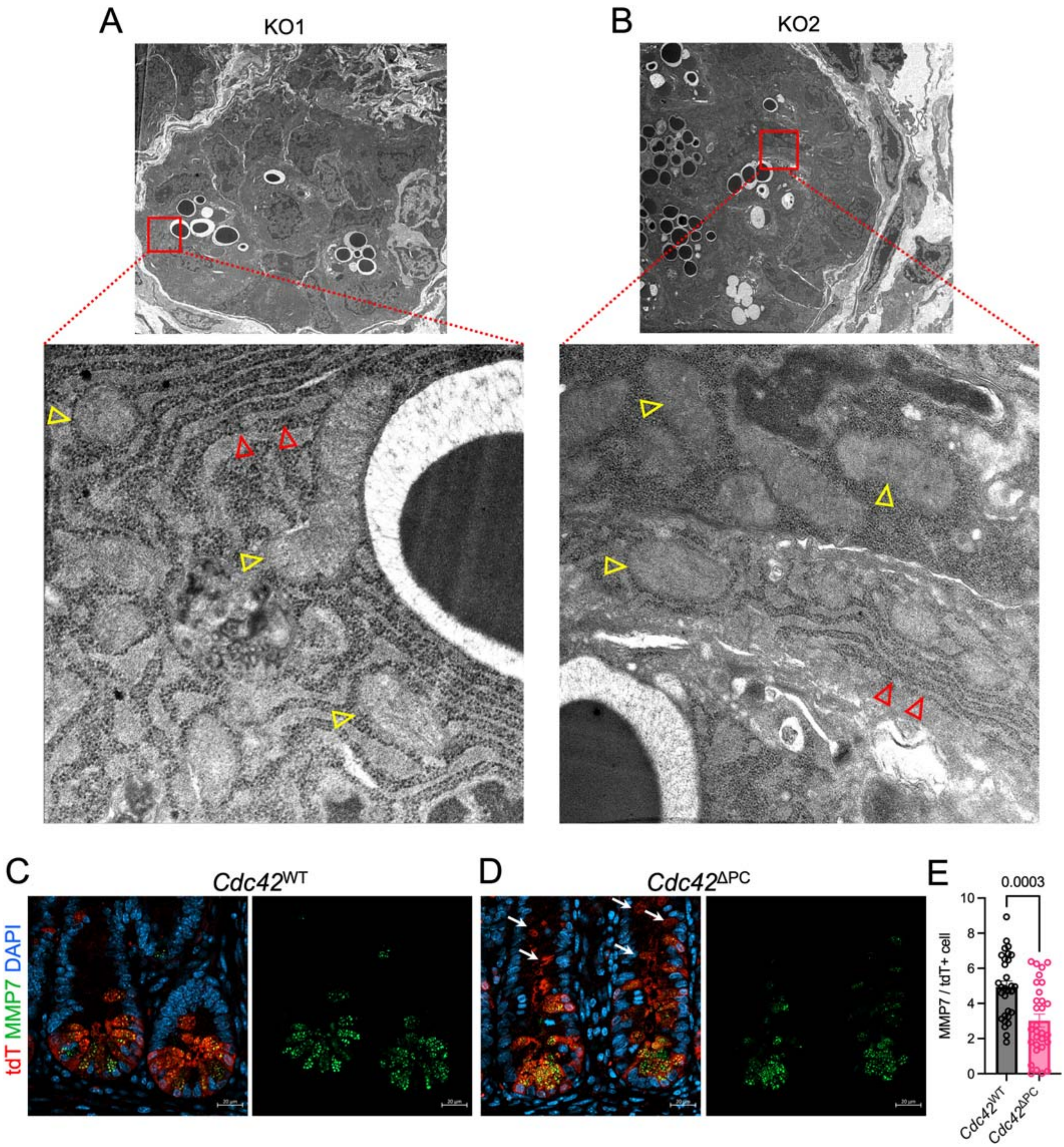

Figure EV5. PC-specific deletion of *Cdc42* impairs mitochondrial integrity and MMP7 production.

(**A**, **B**) TEM micrographs of *Cdc42*$^{\Delta PC}$ mouse ilea. These representative images illustrate the severe mitochondrial cristolysis (yellow arrows) and pronounced ER lumen dilation (red arrows) in virtually all the PCs of *Cdc42*$^{\Delta PC}$ mice. (**C, D**) Confocal immunofluorescence analysis of MMP7 and tdT in *S. Typhimurium* infected *Cdc42*^{WT} and *Cdc42*$^{\Delta PC}$ mice. Arrows in panel F point to up-migrating tdT$^+$ cells that do not express MMP7. (**E**) MMP7 abundances in individual tdT$^+$ cells were quantified from *Cdc42*^{WT} and *Cdc42*$^{\Delta PC}$ mice, and statistically compared by a Student's *t*-test. Exact *p*-value is provided above the comparison bar. Data are presented as mean ± SEM.

 © The Author(s)

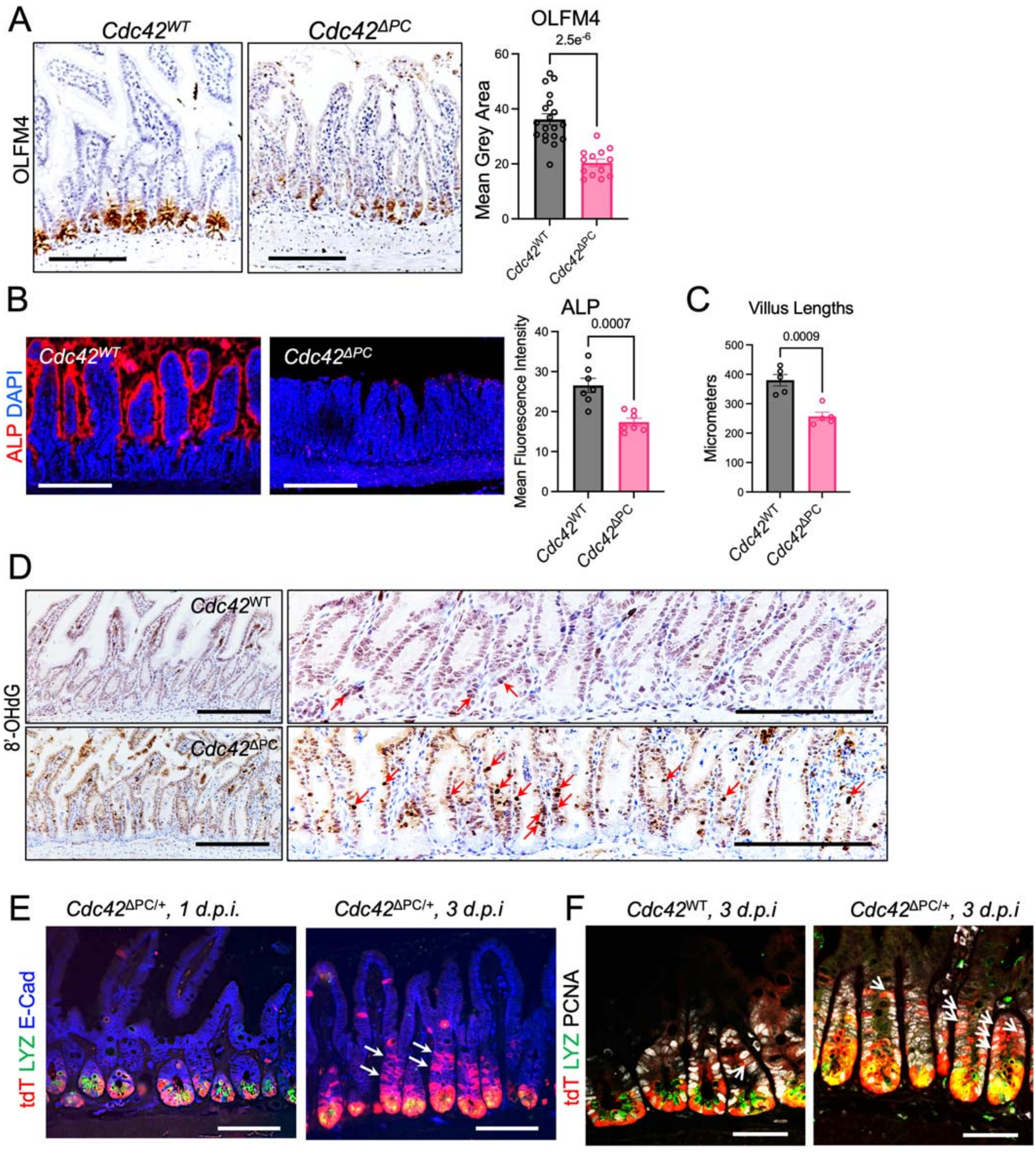
A
Cdc42WT
Cdc42ΔPC
OLFM4
OLFM4
2.5e-6
Mean Grey Area
60
40
20
0
Cdc42WT
Cdc42ΔPC
B
Cdc42WT
Cdc42ΔPC
ALP DAPI
ALP
0.0007
Mean Fluorescence Intensity
40
30
20
10
0
C
Villus Lengths
0.0009
Micrometers
500
400
300
200
100
0
D
8'-OHdG
E
Cdc42ΔPC/+, 1 d.p.i.
Cdc42ΔPC/+, 3 d.p.i
tdT LYZ E-Cad
F
Cdc42WT, 3 d.p.i
Cdc42ΔPC/+, 3 d.p.i
tdT LYZ PCNA

Figure EV6. PC-specific *Cdc42* knockout reduces stem cell and enterocyte populations and elevates DNA oxidation during *S. Typhimurium* infection.

(**A**) Immunostaining was performed for OLFM4 in infected $Cdc42^{WT}$ and $Cdc42^{\Delta PC}$ mouse intestines. A Student's *t*-test was performed to compare OLFM4 abundance in crypts between the two groups. Exact *p*-value is provided above the comparison bar. Scale bar, 100 µm. Data are presented as mean ± SEM. (**B**) Immunostaining was performed for alkaline phosphatase (ALP) in infected $Cdc42^{WT}$ and $Cdc42^{\Delta PC}$ intestines. Scale bar, 100 µm. A Student's *t*-test was performed to compare the ALP between the two groups. Exact *p*-value is provided above the comparison bar. Data are presented as mean ± SEM. (**C**) Villus lengths were measured from the infected $Cdc42^{WT}$ and $Cdc42^{\Delta PC}$ mice at 4 d.p.i. and analyzed by a Student's *t*-test. Exact *p*-value is provided above the comparison bar. Data are presented as mean ± SEM. (**D**) Immunostaining for 8′-OHdG using infected $Cdc42^{WT}$ and $Cdc42^{\Delta PC}$ intestinal sections. Red arrows point to 8′-OHdG puncta. Scale bar, 100 µm. (**E**) Tamoxifen was injected into $Cdc42^{\Delta PC/+}$ mice carrying a PC reporter. Mice were infected with *S. Typhimurium and* sacrificed at 1 and 3 d.p.i. Sections were stained for tdT and lysozyme. Arrows point to up-migrating tdT^+ cells. Scale bar, 50 µm. (**F**) Tamoxifen was injected into $Cdc42^{WT}$ and $Cdc42^{\Delta PC/+}$ carrying a PC reporter. Mice were infected with *S. Typhimurium and* sacrificed at 3 d.p.i. Sections were stained for tdT, lysozyme, and PCNA. Arrows point to $PCNA^+tdT^+$ cells. Scale bar, 50 µm.

© The Author(s)
